# Rapid HPLC-Based ADME Profiling of Novel Donepezil and Rivastigmine Analogues for Potential Application in Alzheimer’s Disease

**DOI:** 10.1021/acsomega.6c07555

**Published:** 2026-10-06

**Authors:** Stefan Simić, Rosalba Leuci, Marianne Pirker, Predrag Kalaba, Judith Wackerlig-Damle, Luca Piemontese, Thierry Langer

**Affiliations:** † Department of Pharmaceutical Sciences, 27258 University of Vienna, Josef-Holaubek-Platz 2, 1090 Vienna, Austria; ‡ Vienna Doctoral School of Pharmaceutical, Nutritional and Sport Sciences, University of Vienna, Josef-Holaubek-Platz 2, 1090 Vienna, Austria; § Department of Pharmacy—Pharmaceutical Sciences, 9295 University of Bari Aldo Moro, via E. Orabona 4, 70126 Bari, Italy; ∥ 56369 The Florey Institute of Neuroscience and Mental Health, 30 Royal Parade, Parkville, Victoria 3052, Australia

## Abstract

Multitarget ligands derived from established cholinesterase inhibitors offer a strategy for addressing the multifactorial pathology of Alzheimer’s disease, but biological potency must be considered together with developability. In this study, a rapid biomimetic high-performance liquid chromatography-based workflow was used to characterize key absorption, distribution, metabolism and excretion-related properties of 26 donepezil- and rivastigmine-derived hybrid compounds, including plasma stability, plasma protein binding, membrane affinity, biomimetic distribution parameters, blood–brain barrier permeability, intestinal absorption, experimental log *P* and aqueous solubility. Plasma stability was reassessed using first-order regression across seven time points with 95% confidence intervals. Most profiles showed limited degradation during the 24 h experiment; accordingly, many apparent half-lives required extrapolation and are interpreted as comparative stability parameters rather than estimates of *in vivo* persistence. Human serum albumin and α1-acid glycoprotein binding was generally high, emphasizing the need to balance protein binding with the unbound fraction, while immobilized artificial membrane-based models predicted central nervous system-positive classification and high intestinal absorption across the series. Importantly, the blood brain barrier permeability and CNS-distribution estimates are derived from biomimetic models and should be regarded as predictive descriptors that require confirmation in dedicated *in vivo* pharmacokinetic studies. Solubility was more variable and emerged as an important differentiating property, with compound (**26**) showing the most favorable profile. An exploratory desirability-based ADME score placed compounds (**15**), (**8**), (**26**), (**4**), (**10**) and (**24**) in the highest tier. Integration with previously reported cholinesterase, fatty acid amide hydrolase and monoamine oxidase activities, which were not remeasured in the present study, further refined lead selection: (**24**) provided the clearest potency-developability compromise and was prioritized as the principal lead, whereas (**26**) emerged as a developability-oriented colead. (**3**) and (**18**) remained potency-driven secondary candidates. Overall, combining biomimetic ADME profiling with existing pharmacological evidence provides a transparent framework for early lead prioritization and supports confirmatory pharmacokinetic, exposure and *in vivo* efficacy studies.

## Graphical abstract

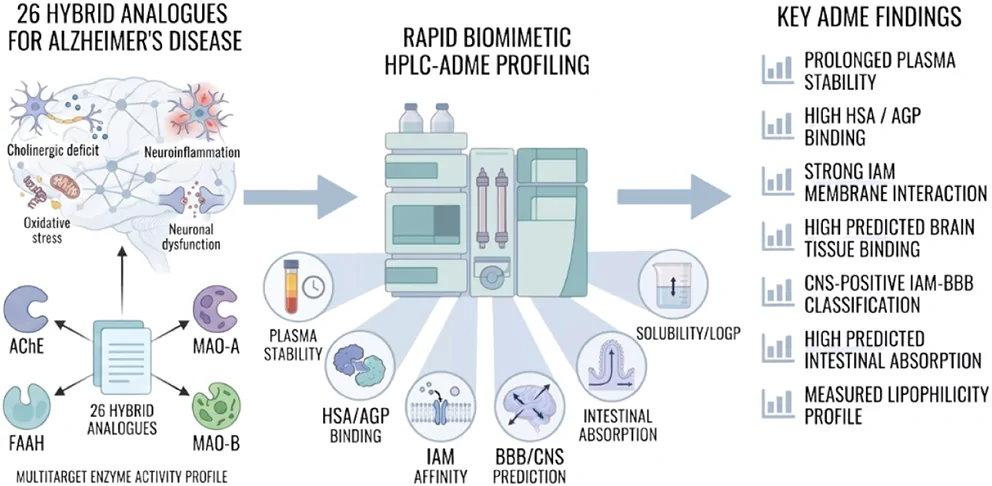

## 1 Introduction

Alzheimer’s disease (AD) is a progressive neurodegenerative disorder characterized by memory loss and broader cognitive decline, including impairments in language, learning, visuospatial abilities, reasoning, and behavior.^1^ The progressive decline in cognitive function can become sufficiently severe to impair daily activities. AD is the most common form of dementia, accounting for at least two-thirds of cases among individuals aged 65 years and older.^1,2^ With over 50 million people currently affected, and numbers projected to triple by 2050, AD represents one of the most pressing global health challenges.^2–4^ Despite decades of extensive research, the precise etiology of AD remains inconclusive, involving a complex interplay between amyloid-beta aggregation, tau pathology, oxidative stress, mitochondrial dysfunction, neuroinflammation, and impaired glucose metabolism.^5,6^ This lack of clear etiology and clear disease mechanism limits the efficacy of single-target drug development efforts and has driven a shift toward multitarget-directed ligand (MTDL) strategies, which aim to simultaneously modulate multiple pathological processes.^4,7^

Among currently available pharmacological treatments, donepezil and rivastigmine are two of the most widely prescribed agents for the symptomatic management of Alzheimer’s disease.^4^ These agents act by increasing synaptic levels of acetylcholine, a neurotransmitter that is significantly depleted in patients with AD. Donepezil selectively inhibits acetylcholinesterase (AChE), whereas rivastigmine inhibits both AChE and butyrylcholinesterase (BChE), which may provide additional benefits in certain aspects of cognitive function.^8–14^ Despite their widespread clinical use, donepezil and rivastigmine provide only modest symptomatic benefits and are associated with adverse effects such as gastrointestinal disturbances, bradycardia, and hepatotoxicity (particularly for rivastigmine), highlighting the need for improved cholinesterase inhibitors with optimized pharmacokinetic and safety profiles.^15^

Multitarget-directed ligands have therefore emerged as a strategy for addressing the multifactorial nature of AD. The compounds investigated here were designed to combine cholinesterase inhibition with additional disease-relevant activities, including inhibition of fatty acid amide hydrolase (FAAH).^16,17^ FAAH is the principal hydrolytic enzyme responsible for terminating signaling by the endocannabinoid anandamide; increased FAAH expression or activity has been reported in AD-related biological samples, and pharmacological or genetic FAAH inhibition can enhance endocannabinoid tone, modulate microglial activation, reduce pro-inflammatory signaling, promote amyloid-β phagocytosis, and improve synaptic or cognitive outcomes in experimental AD models.^18–22^ These findings support FAAH as a mechanistically relevant, although still preclinical target for mitigating neuroinflammation and synaptic dysfunction in AD. The selected hybrids were previously shown to inhibit AChE, BChE, and FAAH and, in some cases, to provide antioxidant or metal-chelating activity, thereby supporting their selection as multitarget candidates.^16,17^

The development of effective AD therapeutics requires a thorough understanding of pharmacokinetics (PK), including absorption, distribution, metabolism, and excretion (ADME) properties. Key parameters include plasma stability, plasma protein binding (PPB), volume of distribution (*V*_d_), and brain tissue penetration.^23–29^ Plasma stability assesses a compound’s chemical integrity in the bloodstream; instability can lead to reduced efficacy or rapid clearance. PPB, particularly to human serum albumin (HSA) and α1-acid glycoprotein (AGP), influences the unbound drug fraction available for activity.^26–28^ *V*_d_ indicates the extent of drug distribution into tissues and, along with clearance rates, determines the drug’s half-life and exposure. For central nervous system (CNS) therapeutics, brain tissue distribution is critical, as it dictates the drug concentration at the site of action.^27–29^ However, experimental determination of these PK parameters typically requires multiple specialized *in vitro* and *in vivo* assays that are time-consuming, costly, and technically demanding, often limiting their routine application in early stage drug discovery, particularly in smaller academic settings. To efficiently model these pharmacokinetic parameters, biomimetic chromatography has emerged as a powerful high-throughput alternative to animal studies. This technique uses high-performance liquid chromatography (HPLC) columns modified with HSA, AGP, and immobilized artificial membrane (IAM) phases to simulate biological environments.^30^

IAM columns provide experimental descriptors of phospholipid interaction, passive membrane partitioning, blood–brain barrier (BBB) permeability and intestinal absorption, while HSA and AGP columns estimate affinity for the principal plasma drug-binding proteins. These biomimetic approaches, combined with chromatographic and computational models, provide a rapid and resource-efficient framework for early compound prioritization. In this study, reported HPLC-based biomimetic methods were applied to characterize the pharmacokinetic and physicochemical profiles of novel MTDLs developed for AD.^30–34^

## 2 Materials and Methods

### 2.1 Test and Standard Compounds

In this study, 26 hybrid compounds were selected based on their published cholinesterase inhibition, antioxidant properties, and neuroprotective effects. These compounds originate from two distinct chemical frameworks, both designed for a multitarget approach to AD therapy. The first set (23 analogues) comprises rivastigmine-based hybrids (Table 1), which were developed to enhance the dual inhibition of acetylcholinesterase (AChE) and butyrylcholinesterase (BChE) while also incorporating antioxidant functionalities to counteract oxidative damage in the brain. The second set (3 analogues) consists of donepezil-like hybrids (Table 2), which combine the cholinergic enhancement properties of donepezil with its neuroprotective and redox-active characteristics, thereby targeting both cholinergic deficit and oxidative stress-related neurotoxicity.^16,17^

**Table 1:**
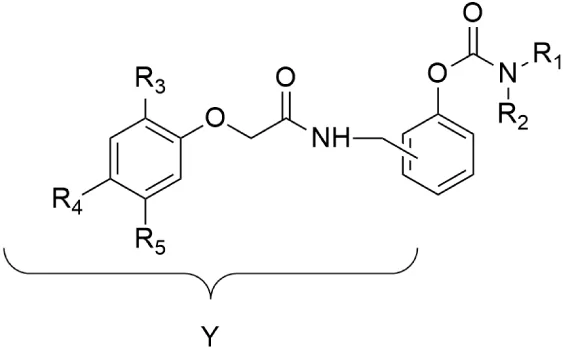
Structures and Previously Reported Biological Activities of the First Set of Analyzed Rivastigmine Analogues, as Reported By Leuci et al.^16^ ^,^ ^a^

compound	Y	R_1_	R_2_	R_3_	R_4_	R_5_	*h*AChE	*eq*BChE	*h*FAAH	*h*MAO-A	*h*MAO-B
(**1**)	*p*-	–CH_2_CH_3_	–CH_3_	–NO_2_	–Cl	–H	55 ± 4%^b^	59 ± 2%^b^	32.2 ± 8.37	44 ± 5%^b^	2.47 ± 0.10
(**2**)	*o*-	–CH_2_CH_3_	–CH_2_CH_3_	–H	–Ph	–H	4.83 ± 0.02	61 ± 3%^b^	15.3 ± 1.76	36 ± 3%^b^	9.44 ± 1.19
(**3**)	*m*-	–CH_3_	–CH_3_	–NO_2_	–Cl	–H	0.036 ± 0.007	2.76 ± 0.43	3.56 ± 0.09	6 ± 5%^b^	35 ± 3%^b^
(**4**)	*m*-	–CH_3_	–CH_3_	–H	–Cl	–H	45 ± 5%^b^	52 ± 1%^b^	3.35 ± 0.24	n.a.	49 ± 8%^b^
(**5**)	*m*-	–CH_3_	–CH_3_	–H	–Ph	–H	52 ± 9%^b^	53 ± 3%^b^	10.7 ± 7.20	n.a.	32 ± 4%^b^
(**6**)	*m*-	–CH_2_CH_3_	–CH_3_	–H	–Ph	–H	39 ± 9%^b^	60 ± 1%^b^	14.3 ± 4.96	n.a.	6.86 ± 0.90
(**7**)	*m*-	–CH_2_CH_3_	–CH_3_	–NO_2_	–Cl	–H	51 ± 2%^b^	45 ± 1%^b^	5.80 ± 2.69	n.a.	6.66 ± 1.05
(**8**)	*m*-	–CH_2_CH_3_	–CH_3_	–H	–Cl	–H	40 ± 4%^b^	33 ± 1%^b^	9.93 ± 8.12	27 ± 16%^b^	36 ± 9%^b^
(**9**)	*m*-	–CH_2_CH_3_	–CH_3_	–NO_2_	–H	–F	6.59 ± 1.63	60 ± 2%^b^	6.29 ± 3.31	7 ± 5%^b^	5.98 ± 1.64
(**10**)	*o*-	–CH_2_CH3	–CH_3_	–H	–Cl	–H	33 ± 4%^b^	54 ± 1%^b^	6.93 ± 0.17	35 ± 5%^b^	13 ± 4%^b^
(**11**)	*o*-	–CH_2_CH_3_	–CH_3_	–H	–Ph	–H	32 ± 1%^b^	1.69 ± 0.35	1.40 ± 0.25	48 ± 3%^b^	1.71 ± 0.75
(**12**)	*o*-	–CH_2_CH_3_	–CH_3_	–NO_2_	–H	–F	2.11 ± 0.16	61 ± 5%^b^	8.09 ± 1.71	43 ± 1%^b^	4.52 ± 1.06
(**13**)	*p*-	–CH_2_CH_3_	–CH_2_CH_3_	–H	–Cl	–H	4.80 ± 0.41	28 ± 2%^b^	17.2 ± 5.77	30 ± 3%^b^	52 ± 3%^b^
(**14**)	*m*-	–CH_2_CH_3_	–CH_2_CH3	–NO_2_	–H	–F	4.95 ± 0.98	62 ± 1%^b^	30.0 ± 10.1	23 ± 5%^b^	55 ± 1%^b^
(**15**)	*p*-	–CH_2_CH_3_	–CH_3_	–H	–Cl	–H	4.44 ± 0.86	57 ± 1%^b^	21.5 ± 0.44	17 ± 5%^b^	20 ± 5%^b^
(**16**)	*p*-	–CH_2_CH_3_	–CH_2_CH_3_	–H	–Ph	–H	5.13 ± 0.79	17 ± 5%^b^	22.8 ± 6.93	34 ± 3%^b^	43 ± 4%^b^
(**17**)	*o*-	–CH_2_CH_3_	–CH_3_	–NO_2_	–Cl	–H	4.76 ± 0.51	46 ± 5%^b^	14.2 ± 0.49	n.a.	21 ± 8%^b^
(**18**)	*m*-	–CH_3_	–CH_3_	–NO_2_	–H	–F	0.014 ± 0.006	1.68 ± 0.40	8.17 ± 3.77	n.a.	41 ± 8%^b^
(**19**)	*p*-	–CH_2_CH_3_	–CH_3_CH_2_	–NO_2_	–Cl	–H	2.86 ± 0.13	50 ± 3%^b^	39.8 ± 6.54	49 ± 3%^b^	2.93 ± 0.39
(**20**)	*o*-	–CH_2_CH_3_	–CH_3_CH_2_	–NO_2_	–H	–F	3.00 ± 0.72	39 ± 3%^b^	39.7 ± 9.36	11 ± 5%^b^	56 ± 5%^b^
(**21**)	*p*-	–CH_3_CH_2_	–CH_3_CH_2_	–NO_2_	–H	–F	4.33 ± 0.19	49 ± 2%^b^	n.a.	21 ± 5%^b^	1.91 ± 0.98
(**22**)	*p*-	–CH_3_	–CH_3_	–H	–Cl	–H	4.26 ± 0.45	59 ± 2%^b^	15.9 ± 2.73^c^	13 ± 4%^b^	38 ± 5%^b^
(**23**)	*p*-	–CH_3_	–CH_3_	–NO_2_	–Cl	–H	1.56 ± 0.37	2.11 ± 0.49	20.2 ± 6.65^c^	41 ± 3%^b^	52 ± 5%^b^

a *h*AChE = human acetylcholinesterase; *eq*BChE = equine butyrylcholinesterase; *h*FAAH = human fatty acid amide hydrolase; *h*MAO-A/B = human monoamine oxidase A/B.

b % inhibition at 10 μM; n.a. = not active; n.d. = not determined.

c Values are mean ± standard error of the mean (SEM) (*n* = 3).

**Table 2:**
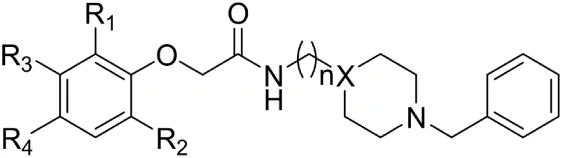
Structures and Previously Reported Biological Activities of the Second Set of Analyzed Donepezil-Like Hybrids, as Reported By Brunetti et al.^17^

compound	X	n	R_1_	R_2_	R_3_	R_4_	*h*AChE	*eq*BChE	*h*FAAH	*h*MAO-A	*h*MAO-B
(**24**)	–N	2	–H	–NO_2_	–H	–Cl	0.0008 ± 0.0002	1.41 ± 0.27	41.6 ± 7.4^a^	n.d.	n.d.
(**25**)	–N	2	–H	–NO_2_	–F	–H	0.0028 ± 0.0002	5.01 ± 0.12	49.6 ± 12.7^a^	n.d.	n.d.
(**26**)	–CH	1	–H	–H	–H	–Cl	1.68 ± 0.14	5.61 ± 0.23	20.9 ± 0.6^a^	n.d.	n.d.

a n.d. = not determined

Standard compounds (Tables 1 and 2, rivastigmine, donepezil) and chemicals for the plasma protein binding (PPB) and IAM chromatography assay were purchased from Sigma-Aldrich (Schnelldorf, Germany), TCI Germany (Eschborn, Germany) and VWR (Vienna, Austria) in the highest available purity and from Gall Pharma (Judenburg, Austria) as pharmaceutical standards. Solvents for chromatography were purchased from Merck (Darmstadt, Germany) in liquid chromatography–mass spectrometry (LC-MS) or HPLC-grade, depending on the performed analyses.

Blank pooled human plasma containing citrate as the anticoagulant was purchased from Biowest (Nuaillé, France). Blank pooled Sprague–Dawley rat plasma containing dipotassium ethylenediaminetetraacetate (K_2_EDTA) as the anticoagulant was purchased from NeoBiotech (Nanterre, France). As both plasma matrices used in the study were commercially obtained, no human participants or animals were recruited or sampled by the authors and thus no additional ethical approval was required for this study.

The stock solutions for the investigated compounds, as well as all standards for the PPB chromatography evaluation were prepared at a concentration of 1 mg/mL in dimethyl sulfoxide (DMSO). All investigated compounds exhibited a purity of ≥95%, as confirmed by the chromatograms provided in Figures S1–S26.

For plasma protein binding experiments, samples were prepared by combining the stock solutions with a mixture of 50 mM ammonium acetate buffer (pH 7.4) and 2-propanol (1:1, v/v), resulting in a final concentration of 0.5 mg/mL.

Samples in the experimental determination of chromatographic hydrophobicity index were prepared by mixing the stock solutions with a mixture of 50 mM ammonium acetate buffer (pH 7.4) and acetonitrile (1:1, v/v), thus resulting in a concentration of 0.5 mg/mL.

Samples for BBB penetration and intestinal absorption determination were prepared by mixing the working solutions with Dulbecco’s phosphate-buffered saline containing 20% acetonitrile (1:1, v/v) to obtain a concentration of 0.5 mg/mL.

The standard mixture used in determination of the experimental log *P* value was prepared by dissolving 2 mg of triphenylene and 0.2 mL of toluene in methanol to a final volume of 20 mL. Samples (concentration of 0.5 mg/mL) for HPLC analysis were obtained by mixing the stock solutions with the standard mixture (1:1, v/v).

For the solubility assay, threemedia solutions were used: JP1 (artificial gastric fluid), JP2 (artificial intestinal fluid) and a phosphate buffer (pH 7.4). The JP1 solution (pH 1.2; 35 mM NaCl and 84 mM HCl) was prepared by dissolving 2.0 g of NaCl and adding 7.0 mL of HCl, followed by dilution with water to a final volume of 1000 mL, while JP2 (pH 6.8; 50 mM phosphate buffer) was prepared by mixing 250 mL of 200 mM KH_2_PO_4_ with 118 mL of 200 mM NaOH and diluting to a final volume of 1000 mL with purified water. The phosphate buffer at pH of 7.4 (200 mM) was prepared by mixing 200 mM K_2_HPO_4_ and 200 mM KH_2_PO_4_ and subsequently adjusting the pH to 7.4.

### 2.2 Plasma Stability Assessment

Plasma stability was assessed by preparing each compound in triplicate. Pooled human plasma was thawed at room temperature, vortexed, and prewarmed at 37 °C for three minutes using a Mixmate shaker (Eppendorf, Hamburg, Germany) to ensure optimal reaction conditions. A volume of 50 μL of the stock solution (1 mg/mL) was added to 500 μL of blank plasma, yielding a final spiked plasma concentration of 90.9 μg/mL. The samples were vortexed for 20 s and incubated at 37 °C with gentle shaking at 500 rpm for various time points, specifically 0, 0.5, 1, 1.5, 2, 4, and 24 h. At each designated time point, 40 μL of the spiked plasma was withdrawn, and 100 μL of ice-cold methanol containing the internal standard (IS, donepezil) at a 1:2.5 v/v ratio was added to terminate the reaction and precipitate plasma proteins. The samples were vortexed thoroughly and centrifuged at 14,000 × g at 4 °C for 15 min. The resulting supernatant was carefully transferred into vials and stored at −20 °C until analysis.

At each incubation time, the triplicate analyte-to-internal-standard peak-area ratios were averaged. The mean response at each time point was then normalized to the corresponding mean response at 0 h, such that the normalized response represented the fraction of analyte remaining:

1 $$R_{t}=\frac{C_{t}}{C_{0}}$$

where *C*_0_ is the mean analyte-to-internal-standard response at 0 h and *C_t_* is the corresponding mean response at incubation time *t*. Apparent plasma degradation was evaluated using the following first-order kinetic model with the use of eq 2:

2 $$\ln\left(\frac{C_{t}}{C_{0}}\right)=a-k_{\mathrm{obs}}t$$

in which *a* is the fitted intercept and *k*_obs_ is the apparent first-order degradation rate constant. Thus, the *k*_obs_ corresponds to the negative slope of the regression line:

3 $$k_{\mathrm{obs}}=-\mathrm{Slope}$$

The apparent plasma half-life was calculated as described in eq 4:

4 $$t_{1/2}=\frac{\ln\left(2\right)}{k_{\mathrm{obs}}}$$

Linear regression was performed using the seven mean time-point responses obtained at 0, 0.5, 1, 1.5, 2, 4, and 24 h. Goodness of fit was assessed using the coefficient of determination (*R* ^2^). Two-sided 95% confidence intervals for *k*_obs_ were calculated from the standard error of the fitted slope using the Student’s *t* distribution with five degrees of freedom, as shown in eq 5:

5 $${\mathrm{CI}}_{95\%}\left(k_{\mathrm{obs}}\right)=k_{\mathrm{obs}}\pm t_{0.975,5}\cdot \mathrm{SE}\left(k_{\mathrm{obs}}\right)$$

When the lower confidence limit of *k*_obs_ was greater than zero, the corresponding 95% confidence interval for the apparent half-life was obtained by inverse transformation (eq 6):

6 $${\mathrm{CI}}_{95\%}\left(t_{1/2}\right)=\left[\frac{\ln\left(2\right)}{k_{\mathrm{obs},\mathrm{upper}}},\frac{\ln\left(2\right)}{k_{\mathrm{obs},\mathrm{lower}}}\right]$$

When the confidence interval for *k*_obs_ included zero, the upper confidence limit of *t*_1/2_ was considered unbounded. Such long half-life estimates should therefore be interpreted as apparent, extrapolated values with substantial uncertainty beyond the 24 h observation period. The statistical analyses were conducted using IBM SPSS Statistics 30 for Windows 11 (IBM Corp, Armonk, USA).

Chromatographic analysis was carried out using a Shimadzu Nexera XR ultrahigh-performance liquid chromatography (UHPLC) system, which included a DGU-20A5R degassing unit, two LC-20AD XR liquid chromatography pumps, a SIL-20A XR autosampler, a CTO-20AC column oven, and an SPD-M20A diode array detector set to extract chromatograms at 254 nm. The system was equipped with a Kinetex Phenyl-Hexyl column (2.6 μm, 2.1 mm × 50 mm, Phenomenex, US) along with a precolumn. The mobile phases consisted of LC-MS grade water containing 0.1% formic acid (solvent A) and LC-MS grade methanol containing 0.1% formic acid (solvent B). The applied gradient started with 20% solvent B from 0 to 1 min, increased to 95% B between 1 and 7 min, and was maintained at 95% B from 7 to 10 min for the washing phase. The column oven temperature was set to 37 °C, the injection volume was 10 μL, and the flow rate was maintained at 0.5 mL/min.

Since some of the compounds exhibited insufficient detection with the UHPLC system, the use of a high-resolution mass spectrometry was required. For liquid chromatography–high-resolution mass spectrometry (LC-HRMS), an Ultimate 3000 RSLC (rapid-separation liquid chromatography) system from Thermo Fisher Dionex (Germany) was coupled with a Bruker maXis electrospray ionization quadrupole time-of-flight (ESI-QTOF) mass spectrometer. The UHPLC system consisted of an SRD-3400 degassing unit, an RS pump, an RS autosampler, an RS column compartment, and an RS diode array detector operating in the range of 220 to 310 nm. Throughout the study, the same reverse-phase HPLC settings were applied to ensure reproducibility across all experiments, apart from the injection volume, which was set to 1 μL in the LC-HRMS analysis to prevent oversaturation of the detector.

### 2.3 Plasma Protein Binding (PPB) Analysis Using HSA and AGP Biomimetic HPLC Columns

All measurements were performed using a Shimadzu Prominence LC-20AD XR liquid chromatograph (Shimadzu Corporation, Tokyo, Japan) equipped with a photodiode-array (PDA) detector. The assay was carried out on two biomimetic chromatography columns: CHIRALPAK-HSA (5 μm, 3 mm × 50 mm, Daicel Inc., Tokyo, Japan) and CHIRALPAK-AGP (5 μm, 3 mm × 50 mm, Daicel Inc., Tokyo, Japan). The optimized gradient, as described previously,^35^ was applied as follows: 0–3 min: 0% B; 3–10 min: 30% B. Solvent B consisted of 2-propanol, while solvent A was a 50 mM ammonium acetate buffer adjusted to a physiological pH of 7.4 ± 0.05. The flow rate was set to 1.0 mL/min, and the column oven temperature was maintained at 30 °C to ensure optimal separation conditions. A lower flow rate was selected compared to the previously reported method^35^ to extend the column’s operational lifespan, as a higher flow rate would result in operation near the column’s maximum allowable pressure.

The dead time was determined by injecting uracil along (0.1 mg/mL) with the samples. For plasma protein binding (PPB) determination using the HSA and AGP columns, sample preparation involved mixing the stock solution with a 1:1 (v/v) mixture of 50 mM ammonium acetate buffer and 2-propanol, resulting in a final concentration of 0.5 mg/mL. The injection volume of the samples was 2 μL. All measurements were conducted in triplicate to ensure reproducibility.

For the calibration of the columns, the literature-reported %PPB values were converted into linear free energy-related log *k* values (logarithm of the apparent affinity constant) using eq 7, as described previously:^35^

7 $$\log k=\log\frac{\%\mathrm{PPB}}{101-\%\mathrm{PPB}}$$

The logarithmic values of the average retention times (log *t*_R_), determined from the triplicate injections of each compound, were plotted against the log *k* values. The slope and intercept of the resulting linear equation were then utilized to convert the gradient retention times into log *k* values for the analyzed compounds. Using these log *k* values, the estimated percentage of protein binding was subsequently calculated with the use of eq 8:

8 $$\%\;\mathrm{binding}=\frac{101\times 10^{\log\;k}}{1+10^{\log\;k}}$$

To calibrate the HSA column, a total of 13 standard compounds with well-characterized plasma protein binding (PPB) properties were selected. These compounds covered a PPB range from 19.0% (cimetidine) to 99.8% (diclofenac), ensuring a broad spectrum of binding affinities was represented. For the AGP column calibration, 7 standard compounds with known PPB values were used. These covered a PPB range from 3.2% (paracetamol) to 87.0% (nicardipine).

For the *in vivo* model estimation, the log *k* value derived from the HSA column had to be converted to log *K* so that a linear correlation with the linear free energy-related log *P* is established with the use of the following equation:^36^

9 $${\log K}_{\mathrm{HSA}}{=e}^{\log\;k(\mathrm{HSA})}$$

### 2.4 Immobilized Artificial Membrane (IAM) Chromatography Conditions

The samples for the determination of the chromatographic hydrophobicity index on immobilized artificial membrane (CHI_IAM_) were analyzed using a Shimadzu Prominence LC-20AD XR liquid chromatography system (Shimadzu Corporation, Tokyo, Japan) equipped with an IAM.PC.DD.2 column (3 mm × 4.6 mm, 12 μm; Regis Technologies, Morton Grove, IL, United States). Analyses were performed using a gradient elution with 50 mM ammonium acetate buffer (pH 7.40 ± 0.05) (solvent A) and acetonitrile (solvent B) (Merck KGaA, Darmstadt, Germany).

The flow rate was set to 0.5 mL/min, and the column oven was maintained at 25 °C. The injection volume was 2 μL. For retention time determination, detection wavelengths were set between 190 and 400 nm, depending on the maximum absorbance of each compound. The dead time was determined by injecting uracil (0.1 mg/mL) along with the sample. All measurements were performed in triplicate.

Five acetonitrile (B) concentrations were selected for each standard according to its retention behavior: chlorothiazide was analyzed at 5, 10, 15, 20, and 25% B; paracetamol, propyphenazone, propyl *p*-hydroxybenzoate, valerophenone, and 2,3-dichlorophenol at 15, 20, 25, 30, and 35% B; octanophenone at 30, 35, 40, 45, and 50% B; and tamoxifen at 50, 55, 60, 65, and 70% B. All measurements were performed in triplicate at each isocratic mobile-phase composition. The log *k*_IAM_ value was calculated using eq 10:

10 $$\log k_{\mathrm{IAM}}=\log\frac{t_{R}-t_{0}}{t_{0}}$$

where *t*_0_ is the dead time and *t*_R_ is the retention time. The log *k*_IAM_ values were plotted against the five different applied acetonitrile concentrations. The intercept (log *k*_*w*_) and slope (*S*) were used to calculate the isocratic hydrophobicity index (φ_0_) using eq 11:

11 $$\varphi_{0}=\frac{\log k_{w}}{S}$$

For gradient measurements, the method was applied as follows: 0% solvent B at 0–3 min, 0–70% solvent B, 3–5 min 70–80% solvent B, 5–7 min 80–100% solvent B, 7–8 min 100% solvent B.

The CHI_IAM_ value was calculated with slope *A* and intercept *B* according to the following equation:

12 $${\mathrm{CHI}}_{\mathrm{IAM}}{=At}_{R}+B$$

The CHI_IAM_ values obtained from gradient measurements were converted to log K_IAM_ and log *k*_IAM_ values using the following equations, as described by Hollósy:^36^

13 $${\log k}_{\mathrm{IAM}}=0.046\times {\mathrm{CHI}}_{\mathrm{IAM}}+0.42$$

14 $${\log K}_{\mathrm{IAM}}=0.29\times e^{\log k(\mathrm{IAM})}+0.7$$

### 2.5 Biomimetic *In Vivo* Distribution Models

Biomimetic *in vivo* models offer efficient and high-throughput alternatives to traditional *in vitro* and *in vivo* studies. Three key biomimetic chromatographic columns were utilized: HSA, AGP, and IAM chromatography.^30,36^

The volume of distribution (*V*_d_) is a fundamental pharmacokinetic parameter that describes the extent of drug distribution beyond plasma into body tissues. A biomimetic *in vivo* model HSA and IAM chromatography provides an efficient means of estimating *V*_d_, unbound volume of distribution (*V*_du_), and brain tissue binding (BTB) by mimicking plasma protein binding and membrane partitioning, two critical determinants of drug distribution.^33,36^

HSA chromatography is used to evaluate PPB, which directly influences the free drug fraction (*f*_u_). Since only unbound drug is available for distribution and pharmacological action, higher HSA binding leads to lower *f*_u_ and reduced *V*_d_, as the drug remains sequestered in plasma. Conversely, drugs with low HSA affinity exhibit higher *f*_u_, allowing extensive distribution into tissues, thereby increasing both *V*_d_ and *V*_du_.^36^

IAM chromatography mimics drug partitioning into phospholipid membranes, providing insights into tissue affinity and passive diffusion. A high IAM retention suggests strong interaction with lipid-rich tissues, leading to higher *V*_d_ as observed for lipophilic drugs accumulating in fat, cell membranes, or the central nervous system. In contrast, low CHI_IAM_ values indicate reduced membrane partitioning, resulting in lower *V*_d_ and limited tissue distribution.^35,36^

The combination of HSA and IAM data is valuable in estimating brain tissue binding (BTB), a crucial factor in CNS drug disposition. Since the brain consists of lipid-rich membranes, IAM retention correlates with drug affinity for brain tissue. Compounds with high CHI_IAM_ values typically exhibit strong BTB, indicating a higher likelihood of drug accumulation in the brain. Additionally, low plasma protein binding enhances free drug availability for CNS penetration, while high PPB can limit brain exposure despite high IAM affinity. This approach is particularly useful in optimizing CNS-active drugs, where both protein binding and lipid membrane interaction play a crucial role in drug disposition. The used models are shown in eqs 15–18.^30,36^

15 $$\log V_{d}=0.44\times {\log K}_{\mathrm{IAM}}-0.22\times {\log K}_{\mathrm{HSA}}-0.62$$

16 $$\log V_{\mathrm{du}}=0.23\times {\log K}_{\mathrm{HSA}}+0.43\times {\log K}_{\mathrm{IAM}}-0.72$$

17 $$\log k_{\mathrm{BTB}}=1.29\times {\log K}_{\mathrm{IAM}}+1.03\times {\log K}_{\mathrm{HSA}}-2.37$$

18 $$\%\mathrm{BTB}=\frac{100\times 10\log k_{\mathrm{BTB}}}{1+10\log k_{\mathrm{BTB}}}$$

### 2.6 Blood–Brain Barrier (BBB) Permeability and Human Intestinal Absorption (HIA) Based on IAM Chromatography

Chromatographic analyses were conducted on a Nexera XR UHPLC system (Shimadzu Corporation, Tokyo, Japan) fitted with an IAM.PC.DD.2 column (100 mm × 4.6 mm, 10 μm; Regis Technologies, Morton Grove, USA). The mobile phase was composed of LC-MS-grade acetonitrile and Dulbecco’s phosphate-buffered saline (DPBS). For the evaluation of the blood–brain barrier permeability, a mobile phase containing 20% acetonitrile and 80% DPBS was used, with the DPBS adjusted to pH 7.0 ± 0.05 using sodium hydroxide. For the intestinal absorption experiments, the DPBS pH was adjusted to 5.5 ± 0.05 with 2 M hydrochloric acid. The flow rate was kept at 1.0 mL/min, while the column oven was maintained at a temperature of 37 °C. The injection volume of the samples was 2 μL. The detection was performed with the use of diode array detector over a wavelength range between 190 and 400 nm.

The IAM retention, or capacity, factor (*k*_IAM_) was calculated according to eq 19:

19 $$k_{\mathrm{IAM}}=\frac{t_{R}-t_{0}}{t_{0}}$$

in which *t*_R_ denotes the retention time of the analyzed compound and *t*_0_ denotes the void time. The latter was determined by injecting 2 μL of uracil (0.5 mg/mL).

To consider the effect of molecular size on diffusion across the BBB, permeability estimates were adjusted using molecular weight–dependent correction factors, in line with previously reported IAM-based permeability models.^37–39^ The permeability descriptor (*P*_m_) was determined from a power-function correlation between *k*_IAM_ and molecular weight, as previously described and represented by the following equation:^40^

20 $$P_{m}=\frac{k_{\mathrm{IAM}}}{{\mathrm{MW}}^{4}}\times 10^{10}$$

A quantitative structure–activity relationship (QSAR) model was used to predict the percentage of human intestinal absorption (%HIA), following a previously reported multiple linear regression approach.^40^ The model includes physicochemical parameters with established relevance to intestinal drug absorption, including polar surface area (PSA), the logarithm of the partition coefficient (log *P*), and interaction terms based on experimentally obtained *k*_IAM_ values at pH 5.5.^39–43^ The physicochemical descriptors used for %HIA estimation were calculated using BIOVIA Dassault Systèmes Draw 2022 (Dassault Systèmes, San Diego, USA). The QSAR model is given by eq 21:

21 $$\%\mathrm{HIA}=\left(122.366\pm 4.527\right)-\left(0.417\pm 0.035\right)\times \mathrm{PSA}-\left(5.269\pm 1.584\right)\times \log P+\left(0.092\pm 0.019\right)\times \left(\mathrm{PSA}\times \log P\right)-\left(9.882\pm 0.641\right)\times \left({1/k}_{\mathrm{IAM}}\times \log P\right)$$

with *n* = 20, *R* = 0.988, *R* ^2^ = 0.975, *R* ^2^_adj_ = 0.969, *s* = 4.038, and *F* = 147.221

### 2.7 Experimental Log P Determination

Experimental log *P* determination was conducted on a Shimadzu Prominence LC-20AD XR liquid chromatography system (Shimadzu Corporation, Tokyo, Japan) equipped with a Shim-pack GIST C_18_ column (3 μm, 4.6 mm × 50 mm; Shimadzu Corporation, Tokyo, Japan). Chromatographic separation was performed using gradient elution with a mixture of 0.01 M phosphate buffer (pH 7.4) and methanol (1:1, v/v) as solvent A, and HPLC-grade methanol (Merck KGaA, Darmstadt, Germany) as solvent B. The proportion of solvent B was increased from 0% to 80% over a time span of 8 min, followed by an isocratic hold at 80% B for 4 min. The flow rate was set to 1.2 mL/min, and the column oven temperature was maintained at 40 °C. The injection volume was 5 μL for all samples at a concentration of 0.5 mg/mL. Retention times of the reference compounds and analytes were determined by peak integration at the wavelength of 254 nm. The log *P* values were calculated using toluene and triphenylene as reference standards according to eq 22, as previously described:^44^

22 $$\log P=[\left(\log P_{\mathrm{toluene}}-\log P_{\mathrm{triphenylene}}\right)\times t_{\mathrm{R,compound}}+t_{\mathrm{R,toluene}}\times \log P_{\mathrm{triphenylene}}-t_{\mathrm{R,triphenylene}}\times \log P_{\mathrm{toluene}}]/\left(t_{\mathrm{R,toluene}}-t_{\mathrm{R,triphenylene}}\right)$$

The *in silico* log *P* values were calculated using ACD/ChemSketch 2021.2.1 (ACD/Laboratories, Toronto, Canada), BIOVIA Dassault Systèmes Draw 2022 (Dassault Systèmes, San Diego, USA), ChemDraw 23 (Revvity Signals Software, Waltham, USA) for Windows 11 and SwissADME (Swiss Institute of Bioinformatics, Ecublens, Switzerland).^45^

### 2.8 Aqueous Solubility Assay

Samples for solubility determination were analyzed using a Shimadzu Prominence LC-20AD XR chromatography system (Shimadzu Corporation, Tokyo, Japan) fitted with a Shim-pack GIST C_18_ column (3 μm, 4.6 mm × 50 mm; Shimadzu Corporation, Tokyo, Japan) coupled to a suitable precolumn.

Analyses were performed using gradient elution with HPLC-grade water containing 0.1% phosphoric acid as solvent A and HPLC-grade acetonitrile (Merck KGaA, Darmstadt, Germany) as solvent B. The gradient started at 5% solvent B, increased linearly to 90% B over 3 min, and was maintained at 90% B for 2.5 min. The column oven temperature was set to 40 °C, the flow rate to 1.0 mL/min and the injection volume to 100 μL. Detection was performed using a diode array detector over a wavelength range of 210–400 nm.

For sample preparation, 50 μL of 1 mg/mL DMSO stock solution was transferred into 2 mL Eppendorf tubes, followed by the addition of 450 μL of the respective medium, resulting in a final volume of 500 μL. The samples were vigorously shaken for 1 h using an Eppendorf MixMate (Eppendorf, Hamburg, Germany) at 3000 rpm and subsequently centrifuged at 14,000 rpm for 10 min. A 50 μL aliquot of the supernatant was diluted with 100 μL of 50% acetonitrile, and 100 μL of the resulting solution was injected into the HPLC system.

For calibration, 50 μL of the 1 mg/mL stock solution was diluted with 450 μL of 50% acetonitrile to obtain a concentration of 0.1 mg/mL. Serial dilutions were then prepared to yield concentrations of 0.05, 0.01, and 0.001 mg/mL. The 1–100 μg/mL range was selected to bracket the expected supernatant concentrations and the lower reporting threshold of the discovery-stage solubility screen. Four nonequidistant calibration levels were used for the rapid comparative ranking.

Subsequently, the solubility (μg/mL) was determined based on the integrated chromatographic peak area using the following equation:^46^

23 solubility⁡=(concentrationoftwofold‐dilutedfiltrate)×2

The capacity factor (*k*′) was calculated with the use of eq 24:

24 $$k^{\prime }=\frac{t_{R}-t_{0}}{t_{0}}$$

where *t*_0_ was once again determined by injecting 5 μL uracil (0.5 mg/mL).

### 2.9 Chromatographic Quality Control

Analytical performance was monitored throughout the chromatographic sequences using an assay-specific check standard containing low, intermediate and high responses. The check standards were reinjected after every 15 sample injections. For the HSA- and AGP-based PPB assays, the standards represented low, intermediate and high protein-binding levels, whereas for the IAM-based BBB permeability and intestinal absorption assays, the standards represented early, intermediate and late chromatographic retention.

## 3 Results and Discussion

The complete study set consisted of 28 compounds, including 26 synthesized analogues and the two reference drugs, donepezil and rivastigmine.

### 3.1 Plasma Stability

The *in vitro* plasma stability experiment (Table 3) revealed that most derivatives exhibited a greater degradation rate in rat versus human plasma (Tables S1–S52), a trend consistent with the higher metabolic activity of rat plasma esterases and cytochrome P450 enzymes.^47,48^

**Table 3:** First-Order Kinetic Parameters for the Plasma Stability of Compounds (**1**)–(**26**) in Pooled Human and Rat Plasma^a^

compound	matrix	SI table	*k*_obs_ (h^–1^)	*R* ^2^	*p*	apparent *t*_1/2_ (h)	95% CI of *t*_1/2_ (h)	remaining at 24 h (%)
(**1**)	human	S1	0.00397	0.939	<0.001	175	135–247	89.5
(**1**)	rat	S2	0.01184	0.994	<0.001	59	54–64	75.3
(**2**)	human	S3	0.00432	0.585	0.045	160	82–4948	85.6
(**2**)	rat	S4	0.01348	0.979	<0.001	51	44–62	70.8
(**3**)	human	S5	0.00288	0.309	0.195	240	≥88*	87.6
(**3**)	rat	S6	0.00639	0.820	0.005	109	70–235	82.4
(**4**)	human	S7	0.00250	0.375	0.144	277	≥112*	90.7
(**4**)	rat	S8	0.00449	0.928	<0.001	154	117–227	88.4
(**5**)	human	S9	0.00080	0.485	0.082	868	≥398*	97.2
(**5**)	rat	S10	0.00787	0.678	0.023	88	49–425	78.6
(**6**)	human	S11	0.00165	0.510	0.072	421	≥198*	95.0
(**6**)	rat	S12	0.00175	0.376	0.143	395	≥159*	92.3
(**7**)	human	S13	0.00373	0.718	0.016	186	108–666	89.4
(**7**)	rat	S14	0.00943	0.843	0.004	74	49–146	74.9
(**8**)	human	S15	0.00474	0.568	0.050	146	≥73*	85.6
(**8**)	rat	S16	0.00369	0.680	0.022	188	105–885	90.6
(**9**)	human	S17	0.00214	0.587	0.044	324	165–8877	92.6
(**9**)	rat	S18	0.01647	0.991	<0.001	42	38–47	65.9
(**10**)	human	S19	0.00893	0.954	<0.001	78	62–104	78.7
(**10**)	rat	S20	0.01179	0.911	<0.001	59	43–92	72.8
(**11**)	human	S21	0.00149	0.653	0.028	466	253–2864	95.5
(**11**)	rat	S22	0.01461	0.996	<0.001	48	44–51	69.5
(**12**)	human	S23	0.00891	0.984	<0.001	79	68–91	80.6
(**12**)	rat	S24	0.01605	0.924	<0.001	43	32–65	64.6
(**13**)	human	S25	0.00372	0.653	0.028	186	101–1145	88.5
(**13**)	rat	S26	0.00525	0.901	0.001	132	96–214	86.8
(**14**)	human	S27	0.00642	0.823	0.005	108	70–232	84.5
(**14**)	rat	S28	0.00284	0.352	0.160	244	≥95*	89.9
(**15**)	human	S29	0.00869	0.961	<0.001	80	65–104	80.1
(**15**)	rat	S30	0.00924	0.969	<0.001	75	62–94	78.3
(**16**)	human	S31	0.01623	0.981	<0.001	43	37–51	66.4
(**16**)	rat	S32	0.04159	0.959	<0.001	17	13–22	35.6
(**17**)	human	S33	0.00421	0.763	0.010	165	100–458	88.2
(**17**)	rat	S34	0.01061	0.988	<0.001	65	58–75	76.3
(**18**)	human	S35	0.00408	0.788	0.008	170	106–421	89.0
(**18**)	rat	S36	0.01768	0.993	<0.001	39	36–43	64.4
(**19**)	human	S37	0.00308	0.746	0.012	225	134–682	92.0
(**19**)	rat	S38	0.00383	0.615	0.037	181	95–2008	88.7
(**20**)	human	S39	0.01466	0.990	<0.001	47	42–53	68.8
(**20**)	rat	S40	0.03879	0.987	<0.001	18	16–21	38.7
(**21**)	human	S41	0.00664	0.827	0.005	104	68–220	83.7
(**21**)	rat	S42	0.03773	0.998	<0.001	18	18–19	40.8
(**22**)	human	S43	0.00575	0.639	0.031	121	65–887	83.4
(**22**)	rat	S44	0.02357	0.997	<0.001	29	28–31	57.0
(**23**)	human	S45	0.01259	0.868	0.002	55	38–100	67.9
(**23**)	rat	S46	0.01113	0.979	<0.001	62	53–75	74.9
(**24**)	human	S47	0.00206	0.368	0.148	337	≥135*	91.4
(**24**)	rat	S48	0.01256	0.929	<0.001	55	42–81	72.2
(**25**)	human	S49	0.00243	0.372	0.146	285	≥114*	90.4
(**25**)	rat	S50	0.00397	0.551	0.056	175	≥86*	89.6
(**26**)	human	S51	0.00262	0.590	0.044	264	135–6425	92.3
(**26**)	rat	S52	0.00341	0.647	0.029	204	110–1345	89.1

a The apparent first-order degradation rate constant (*k*_obs_), coefficient of determination (*R* ^2^), regression *p* value, apparent plasma half-life (*t*_1/2_), corresponding 95% CI and percentage of parent compound remaining after 24 h are reported. Linear regression was performed using the mean normalized analyte response at seven incubation time points (0, 0.5, 1, 1.5, 2, 4, and 24 h). SI Table refers to the corresponding Supporting Information table containing the underlying plasma-stability data. For profiles in which the 95% CI of *k*_obs_ included zero, the upper confidence limit of the apparent half-life could not be estimated; in these cases, only the lower 95% confidence bound is reported (≥value) and the half-life should be interpreted as an uncertain extrapolation beyond the 24 h observation period.

The first-order fits showed variable goodness of fit across the 52 compound-matrix profiles (*R* ^2^ between 0.309 and 0.998). In total 49 apparent half-lives exceeded the 24 h observation period and were as such extrapolated beyond the experimental duration. Twenty-six estimates exceeded 120 h and represent strong extrapolations. For profiles with limited degradation, the 95% confidence intervals were broad and in several cases the upper half-life limit was unbounded because the confidence interval for *k*_obs_ included zero. These long values should therefore be interpreted as apparent ranking parameters indicating high plasma stability rather than precise kinetic half-lives.

Among the tested compounds, (**1**), (**2**), and (**16**) demonstrated substantial degradation in rat plasma. For example, (**16**) showed a decline from 100.0 ± 3.2% at 0 h to 66.4 ± 6.3% in human plasma, while in rat plasma, it dropped more substantially to 35.6 ± 7.6% after 24 h. Similarly, (**1**) retained 89.5 ± 1.4% of its initial concentration in human plasma but only 75.3 ± 9.4% in rat plasma. These findings suggest that these derivatives are more susceptible to enzymatic hydrolysis in rat plasma, leading to a significantly shorter half-life (*t*_1/2_ = 17 h; 95% confidence interval (CI): 13–22 h) for (**16**) in rat plasma versus 43 h (95% CI: 37–51 h) in human plasma. This characteristic could be beneficial for sustained therapeutic effects, reducing dosing frequency and prolonging drug exposure in clinical applications. However, their rapid degradation in plasma raises concerns about preclinical model suitability, as shorter half-lives may underestimate human pharmacokinetics.

Interestingly, a subset of compounds: (**8**), (**14**), and (**25**) exhibited the opposite trend, degrading more rapidly in human plasma than in rat plasma. For example, (**8**) retained 85.6 ± 0.6% of its initial concentration in human plasma but 90.6 ± 4.5% in rat plasma after 24 h, resulting in a longer *t*_1/2_ in rat plasma (188 h; 95% CI 105–885 h) than in human plasma (146 h; 95% CI: ≥73 h (n.d.)). Similarly, (**14**) showed a *t*_1/2_ of 108 h (95% CI: 70–232 h) in human plasma versus 244 h (95% CI: ≥95 h (n.d.)) in rat plasma, suggesting that this compound is more susceptible to enzymatic degradation in human plasma. These deviations from the expected trend could be attributed to species-specific differences in enzyme expression, substrate affinity, or plasma protein interactions. The presence of specific hydrolases or esterases in human plasma, which may be less active or absent in rat plasma, could contribute to these observed differences.^49,50^ Further studies, such as enzyme inhibition assays and metabolic profiling, are warranted to determine the exact enzymes responsible for the degradation of these derivatives.

In general, introduction of electron-withdrawing groups such as −NO_2_ and −Cl contributed to prolonged stability, as observed in (**3**), (**4**), (**7**), (**9**), and (**18**), where the human plasma half-life ranges between 170 and 324 h. Electron-withdrawing groups can reduce the electrophilic reactivity of hydrolytically susceptible carbonyl-containing motifs and may therefore decrease cleavage by plasma hydrolases, although the magnitude of this effect depends on the position of the substituent and the local electronic environment.^51,52^ Additionally, compounds containing bulky steric groups, such as (**5**) (868 h; 95% CI: ≥398 h (n.d.)) and (**6**) (421 h; CI: ≥198 h (n.d.)), exhibit significantly increased stability, suggesting that steric hindrance around metabolically susceptible bonds slows down enzymatic degradation.^52^ Conversely, compounds with less steric protection, such as (**16**) (43 h; 95% CI: 37–51 h), display rapid clearance, supporting the notion that accessibility to metabolic enzymes is a key determinant of half-life. Heterocyclic structures also appear to influence plasma stability, particularly in humans.^53^

The calculated *in vitro* plasma-stability half-lives ranged from 43 to 868 h in human plasma and from 17 to 395 h in rat plasma. (**16**) showed the shortest half-life in both species (43 h; 95% CI: 37–51 h in human plasma and 17 h; 95% CI: 13–22 h in rat plasma), whereas most derivatives were substantially more resistant to plasma degradation. Although these values exceeded the reported *in vivo* elimination half-life of rivastigmine (∼1 h), and several were numerically comparable to or greater than that of donepezil (79.5 ± 19.0 h), such comparisons should be interpreted cautiously because clinical half-life additionally reflects drug distribution, hepatic and extrahepatic metabolism, renal excretion, and other clearance processes.^11,14^ Thus, donepezil and rivastigmine are used here as qualitative pharmacological comparators rather than as directly equivalent plasma-stability controls. Among the derivatives, compounds (**10**) (78 h; 95% CI: 62–104 h), (**12**) (79 h; 95% CI: 68–91 h), (**16**) (43 h; 95% CI: 37–51 h), and (**20**) (47 h; 95% CI: 42–53 h) showed shorter human-plasma half-lives than the reported *in vivo* value for donepezil, while (**15**) showed an equivalent numerical value of 80 h (95% CI: 65–104 h).

From a drug-development perspective, resistance to degradation in plasma may reduce compound loss during circulation and improve the reproducibility of subsequent pharmacological assays.^54,55^ However, plasma stability alone does not establish bioavailability, systemic exposure, dosing frequency, duration of action, or in *vivo* clearance, because these properties also depend on absorption, distribution, hepatic and renal elimination, and the unbound drug fraction.^23,54,55^ Moreover, very high *in vitro* stability should not automatically be regarded as advantageous, as prolonged systemic persistence may increase the risk of accumulation, sustained toxicity, and drug–drug interactions if accompanied by slow *in vivo* clearance.^23,51^ While most compounds degraded faster in rat plasma, some demonstrated the opposite trend, suggesting species-specific metabolic pathways.^54^ Importantly, the present assay measures only the disappearance of the parent compound in isolated plasma under the specified incubation conditions. Comparisons with the clinically determined elimination half-lives of donepezil and rivastigmine are therefore provided solely as qualitative clinical context and should not be used to infer the *in vivo* persistence, systemic exposure, or dosing intervals of the hybrid compounds.

### 3.2 Plasma Protein Binding (PPB) Analysis

The plasma protein binding of the putative anti-Alzheimer’s compounds was investigated using biomimetic columns under physiological conditions (pH = 7.4). The calibration data for the HSA column is listed in Table 4. The selected compounds were chosen to cover a wide binding range, ranging from 19% (cimetidine) to 99.8% (diclofenac). The gradient retention times and log k exhibited a strong linear correlation (*R* ^2^ = 0.96), consistent with earlier findings.^35^ The retention times from the gradient elution were converted to log *k* using the following equation:

25 $$\log k=2.45\times \log t_{R}-0.13$$

**Table 4:** Calibration Set of Compounds Used for the HSA Column^a^

			log *k*		
compound	*t*_R_ ± SD	log *t*_R_	measured	literature	% PPB (literature)
acetylsalicylic acid	0.923 ± 0.007	–0.035	–0.22	–0.03	49
budesonide	3.075 ± 0.006	0.488	1.06	0.83	88
cimetidine	0.761 ± 0.015	–0.119	–0.42	–0.64	19
ciprofloxacin	0.973 ± 0.004	–0.012	–0.16	–0.18	40
diazepam	3.910 ± 0.002	0.592	1.32	1.63	98.7
diclofenac	6.087 ± 0.006	0.784	1.79	1.92	99.8
indomethacin	6.575 ± 0.012	0.818	1.87	1.69	99
ketoprofen	5.843 ± 0.102	0.767	1.74	1.69	99
modafinil	1.625 ± 0.014	0.211	0.38	0.17	60
nicardipine	4.036 ± 0.036	0.606	1.35	1.20	95
nizatidine	0.732 ± 0.019	–0.136	–0.46	–0.28	35
propranolol	2.337 ± 0.008	0.369	0.77	0.79	87
warfarin	4.637 ± 0.014	0.666	1.50	1.69	99

a SD – standard deviation; *t*_R_ – retention time; log *k* – logarithm of the apparent affinity constant.

Calibration of the AGP column was performed with standards listed in Table 5. The selected compounds have shown a strong correlation (*R* ^2^ = 0.96) between gradient retention times and log k values. The retention times obtained from the AGP column were converted to the log k values with the following equation:

26 $$\log k=2.71\times \log t_{r}-0.99$$

**Table 5:** Calibration Set of Compounds Used for the AGP Column^a^

			log *k*		
compound	*t*_R_ ± SD	log *t*_R_	measured	literature	% PPB (literature)
carbamazepine	3.359 ± 0.005	0.526	0.43	0.26	65
indomethacin	3.160 ± 0.002	0.500	0.36	0.09	56
nicardipine	3.989 ± 0.002	0.601	0.64	0.79	87
nizatidine	1.880 ± 0.004	0.274	–0.25	–0.24	37.1
paracetamol	0.637 ± 0.001	–0.196	–1.53	–1.49	3.2
propranolol	3.907 ± 0.010	0.592	0.61	0.76	86
warfarin	3.820 ± 0.004	0.582	0.59	0.67	83.2

a SD – standard deviation; *t*_R_ – retention time; log *k* – logarithm of the apparent affinity constant.

Listed in Table 6 are the biomimetic PPB data of the novel compounds compared to the well-established cholinesterase inhibitors donepezil and rivastigmine. The experimental results demonstrate that the compounds exhibit high plasma protein binding affinity, with human serum albumin binding values ranging from 92.9% to 97.8%. The plasma protein binding of the novel compounds is comparable to donepezil (94.6%), while it significantly exceeds rivastigmine (39.8%). Likewise, binding to AGP was consistently high for the new derivatives (72.8% to 94.8%) and donepezil (74.7%), in contrast to rivastigmine (25.1%).

**Table 6:** Biomimetic PPB Data of the Novel Compounds Compared to *In Silico* PPB Findings

	HSA				AGP						
compound	*t*_R_ ± SD	log *t*_R_	log *k*	%PPB	*t*_R_ ± SD	log *t*_R_	log *k*	%PPB	preADMET	deepPK	ADMETlab 3.0
(**1**)	4.095 ± 0.017	0.612	1.36	96.8	3.788 ± 0.022	0.578	0.58	79.8	99.4	87.5	98.4
(**2**)	4.573 ± 0.013	0.660	1.48	97.8	4.618 ± 0.012	0.664	0.81	87.4	89.4	87.5	97.8
(**3**)	3.719 ±0.002	0.570	1.26	95.8	4.607 ± 0.016	0.663	0.81	87.3	88.4	86.4	80.3
(**4**)	3.682 ± 0.013	0.566	1.25	95.6	4.221 ± 0.014	0.625	0.70	84.3	86.6	71.0	77.4
(**5**)	4.164 ± 0.007	0.620	1.38	97.0	4.391 ± 0.006	0.643	0.75	85.7	92.3	85.8	97.1
(**6**)	4.450 ± 0.007	0.648	1.45	97.6	4.548 ± 0.008	0.658	0.79	86.9	92.5	87.2	98.3
(**7**)	4.049 ± 0.003	0.607	1.35	96.7	4.734 ± 0.014	0.675	0.84	88.2	92.0	89.8	98.5
(**8**)	3.855 ± 0.039	0.586	1.30	96.2	4.413 ± 0.006	0.645	0.76	85.9	88.4	74.3	96.1
(**9**)	3.443 ± 0.008	0.537	1.18	94.8	4.885 ± 0.022	0.689	0.88	89.1	83.9	90.1	83.5
(**10**)	3.765 ± 0.019	0.576	1.28	95.9	4.482 ± 0.004	0.651	0.77	86.4	92.1	68.5	96.8
(**11**)	4.480 ± 0.007	0.651	1.46	97.6	4.461 ± 0.004	0.649	0.77	86.3	96.5	83.7	98.2
(**12**)	3.327 ± 0.075	0.522	1.14	94.2	5.465 ± 0.036	0.738	1.01	92.0	87.6	89.2	87.7
(**13**)	4.263 ± 0.004	0.630	1.41	97.2	3.908 ± 0.007	0.592	0.61	81.2	94.2	77.0	97.7
(**14**)	3.663 ± 0.024	0.564	1.25	95.6	5.548 ± 0.048	0.744	1.03	92.3	86.8	93.6	95.3
(**15**)	4.020 ± 0.003	0.604	1.35	96.6	3.781 ± 0.002	0.578	0.57	79.7	94.8	73.1	95.7
(**16**)	4.395 ± 0.004	0.643	1.44	97.5	4.513 ± 0.012	0.654	0.78	86.7	96.6	88.2	98.4
(**17**)	3.952 ± 0.004	0.597	1.33	96.5	4.923 ± 0.012	0.692	0.88	89.3	97.1	87.4	98.7
(**18**)	3.070 ± 0.001	0.487	1.06	92.9	4.605 ± 0.012	0.663	0.81	87.3	79.3	86.9	62.6
(**19**)	3.948 ± 0.025	0.596	1.33	96.4	3.780 ± 0.007	0.577	0.57	79.7	65.6	99.1	98.7
(**20**)	3.599 ± 0.023	0.556	1.23	95.4	3.299 ± 0.006	0.518	0.41	72.8	76.5	76.7	83.4
(**21**)	4.458 ± 0.008	0.649	1.45	97.6	4.024 ± 0.006	0.605	0.65	82.4	100.0	92.4	98.8
(**22**)	3.352 ± 0.037	0.525	1.15	94.4	6.341 ± 0.034	0.802	1.18	94.8	89.4	92.9	96.4
(**23**)	4.030 ± 0.030	0.605	1.35	96.7	3.733 ± 0.004	0.572	0.56	79.1	90.2	91.9	94.4
(**24**)	3.684 ± 0.019	0.566	1.25	95.7	3.766 ± 0.002	0.576	0.57	79.5	95.3	70.0	74.2
(**25**)	3.814 ± 0.020	0.581	1.29	96.1	3.655 ± 0.001	0.563	0.53	78.1	98.0	85.1	79.5
(**26**)	3.174 ± 0.032	0.502	1.09	93.5	3.800 ± 0.007	0.580	0.58	79.9	78.8	90.7	96.3
Donepezil	3.418 ± 0.005	0.534	1.17	94.6	3.418 ± 0.005	0.534	0.45	74.7	84.6	69.5	92.0
Rivastigmine	0.950 ± 0.020	–0.022	–0.19	39.8	1.546 ± 0.004	0.189	–0.48	25.1	26.4	10.0	34.7

HSA and AGP are major plasma drug-binding proteins, and their interactions can influence the unbound drug fraction, apparent volume of distribution, clearance, pharmacokinetic variability, and the concentration available for distribution into tissues.^26,56,57^ However, high PPB should not by itself be interpreted as evidence of prolonged circulation time or duration of action, because the pharmacokinetic consequences depend on unbound intrinsic clearance, tissue binding, extraction ratio, and changes in protein concentration.^58,59^ The consistently high HSA binding of the series is compatible with the compounds’ aromatic and hydrophobic character. HSA contains several ligand-binding cavities, including Sudlow sites I and II, in which drug association is governed by combinations of hydrophobic contacts, van der Waals forces, hydrogen bonding, electrostatic interactions, and, for aromatic ligands, π-associated interactions.^59–63^

The presence of strong electron-withdrawing substituents (−NO_2_, −Cl, and −F) significantly impacts binding affinity. Compounds (**1**), (**3**), (**7**), (**9**), and (**25**), all containing *p*- or *m*-NO_2_ groups, exhibited higher HSA binding (∼96–97%). This suggests that nitro groups enhance lipophilicity, allowing stronger hydrophobic and π-π interactions with HSA binding sites.^26,59,64^ Similarly, chlorine-substituted derivatives e.g., (**4**), (**10**), (**15**) maintained high PPB values, supporting previous studies where halogenated compounds displayed increased affinity for HSA due to halogen bonding and enhanced hydrophobic interactions.^65^ The ethyl (−CH_2_CH_3_) and methyl (−CH_3_) groups at R_1_ and R_2_ positions impact PPB by modulating the overall molecular lipophilicity and steric hindrance. Notably, compounds with ethyl groups e.g., (**2**), (**6**), (**16**), (2**1**) consistently exhibited higher binding to AGP (∼87–94%), potentially due to better accommodation within AGP’s hydrophobic pockets.^57,66,67^ Conversely, molecules with smaller methyl groups e.g., (**14**), (**18**), (**24**) showed slightly lower AGP binding (∼79–86%). Heterocyclic-containing compounds (**19**), (**20**) and (**26**) displayed slightly lower PPB values compared to fully aromatic derivatives, suggesting that the presence of nitrogen in the ring system reduces lipophilicity and alters binding site interactions.^68^ This could be due to disrupted π-π stacking within HSA’s Sudlow sites.^69^

The experimental PPB values obtained for donepezil and rivastigmine are in strong agreement with literature-reported data, confirming the reliability of our analytical approach. Donepezil exhibited a PPB of 94.6%, closely matching previously reported value of 96%.^70^ This high binding affinity is well-documented and suggests that donepezil remains extensively bound to plasma proteins, which can influence its pharmacokinetics and tissue distribution. In contrast, rivastigmine demonstrated a significantly lower PPB of 39.8%, aligning with literature values that place its binding around 40%.^71^ This lower affinity is consistent with its chemical structure, which lacks extensive hydrophobic and aromatic interactions that typically enhance protein binding. These findings confirm that the biomimetic HPLC-based methodology produces results that accurately reflect known pharmacokinetic properties, supporting the credibility of this experimental approach.

The comparison (Table S53) between experimental HSA binding and the *in silico* predictions showed useful but method-dependent agreement. The PPB values predicted by preADMET, deepPK and ADMETlab represent total plasma protein binding rather than binding specifically to HSA. Comparison between these end points is considered relevant because HSA is the most abundant plasma protein and is a major carrier of many drugs.^35,60^ Previous biomimetic chromatographic studies have similarly evaluated HSA-column retention by comparison with total PPB values obtained from plasma-based methods or published data sets. Accordingly, the present comparison was used to assess general binding trends rather than to imply direct equivalence between HSA binding and total PPB.^35,72^ Across the 28 compounds (Figure 1) and reference drugs, the squared Pearson correlations (*R* ^2^) were 0.76 for preADMET, 0.73 for deepPK, and 0.63 for ADMETlab, with mean absolute errors of 7.3, 12.6, and 5.7 percentage points, respectively. Thus, ADMETlab gave the lowest average absolute error, whereas preADMET provided the highest rank correlation. The remaining deviations emphasize that prediction accuracy depends on the chemical space used for model training and that experimental biomimetic measurements remain important for structurally complex, highly bound compounds.

**Figure 1:**
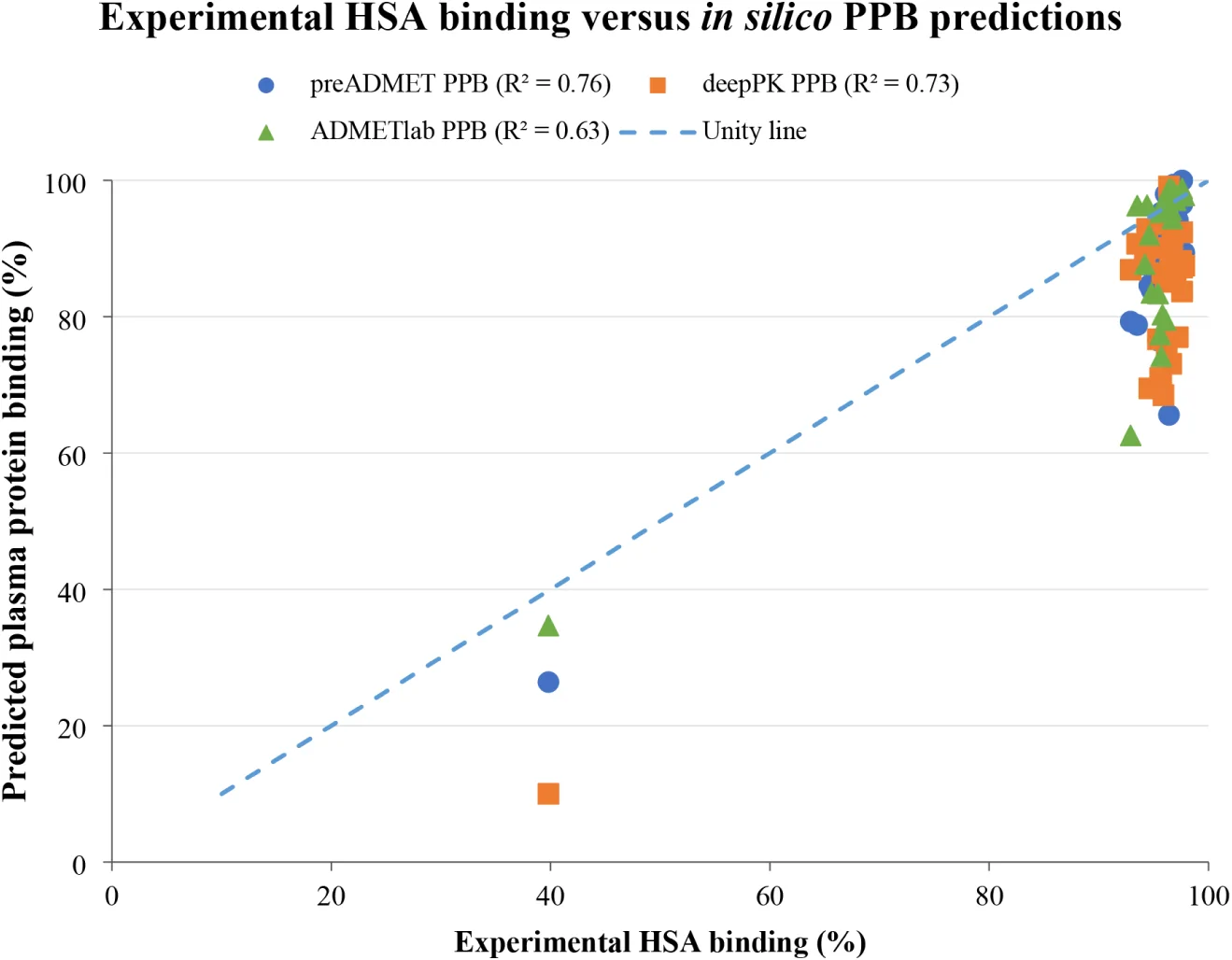
Comparison of the experimentally derived PPB data and *in silico* predicted data. Experimental HSA binding values are shown on the *x*-axis, and predicted total plasma protein binding values from preADMET, deepPK, and ADMETlab are shown on the *y*-axis. Individual observations are displayed as unconnected markers. The dashed unity line represents perfect agreement (y = x).

### 3.3 Phospholipid Binding Analysis by Immobilized Artificial Membrane (IAM) Chromatography

The phospholipid binding of the novel putative anti-Alzheimer’s compounds was assessed using an IAM column at physiological pH (7.4). The calibration data set (Table 7) was selected to encompass a broad range of CHI_IAM_ values, ensuring comprehensive coverage from the lowest to the highest CHI_IAM_ values. To determine the CHI_IAM_ values, a linear regression analysis was conducted between φ_0_ and the average retention time of three consecutive injections under gradient elution. As shown in eq 27, the slope (*A*) and intercept (*B*) obtained from this regression were then used to calculate CHI values for the compounds. The linear correlation between gradient retention times and isocratic φ_0_ values for standard compounds on the IAM column is described by the equation

27 $${\mathrm{CHI}}_{\mathrm{IAM}}=18.13\times t_{R}-69.67$$

with *n* = 8 and *R* ^2^ = 0.99.

**Table 7:** Set of Compounds Used for CHI Calibration on IAM Column at Physiological pH (7.4)

compound	*t*_R_ ± SD	*S*	log *k*_w_	CHI_IAM_
paracetamol	4.093 ± 0.004	–0.026	–0.130	4.9
chlorothiazide	4.154 ± 0.001	–0.046	0.253	5.5
propyphenazone	5.365 ± 0.005	–0.041	1.041	25.4
propyl *p*-hydroxybenzoate	5.855 ± 0.004	–0.049	1.808	36.7
valerophenone	5.945 ± 0.002	–0.049	1.892	38.7
2,3-dichlorophenol	6.148 ± 0.004	–0.045	1.875	41.5
octanophenone	6.425 ± 0.004	–0.060	2.972	49.4
tamoxifen	7.621 ± 0.004	–0.036	2.399	67.3

The CHI_IAM_ results reveal significant variations in membrane interaction potential, which is a determinant of drug absorption, distribution, and BBB permeability. Donepezil (CHI_IAM_ = 45.0) and rivastigmine (CHI_IAM_ = 39.0) serve as reference points, with donepezil exhibiting higher lipophilicity, which contributes to its prolonged CNS activity and strong BBB penetration.^73^

As shown in Table 8, compounds with higher CHI_IAM_ values, such as (**26**) (48.5), (**19**) (43.4), and (**2**) (41.6), exhibit increased lipophilicity and stronger membrane interaction, likely due to extended aromatic systems or larger alkyl substituents. (**26**) is more appropriately attributed to the combined effects of its aromatic surface, chloro substitution, tertiary amine-containing donepezil-like fragment, ionization state and molecular conformation; the presence of a nitrogen heterocycle alone does not predict higher lipophilicity.^30,74–76^ Nitro-containing analogues such as (**9**), (**12**), (**14**), and (**18**) generally showed lower experimental log *P* and moderate-to-low CHI_IAM_ values, consistent with the increased polarity and hydrogen-bond-accepting character of the NO_2_ group. Chlorine and fluorine should not be grouped with nitro substitution as having a uniform effect: chlorine commonly increases hydrophobic surface and polarizability, whereas fluorine produces smaller and highly context-dependent changes in lipophilicity. The experimental log *P* data therefore provide the most consistent interpretation of these IAM trends: additional phenyl substitution increased lipophilicity most strongly, chloro-substituted analogues generally occupied an intermediate range, and nitro/fluoro analogues without a phenyl substituent tended to be less lipophilic.^75–79^

**Table 8:** IAM Lipophilicity Data of the Novel Multi-Target Putative Anti-Alzheimer’s Agents

compound	*t*_R_ ± SD	CHI_IAM_	log *K*_IAM_	log *k*_IAM_
(**1**)	5.870 ± 0.001	36.7	3.093	2.110
(**2**)	6.138 ± 0.004	41.6	3.691	2.333
(**3**)	5.757 ± 0.006	34.7	2.877	2.016
(**4**)	5.724 ± 0.008	34.1	2.818	1.988
(**5**)	5.947 ± 0.006	38.1	3.251	2.174
(**6**)	6.030 ± 0.001	39.7	3.435	2.244
(**7**)	5.839 ± 0.004	36.2	3.031	2.084
(**8**)	5.823 ± 0.002	35.9	3.001	2.071
(**9**)	5.658 ± 0.009	32.9	2.704	1.933
(**10**)	5.835 ± 0.000	36.1	3.024	2.081
(**11**)	6.055 ± 0.006	40.1	3.492	2.265
(**12**)	5.646 ± 0.011	32.7	2.685	1.923
(**13**)	5.927 ± 0.006	37.8	3.209	2.158
(**14**)	5.745 ± 0.001	34.5	2.855	2.006
(**15**)	5.833 ± 0.006	36.1	3.020	2.080
(**16**)	6.110 ± 0.015	41.1	3.622	2.310
(**17**)	5.834 ± 0.003	36.1	3.022	2.080
(**18**)	5.550 ± 0.010	30.9	2.532	1.843
(**19**)	6.237 ± 0.006	43.4	3.950	2.417
(**20**)	5.947 ± 0.007	38.1	3.250	2.174
(**21**)	5.951 ± 0.004	38.2	3.260	2.178
(**22**)	5.747 ± 0.000	34.5	2.860	2.008
(**23**)	5.784 ± 0.003	35.2	2.926	2.038
(**24**)	5.732 ± 0.005	34.2	2.832	1.995
(**25**)	5.768 ± 0.006	34.9	2.898	2.025
(**26**)	6.521 ± 0.008	48.5	4.816	2.653
Donepezil	6.323 ± 0.002	45.0	4.192	2.488
Rivastigmine	5.993 ± 0.015	39.0	3.351	2.213

### 3.4 Biomimetic *In Vivo* Distribution Modeling

The biomimetic *in vivo* model data set (Table 9) provides valuable insights into the volume of distribution, unbound volume of distribution and brain tissue binding of the newly synthesized compounds in comparison to donepezil and rivastigmine. Donepezil (*V*_d_ = 9.25 L/kg) exhibits extensive tissue distribution, reflecting its high lipophilicity and strong membrane affinity, characteristics that facilitate its central nervous system (CNS) penetration. This *V*_d_ seems to be in line with the reported value of 11.8 ± 1.70 L/kg for a 5 mg dose and 11.6 ± 1.91 L/kg in literature.^70^ In contrast, rivastigmine (*V*_d_ = 4.70 L/kg) shows moderate distribution, likely due to its lower lipophilicity and weaker plasma protein binding, as confirmed with the reported literature value, which falls in the range between 2.8 and 5.5 L/kg.^80^ The majority of the newly synthesized compounds display lower *V*_d_ values (0.66–1.95 L/kg), suggesting that they may remain more confined to plasma and may have lower extravascular distribution compared to donepezil and rivastigmine.

**Table 9:** Biomimetic *In Vivo* Parameters of Novel Analogues Compared to Reference Drugs Donepezil and Rivastigmine

compound	log *k*_HSA_	log *K*_HSA_	CHI_IAM_	log *K*_IAM_	log *k*_IAM_	log *V*_d_	*V*_d_	log *V*_du_	log *k*_BTB_	%BTB
(**1**)	1.365	3.91	36.7	3.093	2.110	–0.12	0.76	1.51	1.76	94.6
(**2**)	1.482	4.40	41.6	3.691	2.333	0.04	1.09	1.88	2.17	95.6
(**3**)	1.263	3.53	34.7	2.877	2.016	–0.13	0.74	1.33	1.53	93.9
(**4**)	1.252	3.50	34.1	2.818	1.988	–0.15	0.71	1.30	1.48	93.7
(**5**)	1.383	3.99	38.1	3.251	2.174	–0.07	0.86	1.59	1.86	94.9
(**6**)	1.453	4.28	39.7	3.435	2.244	–0.05	0.89	1.74	2.02	95.3
(**7**)	1.353	3.87	36.2	3.031	2.084	–0.14	0.73	1.47	1.71	94.5
(**8**)	1.301	3.67	35.9	3.001	2.071	–0.11	0.78	1.41	1.64	94.3
(**9**)	1.181	3.26	32.9	2.704	1.933	–0.15	0.71	1.19	1.34	93.1
(**10**)	1.276	3.58	36.1	3.024	2.081	–0.08	0.84	1.40	1.63	94.2
(**11**)	1.460	4.31	40.1	3.492	2.265	–0.03	0.93	1.77	2.06	95.4
(**12**)	1.144	3.14	32.7	2.685	1.923	–0.13	0.74	1.16	1.29	92.8
(**13**)	1.408	4.09	37.8	3.209	2.158	–0.11	0.78	1.60	1.86	94.9
(**14**)	1.246	3.48	34.5	2.855	2.006	–0.13	0.74	1.31	1.50	93.8
(**15**)	1.345	3.84	36.1	3.020	2.080	–0.14	0.73	1.46	1.70	94.4
(**16**)	1.440	4.22	41.1	3.622	2.310	0.05	1.11	1.81	2.09	95.4
(**17**)	1.327	3.77	36.1	3.022	2.080	–0.12	0.76	1.45	1.68	94.4
(**18**)	1.059	2.88	30.9	2.532	1.843	–0.14	0.72	1.03	1.10	91.7
(**19**)	1.326	3.77	43.4	3.950	2.417	0.29	1.95	1.84	2.11	95.5
(**20**)	1.228	3.41	38.1	3.250	2.174	0.06	1.15	1.46	1.70	94.4
(**21**)	1.455	4.28	38.2	3.260	2.178	–0.13	0.74	1.67	1.94	95.1
(**22**)	1.152	3.17	34.5	2.860	2.008	–0.06	0.87	1.24	1.41	93.4
(**23**)	1.348	3.85	35.2	2.926	2.038	–0.18	0.66	1.42	1.65	94.3
(**24**)	1.253	3.50	34.2	2.832	1.995	–0.14	0.72	1.30	1.49	93.7
(**25**)	1.289	3.63	34.9	2.898	2.025	–0.14	0.72	1.36	1.57	94.0
(**26**)	1.094	2.99	48.5	4.816	2.653	0.84	6.95	2.04	2.18	95.6
donepezil	1.173	3.23	45.0	4.192	2.488	0.97	9.25	1.83	2.05	95.3
rivastigmine	–0.187	0.83	39.0	3.351	2.213	0.67	4.70	0.91	0.29	74.5

In terms of unbound volume of distribution (*V*_du_), donepezil (log *V*_du_ = 1.83) has a relatively high unbound fraction, meaning that a significant portion of the drug is freely available for distribution to tissues, including the CNS. Rivastigmine, on the other hand, shows a lower log *V*_du_ (0.91), indicating that despite its moderate *V*_d_, the fraction available for tissue penetration is smaller in comparison to donepezil. Among the new compounds, (**26**) (log *V*_du_ = 2.04) and (**19**) (log *V*_du_ = 1.84) exhibit the highest unbound volume, suggesting enhanced free drug availability, which is crucial for CNS penetration and pharmacological activity.

These results indicate that modulating plasma protein binding is key to optimizing CNS drug delivery, as compounds with lower *V*_d_ but higher *V*_du_ may achieve sufficient brain exposure while avoiding excessive peripheral distribution.^81,82^

The predicted brain tissue binding (BTB) values indicate extensive association of most compounds with brain lipids and other tissue constituents. Donepezil showed 95.3% BTB, rivastigmine 74.5%, and the synthesized compounds 92.8–95.6%, with (**26**), (**19**), and (**16**) giving the highest estimates.^83–86^ High BTB does not itself demonstrate BBB penetration or pharmacologically available brain exposure, because extensive tissue binding corresponds to a low unbound brain fraction. The BTB estimates therefore complement, but do not replace, the separate IAM-based passive BBB permeability model; definitive CNS exposure would require assessment of unbound brain-to-plasma partitioning and transporter effects.^87,88^ The somewhat lower BTB of (**18**) is consistent with its low experimental log *P* and increased polarity from nitro/fluoro substitution, but the effect should be attributed to the complete structure rather than to a universal electron-withdrawing-group rule.^83,85,86^

### 3.5 Blood–Brain Barrier (BBB) Permeability and Human Intestinal Absorption (HIA) Based on IAM Chromatography

The BBB permeability (Table 10) of the putative anti-Alzheimer’s analogues was estimated using IAM chromatography and compared with donepezil and rivastigmine as reference CNS-active drugs. All compounds investigated were classified as CNS-positive according to the IAM-derived *P*_m_ descriptor, with *P*_m_ values ranging from 2.81 to 18.66. These values were above the established CNS permeability threshold (1.51) and exceeded the *P*_m_ value of donepezil (*P*_m_ = 2.20). Most compounds also displayed higher predicted BBB permeability than rivastigmine (*P*_m_ = 4.46), indicating that the structural modifications introduced in the analogue series generally increased the membrane-interaction component relevant for passive BBB diffusion.

**Table 10:** BBB Permeability Estimation Based Upon Immobilized Artificial Membrane Chromatography^a^

compound	CNS	MW	*P*_m_	*k*_IAM_ [*n* = 3] ± CV [%]
(**1**)	+	421.8	10.66	33.75 ± 0.2
(**2**)	+	432.2	3.14	10.97 ± 2.5
(**3**)	+	407.8	6.19	17.12 ± 2.0
(**4**)	+	362.8	7.70	13.34 ± 2.1
(**5**)	+	404.5	18.65	49.91 ± 2.4
(**6**)	+	418.5	18.66	57.22 ± 0.5
(**7**)	+	421.8	9.71	30.75 ± 3.3
(**8**)	+	376.8	11.31	22.80 ± 1.2
(**9**)	+	405.4	4.29	11.58 ± 2.1
(**10**)	+	376.8	11.15	22.48 ± 1.5
(**11**)	+	418.8	12.31	37.86 ± 2.0
(**12**)	+	405.4	3.93	10.61 ± 1.9
(**13**)	+	390.9	11.70	27.30 ± 0.3
(**14**)	+	419.4	6.57	20.34 ± 1.1
(**15**)	+	376.8	12.41	25.02 ± 1.7
(**16**)	+	432.5	7.26	25.41 ± 0.4
(**17**)	+	421.8	8.79	27.84 ± 2.3
(**18**)	+	391.3	2.81	6.60 ± 2.8
(**19**)	+	435.9	5.25	18.94 ± 1.7
(**20**)	+	419.4	5.63	17.42 ± 3.2
(**21**)	+	419.4	7.61	23.55 ± 3.5
(**22**)	+	362.8	8.36	14.49 ± 2.3
(**23**)	+	407.8	6.95	19.22 ± 3.2
(**24**)	+	432.9	6.73	23.65 ± 0.6
(**25**)	+	398.5	3.15	7.93 ± 2.2
(**26**)	+	372.9	6.57	12.70 ± 0.7
donepezil	+	416.0	2.20	6.60 ± 0.6
rivastigmine	+	250.3	4.46	1.75 ± 0.2

a MW – molecular weight; *P*_m_ – coefficient of membrane permeability; *k*_IAM_ – retention factor on the IAM biomimetic column; CV – coefficient of variation calculated based on three separate measurements.

The first structural series, comprising compounds (**1**)–(**23**), based on a rivastigmine-related scaffold containing carbamate functionality and two aromatic regions connected through an ether/carbamate-type linker. Within this series, the highest *P*_m_ values were observed for compounds (**5**) and (**6**), with *P*_m_ values of 18.65 and 18.66, respectively. These compounds also showed the highest IAM retention factors, indicating strong interaction with the immobilized phospholipid stationary phase. Structurally, both compounds contain a phenyl substituent instead of more polar nitro-containing substitution patterns, which likely increases hydrophobic surface area and promotes membrane partitioning.

A similar favorable BBB profile was observed for compounds (**8**), (**10**), (**11**), (**13**), and (**15**), which showed *P*_m_ values between 11.15 and 12.41. These compounds combine moderate molecular weight with substantial aromatic and halogenated character, supporting passive membrane diffusion. In particular, −Cl- and −Ph-substituted analogues generally exhibited higher *k*_IAM_ and *P*_m_ values than compounds bearing −NO_2_ or −F substitution alone. This trend suggests that hydrophobic substituents increase phospholipid interaction and improve predicted BBB permeability, whereas polar nitro groups may partially reduce permeability by increasing polarity and hydrogen-bond accepting capacity.^38,89,90^

Compounds (**2**), (**9**), (**12**), (**18**), and (**25**) showed the lowest *P*_m_ values within the investigated set, although they remained above the CNS+ threshold. (**18**) had the lowest *P*_m_ among the rivastigmine analogue series (*P*_m_ = 2.81), while compounds (**2**), (**9**), and (**12**) showed *P*_m_ values of 3.14, 4.29, and 3.93, respectively. These values are still higher than donepezil but lower than, or close to, rivastigmine. The reduced permeability of these analogues appears to be associated with lower IAM retention due to the presence of more polar substituents, particularly nitro groups, which may reduce the ability of the compounds to partition into the lipid-like IAM stationary phase.^91^ This indicates that, although the general scaffold supports BBB permeation, the substitution pattern strongly modulates the extent of predicted passive permeability.^37,89^

Compared to donepezil and rivastigmine, the investigated compounds generally showed stronger IAM retention and higher calculated *P*_m_ values, indicating potential enhanced predicted passive BBB permeability. Donepezil contains a tertiary amine and two aromatic regions that support BBB penetration, but its *P*_m_ value within this data set was 2.20, which was lower than that of most analogues. Rivastigmine has a lower molecular weight and a tertiary amine-containing carbamate structure favorable for CNS permeability, but its *k*_IAM_ value was also relatively low compared with most synthesized compounds. The higher *P*_m_ values of the analogues can be attributed to their retained carbamate-like structural elements, expanded aromatic and hydrophobic domains, halogen substitution, and stronger phospholipid interaction, as reflected by the increased *k*_IAM_ values. These findings suggest that structural expansion relative to rivastigmine and increased aromatic surface area relative to donepezil improve predicted passive BBB permeability.^30,90,92^

IAM chromatography was selected as a rapid and reproducible screening method for comparing the BBB permeability potential of the analogues. The phosphatidylcholine-based stationary phase reflects interactions with membrane lipids and is suitable for estimating the passive membrane permeability.^37,40,74^ Importantly, the applied IAM approach was previously evaluated using 50 structurally diverse marketed drugs with documented positive or negative CNS penetration profiles and successfully differentiated CNS-permeable from nonpermeable compounds.^40^

Nevertheless, IAM chromatography does not reproduce the full biological complexity of the BBB, including active uptake and efflux transport, metabolism and protein binding.^40,90,93^ It was therefore used in the present study as an early stage comparative tool to rank the analogues relative to donepezil and rivastigmine, rather than as definitive evidence of *in vivo* brain exposure.

The human intestinal absorption (Table 11) of the investigated compounds was estimated using the IAM chromatography-based QSAR model and compared with donepezil and rivastigmine as reference drugs. Overall, all investigated analogues showed high predicted intestinal absorption, with %HIA values ranging from 81% to 100%. These values indicate favorable predicted oral absorption across the whole compound set. Importantly, all compounds showed higher predicted %HIA than rivastigmine (%HIA = 69%), while most compounds were also comparable to or higher than donepezil (%HIA = 86%). This ranking is consistent with literature data showing that donepezil generally exhibits high oral absorption, with reported relative intestinal absorption ranging between 95–100%, whereas rivastigmine shows lower and more variable systemic availability, although one study reported an absolute bioavailability of 72% for rivastigmine after oral administration.^11,94,95^ Thus, the model-derived values reflect the general literature trend in which donepezil displays more favorable intestinal absorption/systemic availability than rivastigmine.

**Table 11:** Human Intestinal Absorption Estimation Based on IAM Chromatography^a^

compound	%HIA	PSA	log *P*	PSA × log *P*	1/*k*_IAM_ × log *P*	1/*k*_IAM_	*k*_IAM_ ± CV [%]
(**1**)	91	113.69	3.33	379.04	0.13	0.04	24.88 ± 0.1
(**2**)	96	50.8	4.78	242.70	0.29	0.06	16.11 ± 0.3
(**3**)	88	113.69	2.99	339.39	0.24	0.08	6.37 ± 3.0
(**4**)	94	67.87	3.09	209.77	0.34	0.11	9.01 ± 0.1
(**5**)	97	67.87	3.94	267.73	0.12	0.03	39.89 ± 0.4
(**6**)	97	67.86	4.29	291.36	0.13	0.03	31.10 ± 0.5
(**7**)	100	113.69	3.33	379.04	0.17	0.05	21.94 ± 0.1
(**8**)	95	67.87	3.44	233.45	0.21	0.06	16.56 ± 0.3
(**9**)	87	113.69	2.88	326.87	0.32	0.11	9.01 ± 0.2
(**10**)	95	67.87	3.44	233.45	0.21	0.06	16.62 ± 0.4
(**11**)	96	67.87	4.29	291.41	0.26	0.06	16.14 ± 0.3
(**12**)	86	113.69	2.88	326.87	0.37	0.13	7.91 ± 2.2
(**13**)	97	67.86	3.79	257.08	0.11	0.03	33.41 ± 0.1
(**14**)	90	113.69	3.22	366.53	0.19	0.06	15.57 ± 0.3
(**15**)	96	67.86	3.44	233.41	0.17	0.05	18.33 ± 0.4
(**16**)	96	67.87	4.64	315.08	0.28	0.06	15.56 ± 0.4
(**17**)	91	113.69	3.33	379.04	0.17	0.05	20.32 ± 0.3
(**18**)	82	113.69	2.53	287.22	0.66	0.26	3.84 ± 1.1
(**19**)	89	113.68	3.68	418.66	0.55	0.15	6.54 ± 0.1
(**20**)	89	113.68	3.22	366.49	0.26	0.08	12.95 ± 2.5
(**21**)	90	113.69	3.22	366.53	0.19	0.06	17.70 ± 0.6
(**22**)	94	67.86	3.09	209.74	0.34	0.11	9.36 ± 0.2
(**23**)	88	113.69	2.99	339.39	0.24	0.08	12.96 ± 0.6
(**24**)	89	90.63	3.11	282.01	0.53	0.17	5.93 ± 0.2
(**25**)	81	90.63	2.45	221.80	1.08	0.44	2.26 ± 0.1
(**26**)	89	41.57	3.81	158.39	1.07	0.28	3.60 ± 0.7
Donepezil	86	38.77	4.57	177.17	1.28	0.28	3.52 ± 0.8
Rivastigmine	69	32.78	2.60	85.21	3.46	1.33	0.75 ± 0.7

a %HIA – percentage of human intestinal absorption; PSA – polar surface area; log *P* – logarithm of the partition coefficient; *k*_IAM_ – retention factor on the IAM biomimetic column; CV – coefficient of variation calculated based on three separate measurements.

The first structural series, compounds (**1**)–(**23**), consists of rivastigmine-related analogues containing carbamate functionality and substituted aromatic fragments. Within this series, the predicted %HIA values were consistently high, ranging between 82% and 100%. The highest predicted absorption was observed for (**7**) (%HIA = 100%), followed by (**5**), (**6**), and (**13**) (%HIA = 97%), and compounds (**2**), (**11**), (**15**), and (**16**) (%HIA = 96%). These compounds generally combine moderate-to-high lipophilicity with substantial IAM retention, suggesting favorable interaction with membrane-like environments while maintaining sufficient physicochemical balance for intestinal absorption.

The high predicted absorption of (**5**) and (**6**) is notable, as these compounds also showed the strongest IAM retention in the BBB permeability analysis. Their high log *P* values and hydrophobic aromatic substituents likely contribute to the strong membrane affinity.^39,96^

Compounds containing higher polar surface area values, particularly nitro-substituted analogues with PSA values around 113.69 Å^2^, generally showed slightly lower predicted %HIA values. Examples include compounds (**9**), (**12**), (**18**), (**19**), (**20**), (**21**), and (**23**), with predicted absorption values between 82% and 90%. (**18**) showed the lowest predicted %HIA within the rivastigmine analogue series (%HIA = 82%), which can be explained by its relatively high PSA and lower IAM retention. This trend is consistent with the final QSAR model, where polar surface area contributes negatively to intestinal absorption, reflecting the reduced passive permeability of more polar compounds.^97,98^

The role of lipophilicity also appears to be structure-dependent. Compounds with higher log *P* values, such as (**2**), (**6**), (**11**), and (**16**), maintained high predicted absorption, but the model does not treat increasing log *P* as universally beneficial. Rather, the interaction terms involving log *P*, PSA, and 1/*k*_IAM_ indicate that absorption depends on the balance between polarity, lipophilicity, and membrane interaction. Therefore, compounds with high log *P* but sufficient IAM retention and acceptable PSA values are predicted to remain well absorbed, whereas compounds combining increased polarity with weaker IAM retention show lower %HIA values.^99,100^

Compared with donepezil, most analogues showed similar or higher predicted intestinal absorption. Donepezil had a predicted %HIA value of 86%, with low PSA and high log *P*. Several analogues, including compounds (**1**)–(**17**) and (**19**)–(**24**), exceeded this value. This suggests that the structural modifications introduced into the rivastigmine-related scaffold generally preserve or improve predicted intestinal absorption relative to donepezil. Compared with rivastigmine, the difference was even more pronounced. Rivastigmine showed a predicted %HIA of 69%, whereas all new analogues were predicted above 80%. This improvement may be attributed to the increased hydrophobic and aromatic character of the analogues, which enhances interaction with the IAM stationary phase and supports passive membrane partitioning.

The donepezil-like hybrids, compounds (**24**)–(**26**), also showed high predicted absorption, although the values differed within this small set of compounds. (**24**) and (**26**) both showed %HIA values of 89%, while (**25**) showed the lowest value among the hybrid compounds (%HIA = 81%). The lower value of (**25**) may be associated with its lower *k*_IAM_ value and lower log *P*, indicating reduced membrane interaction compared to (**24**) and (**26**). Interestingly, (**26**) had the lowest PSA value among the hybrids, which may partly compensate for its modest *k*_IAM_ value and support its predicted absorption.^39,74^

These results support favorable passive absorption potential, but they should not be equated with oral bioavailability, which additionally depends on dissolution, gastrointestinal stability, transporter effects, and first-pass metabolism.^39,41–43,96^

The *V*_d_, *V*_du_, BTB, BBB permeability, and %HIA values reported are model-derived estimates rather than direct pharmacokinetic measurements. Their principal applicability to the present chemical series is comparative ranking, because all analogues were evaluated under identical chromatographic conditions and largely share related rivastigmine- or donepezil-derived scaffolds. Nevertheless, these models were developed from external calibration and/or training sets and their applicability domains may not fully encompass these hybrids, particularly compounds at the extremes of lipophilicity, ionization, molecular size, or protein and membrane affinity. The models primarily describe HSA and phospholipid interactions and passive partitioning—they do not account for active uptake or efflux transport, metabolism outside plasma, dissolution, first-pass metabolism, tissue-specific binding, or the dynamic biological barriers that determine systemic and brain exposure. Consequently, these predicted parameters should be interpreted as hypothesis-generating descriptors for early stage prioritization and require confirmation using orthogonal *in vitro* assays and, ultimately, *in vivo* pharmacokinetic studies.

### 3.6 Experimental Log P Determination

The experimentally determined HPLC log *P* values were compared with *in silico* predictions (Table 12) to evaluate the lipophilicity profile of the investigated compounds and its relevance for ADME and CNS-oriented drug design. Lipophilicity is one of the key physicochemical parameters in medicinal chemistry due to its effect on aqueous solubility, passive membrane permeability, plasma protein binding, tissue distribution, metabolic stability, and toxicity.^75–77^ Donovan and Pescatore described HPLC-based log *P* determination as a rapid and practical alternative to classical shake-flask measurements, particularly when only small amounts of material are available or when compound purity is limited, and emphasized that calculated log *P* values may deviate when structural fragments, connectivity patterns, or intramolecular interactions are not adequately represented in prediction databases.^44^ This is relevant for the present compound set, which contains multifunctional structures with aromatic rings, carbamate/urea-like motifs, tertiary amine-containing fragments, nitro groups, and halogen substituents.

**Table 12:** Experimental and *In Silico* calculated log *P* Values of the Investigated Analogues and the Reference Drugs^a^

		SwissADME						ChemDraw 23			
compound	experimental log *P* + SD	iLOGP	XLOGP	WLOGP	MLOGP	SILICO-IT	BIOVIA Draw 2022	Broto	Viswanadhan	Crippen	ACD/ChemSketch 2021.2.1
(**1**)	3.09 ± 0.003	2.89	3.32	3.24	1.87	0.96	3.33	2.78	Ø	Ø	3.06
(**2**)	4.21 ± 0.000	4.09	4.86	4.74	3.55	4.47	4.64	4.10	4.58	4.57	4.87
(**3**)	2.30 ± 0.012	2.49	2.95	2.85	1.64	0.56	4.36	2.14	Ø	Ø	2.53
(**4**)	1.99 ± 0.009	3.19	3.12	2.94	2.53	2.66	3.09	2.18	2.73	2.78	2.84
(**5**)	3.25 ± 0.007	3.69	4.12	3.96	3.13	3.66	3.94	2.99	3.9	3.9	3.81
(**6**)	3.67 ± 0.003	3.74	4.49	4.35	4.34	4.07	4.29	3.63	4.24	4.23	4.34
(**7**)	2.85 ± 0.018	2.90	3.32	3.24	1.87	0.96	3.33	2.78	Ø	Ø	3.06
(**8**)	2.83 ± 0.005	3.58	3.49	3.33	2.76	3.06	3.43	2.82	3.08	3.12	3.37
(**9**)	2.36 ± 0.009	2.72	2.79	3.15	1.76	0.74	2.87	2.30	Ø	Ø	2.18
(**10**)	2.94 ± 0.005	3.22	3.49	3.33	2.76	3.06	3.43	2.82	3.08	3.12	3.37
(**11**)	3.87 ± 0.005	3.68	4.49	4.35	3.34	4.07	4.29	3.63	4.24	4.23	4.34
(**12**)	2.41 ± 0.002	2.37	2.79	3.15	1.76	0.74	2.87	2.3	Ø	Ø	2.18
(**13**)	3.23 ± 0.002	3.70	3.86	3.72	2.98	3.47	3.78	3.28	3.42	3.46	3.9
(**14**)	1.86 ± 0.031	2.98	3.16	3.54	1.98	1.15	3.22	2.76	Ø	Ø	2.72
(**15**)	2.82 ± 0.010	3.46	3.49	3.33	2.76	3.06	3.43	2.82	3.08	3.12	3.37
(**16**)	4.06 ± 0.010	2.98	3.16	3.54	1.98	1.15	4.64	4.10	4.58	4.57	4.87
(**17**)	3.09 ± 0.003	2.39	3.32	3.24	1.87	0.96	3.34	2.78	Ø	Ø	3.06
(**18**)	1.87 ± 0.003	2.42	2.43	2.76	1.53	0.34	2.52	1.66	Ø	Ø	1.65
(**19**)	3.48 ± 0.005	3.13	3.69	3.63	2.09	1.36	3.68	3.24	Ø	Ø	3.59
(**20**)	2.64 ± 0.012	2.97	3.16	3.54	1.98	1.15	3.22	2.76	Ø	Ø	2.72
(**21**)	2.65 ± 0.007	3.26	3.16	3.54	1.98	1.15	3.22	2.76	Ø	Ø	2.72
(**22**)	3.00 ± 0.010	3.16	3.12	2.94	2.53	2.66	3.09	2.18	2.73	2.78	2.84
(**23**)	2.68 ± 0.005	2.81	2.95	2.85	1.64	0.56	2.98	2.14	Ø	Ø	2.53
(**24**)	3.57 ± 0.003	2.40	3.04	1.65	2.10	1.21	3.11	1.37	Ø	Ø	2.17
(**25**)	2.80 ± 0.010	2.87	2.41	0.99	1.61	0.56	2.44	0.76	Ø	Ø	1.69
(**26**)	3.41 ± 0.028	3.51	4.14	3.21	3.04	4.08	3.81	2.97	3.41	3.50	4.01
donepezil	3.93 ± 0.001	3.92	4.28	3.83	3.07	4.00	4.57	3.65	3.69	4.01	4.71
rivastigmine	2.14 ± 0.002	3.21	2.29	2.44	2.34	1.46	2.60	Ø	2.41	2.36	2.14

a Experimental log *P* – HPLC experimentally determined log *P*; SD – standard deviation based on three measurements; SwissADME, BIOVIA Draw, ChemDraw, and ACD/Chemsketch – *in silico* determined log *P* values.

The experimental log *P* values of the compound set ranged from 1.86 to 4.21, indicating that the investigated compounds occupy a moderate lipophilicity range. This is favorable from an ADME perspective because sufficient lipophilicity is required for membrane partitioning, but excessive lipophilicity is associated with reduced aqueous solubility, increased nonspecific binding, higher metabolic turnover, and increased attrition risk.^44,77^ Lipinski’s rule of five introduced log *P* ≤ 5 as one of the widely used empirical limits associated with oral drug-likeness.^101^ Later analyses further emphasized that lipophilicity should be carefully controlled during optimization, since increasing lipophilicity may improve potency or permeability but can simultaneously worsen solubility, selectivity, and safety-related properties.^30,75,76,102^ In this context, the fact that none of the investigated compounds exceeded an experimental log *P* of 5 suggests that the series remains within a physicochemical window compatible with further ADME profiling.

The comparative performance of the computational log *P* methods is summarized in Figure 2 using correlation strength, absolute prediction error, and data coverage. ChemDraw Crippen and Viswanadhan showed the strongest agreement with the experimental values, with Pearson correlation coefficients of 0.931 and 0.906 and mean absolute errors of 0.346 log *P* units. However, both methods were evaluated for only 14 compounds. Among the methods available for the complete data set of 28 compounds, ACD/ChemSketch provided the best overall performance, with a Pearson correlation coefficient of 0.812, an *R* ^2^ of 0.660, and a mean absolute error of 0.452 log *P* units. In contrast, SILICO-IT produced the largest prediction error. Therefore, ACD/ChemSketch provided the most representative agreement across the full compound set, whereas the stronger results obtained with Crippen and Viswanadhan should be interpreted as subset-specific. The complete statistical comparison is provided in Table S54. For CNS-directed compounds, passive BBB penetration requires sufficient membrane affinity, but successful CNS drug design generally depends on balancing controlled lipophilicity with molecular weight, polar surface area, hydrogen-bonding capacity and p*K*_a_ rather than relying on log *P* alone.^103–106^ Therefore, the experimentally observed log *P* range between 1.86 and 4.21 is consistent with a balanced CNS-relevant lipophilicity profile, especially when interpreted together with IAM retention, PSA, BBB permeability and intestinal absorption data.

**Figure 2:**
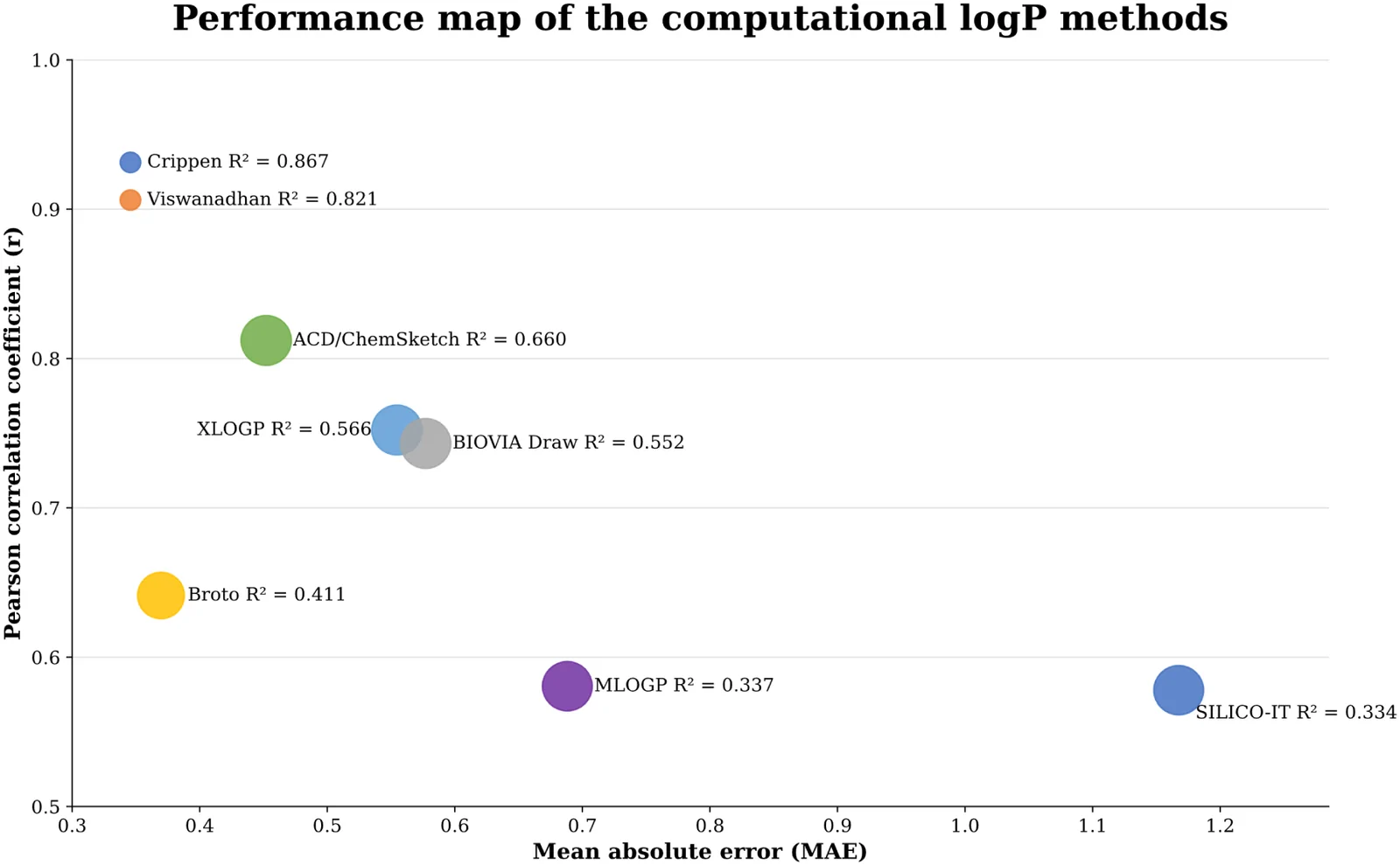
Performance map of the computational log *P* methods. Methods in the upper-left region combine stronger correlation with lower absolute error. Marker size reflects the number of available compound predictions.

The experimental log *P* values reflected the structural features of the investigated compounds. In the rivastigmine analogue series, phenyl-substituted compounds showed the highest lipophilicity, including (**2**) (log *P* = 4.21), (**16**) (log *P* = 4.06), (**11**) (log *P* = 3.87), and (**6**) (log *P* = 3.67). These analogues contain additional aromatic surface area, which increases hydrophobic character and favors partitioning into the organic phase. This observation is consistent with general medicinal chemistry principles, where aromatic and hydrophobic substituents increase lipophilicity and may support membrane permeability, but may also increase nonspecific binding and safety liabilities when overused.^102,107^ Thus, the high log *P* values of (**2**), (**11**), and (**16**) may be favorable for BBB interaction, but these compounds should also be monitored for solubility and nonspecific binding during further profiling.

In contrast, −NO_2_- and −F-substituted analogues without additional phenyl substitution generally showed lower experimental log *P* values. (**14**) and (**18**) displayed the lowest log *P* values in the series, 1.86 and 1.87, respectively, while (**9**) and (**12**) also showed relatively low values of 2.36 and 2.41. The lower lipophilicity of these analogues can be rationalized by the presence of polar nitro groups, which increase polarity and hydrogen-bond accepting capacity and reduce apparent hydrophobic partitioning. This supports the broader concept that lipophilicity must be balanced against polarity; higher polarity may improve aqueous behavior, but excessive polarity can reduce passive membrane permeability, including BBB penetration.^103,104^ Cl-substituted compounds without additional phenyl groups, such as (**8**), (**10**), (**13**), (**15**), and (**22**) occupied an intermediate lipophilicity range, indicating that chlorine contributes to hydrophobicity but less strongly than introduction of a full phenyl ring.

The −NO_2_ group also warrants a balanced safety discussion. Nitroaromatic motifs are recognized structural alerts because enzymatic −NO_2_ reduction can generate nitroso and hydroxylamine intermediates, reactive oxygen species, and, in susceptible structures, covalent deoxyribonucleic acid (DNA) adducts.^78,79^ Nevertheless, the presence of −NO_2_ is not by itself proof of unacceptable toxicity: several clinically used drugs contain nitro groups, and the available evidence is explicitly structure- and metabolism-dependent.^79^ Accordingly, the −NO_2_-containing analogues should not be excluded solely on this basis, but should undergo targeted reactive-metabolite, glutathione-trapping, cytotoxicity, and genotoxicity assessment during further development.

The donepezil-like hybrids also showed moderate experimental log *P* values. Compound (**24**) showed the highest lipophilicity within this subgroup (log *P* = 3.57), followed by (**26**) (log *P* = 3.41), whereas (**25**) was less lipophilic (log *P* = 2.80). This trend is consistent with the structural differences between the analogues: the −Cl-containing hybrids showed higher lipophilicity than the −F/–NO_2_-substituted analogue.

Compared with rivastigmine (log *P* = 2.14), most analogues were more lipophilic, whereas only (**2**) and (**16**) exceeded donepezil (log *P* = 3.93), with (**11**) showing a similar value. Overall, phenyl-substituted analogues were more donepezil-like, while −NO_2_-containing compounds showed lipophilicity closer to rivastigmine.

### 3.7 Aqueous Solubility Assessment

The apparent aqueous solubility of the compounds was determined in artificial gastric fluid (JP1, pH 1.2), simulated intestinal medium (JP2, pH 6.8) and phosphate buffer at pH 7.4 using a DMSO-based chromatographic screening assay. Because the compounds were introduced from DMSO stocks and equilibrated for 1 h, the values should be interpreted as kinetic or apparent solubility rather than equilibrium intrinsic solubility; such assays are suitable for rapid discovery-stage ranking but remain sensitive to residual cosolvent, precipitation kinetics, incubation time, and the solid form generated during the experiment.^46,56,108,109^

The analogues investigated showed a broad solubility range (Table 13). In the JP1 medium, the measurable values of the analogues ranged from 4.8 to 65.9 μg/mL. In JP2, measurable values ranged from 1.3 to 58.5 μg/mL, while (**2**), (**11**), (**16**), and (**21**) were below 1 μg/mL; at pH 7.4, measurable values ranged from 1.1 to 53.3 μg/mL, with compounds (**1**), (**2**), (**6**), (**7**), (**11**), (**16**), and (**21**) below 1 μg/mL. (**26**) was the most soluble analogue in all three media, followed by compounds (**4**), (**8**), (**10**), (**15**), (**24**) and (**25**), whereas compounds (**2**), (**6**), (**11**), (**16**), and (**21**) were the least soluble and may present formulation or exposure liabilities. Poor solubility can compromise dissolution, assay reliability, dose proportionality, and dosage-form development.^110–113^

**Table 13:** Aqueous Solubility of Investigated Compounds in Comparison to Donepezil and Rivastigmine^a^

Compound	JP1 [μg/mL] ± CV [%]	JP2 [μg/mL] ± CV [%]	7.4 [μg/mL] ± CV [%]	*k*′ ± CV [%]	*R* ^^2^^
(**1**)	5.5 ± 2.6	3.9 ± 8.6	<1	4.06 ± 0.1	0.9933
(**2**)	14.6 ± 0.7	<1	<1	4.54 ± 0.1	0.9983
(**3**)	38.4 ± 1.4	18.0 ± 0.6	1.1 ± 3.5	3.88 ± 0.2	0.9979
(**4**)	49.0 ± 5.1	35.5 ± 4.9	19.4 ± 4.8	3.84 ± 0.1	0.9933
(**5**)	19.8 ± 3.9	5.5 ± 6.0	5.2 ± 3.2	4.14 ± 0.1	0.9938
(**6**)	11.2 ± 1.0	1.3 ± 3.3	<1	4.31 ± 0.1	0.9964
(**7**)	17.0 ± 3.8	11.2 ± 5.5	<1	4.06 ± 0.1	0.9961
(**8**)	44.6 ± 2.5	43.0 ± 6.9	16.6 ± 4.2	4.02 ± 0.1	0.9933
(**9**)	36.0 ± 3.1	14.1 ± 4.2	12.0 ± 2.1	3.86 ± 0.1	0.9932
(**10**)	46.9 ± 1.7	35.0 ± 3.8	13.1 ± 1.7	4.09 ± 0.1	0.9955
(**11**)	6.7 ± 4.5	<1	<1	4.37 ± 0.1	0.9980
(**12**)	35.4 ± 5.1	28.2 ± 1.9	18.6 ± 2.1	3.88 ± 0.1	0.9938
(**13**)	27.8 ± 2.3	29.5 ± 8.5	6.4 ± 7.1	4.19 ± 0.1	0.9958
(**14**)	19.4 ± 0.5	14.1 ± 4.1	9.9 ± 1.0	4.04 ± 0.1	0.9954
(**15**)	53.3 ± 5.1	45.3 ± 0.8	29.4 ± 5.2	4.01 ± 0.2	0.9936
(**16**)	4.8 ± 0.7	<1	<1	4.45 ± 0.1	0.9916
(**17**)	25.7 ± 4.8	8.2 ± 5.0	5.0 ± 6.6	4.08 ± 0.2	0.9976
(**18**)	32.4 ± 1.4	15.6 ± 1.3	13.4 ± 1.7	3.68 ± 0.1	0.9973
(**19**)	20.1 ± 0.9	14.1 ± 3.3	9.4 ± 8.7	4.22 ± 0.1	0.9947
(**20**)	12.5 ± 1.5	23.6 ± 4.5	5.6 ± 1.6	4.04 ± 0.2	0.9960
(**21**)	7.4 ± 6.1	<1	<1	4.04 ± 0.1	0.9963
(**22**)	32.9 ± 5.3	28.2 ± 4.2	15.1 ± 1.4	3.87 ± 0.8	0.9984
(**23**)	41.2 ± 6.1	29.5 ± 5.6	23.5 ± 2.2	3.88 ± 0.3	0.9992
(**24**)	50.2 ± 2.7	44.4 ± 1.3	24.8 ± 1.2	2.87 ± 0.4	0.9955
(**25**)	35.7 ± 3.8	33.6 ± 1.7	26.9 ± 1.0	2.70 ± 0.3	0.9945
(**26**)	65.9 ± 4.2	58.5 ± 0.5	53.3 ± 1.1	2.86 ± 0.3	0.9999
donepezil	73.4 ± 1.5	72.9 ± 3.3	63.9 ± 2.7	3.01 ± 0.3	0.9966
rivastigmine	78.8 ± 1.9	82.8 ± 1.8	78.1 ± 0.7	2.64 ± 0.2	0.9941

a JP1 – artificial gastric fluid; JP2 – artificial intestinal fluid media; 7.4 – phosphate buffer based on physiological conditions media; *k*′ – capacity (retention) factor; *R* ^2^ – coefficient of determination of the corresponding external four-point calibration curve.

Across the synthesized series, apparent solubility was higher in JP1 than in JP2 for 24 of the 26 analogues and higher than at pH 7.4 for all analogues. At pH 1.2, basic nitrogen centers, where present, are expected to be more extensively protonated, which increases the charged fraction, aqueous hydration and therefore the apparent solubility. This acid-driven effect is particularly plausible for compounds (**24**)–(**26**), which contain a donepezil-like tertiary amine.

The structural pattern supports this interpretation. The extra phenyl ring in compounds (**2**), (**5**), (**6**), (**11**) and (**16**) increases the hydrophobic surface area, which can strengthen the crystal packing. The JP1 solubility of these compounds shows an improvement, but it still remains among the lowest. In contrast, the Cl-substituted analogues without an additional phenyl ring, compounds (**4**), (**8**), (**10**), (**15**), and (**22**) showed favorable JP1 solubility. NO_2_-containing analogues remained variable because added polarity can be offset by lattice-stabilizing intermolecular interactions and the NO_2_ group is unlikely to be the principal protonation site. Thus, the pH-driven ionization raises JP1 solubility, whereas substitution pattern and solid-state behavior govern the residual differences.

For most compounds, solubility was higher in JP2 than at pH 7.4, particularly for compounds (**3**), (**8**), (**10**), (**13**) and (**20**). This may reflect a larger protonated fraction of compounds containing basic centers at the lower pH, although buffer identity and ionic strength can also influence the measured concentration. Apparent solubility of ionizable molecules is therefore governed by pH, p*K*_a_, intrinsic solubility, and solid-state behavior rather than by lipophilicity alone.^100,114–116^

The structural trends were broadly consistent with the experimental log *P* data. Phenyl-substituted compounds (**2**), (**6**), (**11**), and (**16**), with log *P* ranging from 3.67 to 4.21, showed very low solubility, plausibly because the additional aromatic ring increases hydrophobic surface area and may strengthen crystal packing. NO_2_-containing analogues showed variable solubility because the nitro group increases polarity and hydrogen-bond-accepting capacity, which may lower log *P* and favor aqueous hydration. However, it can also participate in directional intermolecular interactions that stabilize the crystal lattice and limit dissolution, making its overall effect strongly dependent on molecular structure and solid-state packing.^117,118^ Cl-substituted analogues without an additional phenyl ring, including 4, 8, 10, 15, and 22, generally showed intermediate to favorable solubility. These observations are consistent with the principle that aqueous solubility reflects both hydration and crystal-lattice energy and cannot be inferred from log *P* alone.^76,100,115,119,120^

Compounds (**24**)–(**26**), which contain a donepezil-like tertiary amine, were considerably more soluble than most members of the first series. The JP1 solubility of these compounds was within the range of 35.7 to 65.9 μg/mL. Their JP2 solubilities ranged from 33.6 to 58.5 μg/mL and their pH 7.4 values from 24.8 to 53.3 μg/mL. Protonation of the tertiary amine increases hydration and explains why compounds (**24**) and (**26**) retained favorable solubility despite experimental log *P* values of 3.57 and 3.41, respectively. Donepezil and rivastigmine showed the highest values overall, reaching 73.4/72.9/63.9 and 78.8/82.8/78.1 μg/mL in JP1/JP2/pH 7.4 media respectively; the higher solubility of rivastigmine is consistent with its lower experimental log *P* value of 2.14 compared with 3.93 for donepezil. For ionizable compounds, the observed aqueous concentration therefore reflects the combined effects of neutral-form lipophilicity and the fraction present in the charged form.^104,114^

A critical point is that the synthesized compounds were tested as nonsalt free forms. For ionizable compounds, conversion to a pharmaceutically acceptable salt is a widely used strategy for increasing dissolution rate and apparent aqueous solubility by providing an ionized solid form and modifying the local pH during dissolution; accordingly, the present values should be regarded as conservative baseline measurements rather than the maximum solubility achievable during pharmaceutical development.^121–124^

Salt formation, however, does not guarantee a sustained increase in equilibrium solubility. The magnitude and persistence of the benefit depend on p*K*_a_, counterion, salt solubility product, medium pH, ionic strength, common-ion effects, polymorphism, and possible conversion back to the less soluble free form; salt screening and solid-state characterization are therefore required before definitive developability conclusions can be made.^121,125^

Overall, (**26**) showed the most favorable solubility profile among the synthesized analogues, whereas compounds (**2**), (**6**), (**11**), (**16**) and (**21**) should be prioritized for salt selection, formulation optimization, and equilibrium-solubility studies.

### 3.8 Integrated ADME and Biological Lead Prioritization

#### 3.8.1 ADME-Only Prioritization

To provide a transparent basis for lead selection across all 26 analogues, an exploratory weighted ADME balance score (0–100) was constructed (Table 14) from the experimental and model-based end points reported in this study with the use of IBM SPSS Statistics 30 for Windows 11 (IBM Corp, Armonk, USA). The score is intended only as a within-series prioritization aid and should not be interpreted as a validated pharmacokinetic or clinical prediction model.

**Table 14:** ADME-Only Summary and Exploratory Lead-Prioritization Score for the 26 Synthesized Analogues^a^

compound	human/rat plasma 24 h remaining (%)	HSA/AGP PPB (%)	CHI_IAM_/*P*_m_/ %HIA	*V*_d_/log *V*_du_/BTB	experimental log *P*	solubility JP1/JP2/pH 7.4 (μg/mL)	ADME score/rank/tier^b^
(**15**)	80.1/78.3	96.6/79.7	36.1/12.41/96	0.73/1.46/94.4	2.82	53.3/45.3/29.4	96.7/1/A
(**8**)	85.6/90.6	96.2/85.9	35.9/11.31/95	0.78/1.41/94.3	2.83	44.6/43.0/16.6	94.4/2/A
(**26**)	92.3/89.1	93.5/79.9	48.5/6.57/89	6.95/2.04/95.6	3.41	65.9/58.5/53.3	93.0/3/A
(**4**)	90.7/88.4	95.6/84.3	34.1/7.70/94	0.71/1.30/93.7	1.99	49.0/35.5/19.4	92.3/4/A
(**10**)	78.7/72.8	95.9/86.4	36.1/11.15/95	0.84/1.40/94.2	2.94	46.9/35.0/13.1	92.1/5/A
(**24**)	91.4/72.2	95.7/79.5	34.2/6.73/89	0.72/1.30/93.7	3.57	50.2/44.4/24.8	90.2/6/A
(**13**)	88.5/86.8	97.2/81.2	37.8/11.70/97	0.78/1.60/94.9	3.23	27.8/29.5/6.4	88.4/7/B
(**22**)	83.4/57.0	94.4/94.8	34.5/8.36/94	0.87/1.24/93.4	3.00	32.9/28.2/15.1	85.6/8/B
(**5**)	97.2/78.6	97.0/85.7	38.1/18.65/97	0.86/1.59/94.9	3.25	19.8/5.5/5.2	83.3/9/B
(**23**)	67.9/74.9	96.7/79.1	35.2/6.95/88	0.66/1.42/94.3	2.68	41.2/29.5/23.5	82.7/10/B
(**25**)	90.4/89.6	96.1/78.1	34.9/3.15/81	0.72/1.36/94.0	2.80	35.7/33.6/26.9	81.2/11/B
(**17**)	88.2/76.3	96.5/89.3	36.1/8.79/91	0.76/1.45/94.4	3.09	25.7/8.2/5.0	81.1/12/B
(**19**)	92.0/88.7	96.4/79.7	43.4/5.25/89	1.95/1.84/95.5	3.48	20.1/14.1/9.4	81.0/13/B
(**14**)	84.5/89.9	95.6/92.3	34.5/6.57/90	0.74/1.31/93.8	1.86	19.4/14.1/9.9	80.8/14/B
(**12**)	80.6/64.6	94.2/92.0	32.7/3.93/86	0.74/1.16/92.8	2.41	35.4/28.2/18.6	80.0/15/C
(**9**)	92.6/65.9	94.8/89.1	32.9/4.29/87	0.71/1.19/93.1	2.36	36.0/14.1/12.0	78.6/16/C
(**7**)	89.4/74.9	96.7/88.2	36.2/9.71/100	0.73/1.47/94.5	2.85	17.0/11.2/<1	78.1/17/C
(**3**)	87.6/82.4	95.8/87.3	34.7/6.19/88	0.74/1.33/93.9	2.30	38.4/18.0/1.1	76.0/18/C
(**18**)	89.0/64.4	92.9/87.3	30.9/2.81/82	0.72/1.03/91.7	1.87	32.4/15.6/13.4	73.5/19/C
(**1**)	89.5/75.3	96.8/79.8	36.7/10.66/91	0.76/1.51/94.6	3.09	5.5/3.9/<1	72.8/20/C
(**6**)	95.0/92.3	97.6/86.9	39.7/18.66/97	0.89/1.74/95.3	3.67	11.2/1.3/<1	72.6/21/C
(**11**)	95.5/69.5	97.6/86.3	40.1/12.31/96	0.93/1.77/95.4	3.87	6.7/<1/<1	69.9/22/D
(**20**)	68.8/38.7	95.4/72.8	38.1/5.63/89	1.15/1.46/94.4	2.64	12.5/23.6/5.6	69.8/23/D
(**21**)	83.7/40.8	97.6/82.4	38.2/7.61/90	0.74/1.67/95.1	2.65	7.4/<1/<1	62.1/24/D
(**2**)	85.6/70.8	97.8/87.4	41.6/3.14/96	1.09/1.88/95.6	4.21	14.6/<1/<1	60.5/25/D
(**16**)	66.4/35.6	97.5/86.7	41.1/7.26/96	1.11/1.81/95.4	4.06	4.8/<1/<1	55.1/26/D

a Values are shown as human/rat parent compound remaining at 24 h, HSA/AGP binding, CHIIAM/*P*_m_/%HIA, *V*_d_/log *V*_du_/BTB, experimental log *P*, and JP1/JP2/pH 7.4 solubility. All analogues were classified as CNS-positive by the IAM model: Tier A: Score ≥ 90; Tier B: 80–89.9; Tier C: 70–79.9; Tier D: <70.

b Scoring note: human plasma stability reached full credit at ≥80% parent remaining at 24 h and rat plasma stability at ≥75%, with no additional reward for very high values; HSA ≤ 95% and AGP ≤ 85% incurred no PPB penalty, with progressively greater penalties above these levels; *P*_m_ and %HIA were capped once high within-series permeability/absorption was reached; *V*_d_ values in the broadly moderate range were favored without treating larger distribution as inherently superior; log *P* values of 2–4 received full credit; solubility desirability increased on a log-like scale, with the greatest weight assigned to pH 7.4. These thresholds are pragmatic discovery-stage ranking rules for this chemical series rather than validated clinical cutoffs.

To reduce double counting of the correlated descriptors, the score was organized into seven domains in total, namely: plasma stability (15%; human 10% and rat 5%), PPB balance (10%; HSA 5%, AGP 5%), IAM-based BBB permeability (*P*_m_, 15%), predicted intestinal absorption (%HIA 10%), biomimetic distribution (10%; *V*_d_ 4%, log *V*_du_ 3%, BTB 3%), experimental log *P* (10%) and solubility (30%; JP1 6%, JP2 9%, pH 7.4 15%). Each parameter was converted to a 0–100 desirability score based on predefined ranges, with values between the limits assigned proportionally. The individual scores were then combined using the selected weights to obtain the overall ADME score (Figure 3).

**Figure 3:**
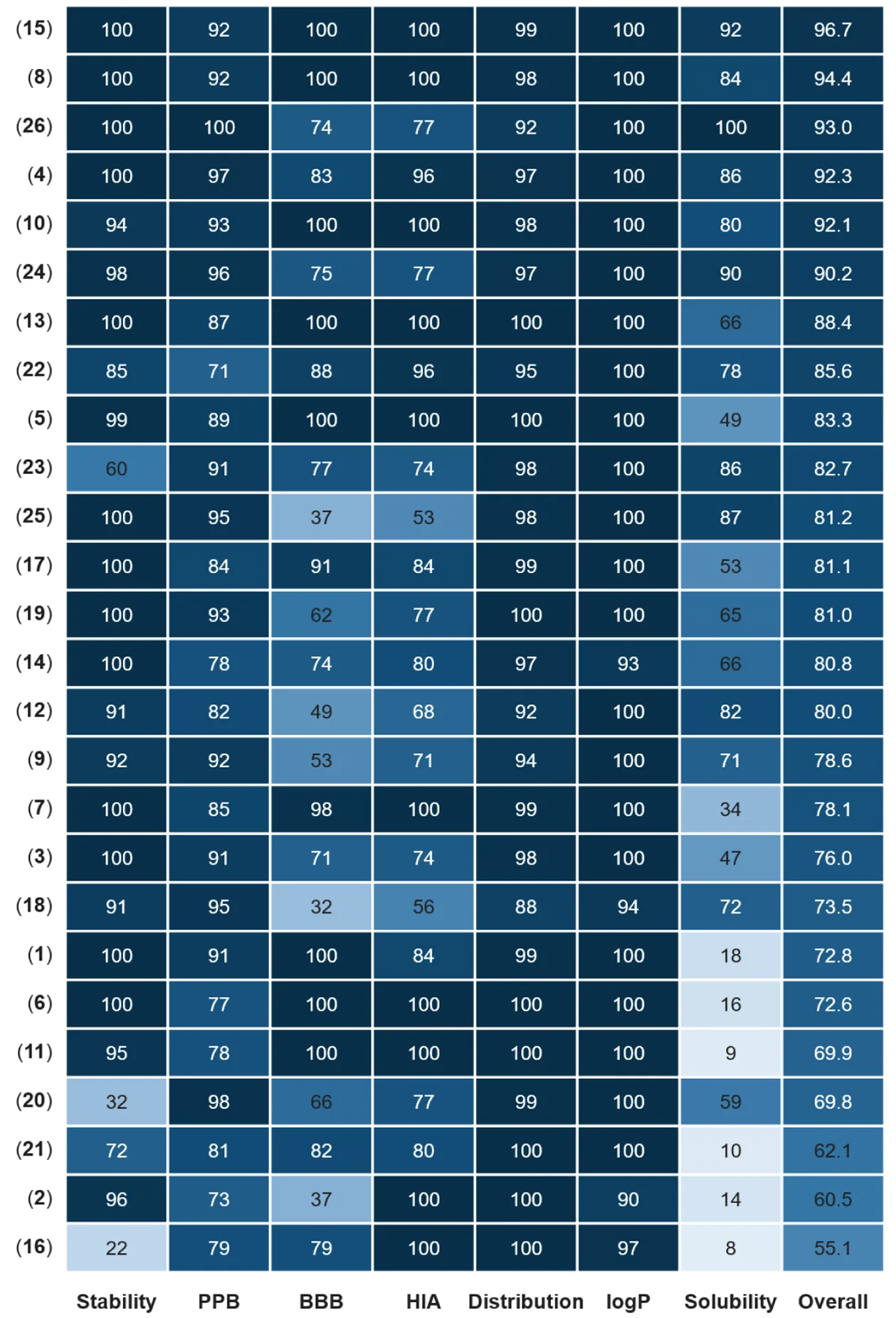
Exploratory ADME desirability heatmap for the 26 analogues, ordered by the ADME-only score. Each domain is normalized from 0 (least favorable) to 100 (most favorable). The PPB and plasma-stability domains intentionally do not reward extreme values.

Two deliberate safeguards were introduced to avoid biologically misleading “higher-is-better” scoring. First, plasma stability was scored from the directly observed percentage of parent compound remaining at 24 h rather than from the extrapolated apparent half-life. Full credit was reached once adequate stability was achieved and values above this range did not receive additional benefit. This reflects the fact that an *in vitro* plasma-stability assay measures susceptibility to degradation in plasma and does not by itself determine hepatic clearance, renal clearance, systemic exposure, or clinical elimination half-life. High plasma stability can be advantageous by limiting loss of the parent compound before it reaches its target, but if it occurs together with low overall systemic clearance it may also contribute to prolonged exposure and accumulation; the present plasma-only assay cannot determine that outcome.^23,51^ Second, PPB was treated as a balance parameter rather than as a positive monotonic property: HSA binding above 95% and AGP binding above 85% were progressively penalized, because very high binding can reduce the unbound fraction and complicate exposure interpretation, while PPB alone does not predict *in vivo* efficacy. Likewise, high PPB can buffer total circulating concentrations and influence clearance, but it can also reduce the immediately unbound fraction and increase sensitivity to protein concentration or displacement; neither maximal nor minimal PPB is therefore universally desirable.^24,26,58,59^

The predicted *V*_d_, log *V*_du_, BTB, *P*_m_, and HIA were used as comparative ranking descriptors rather than direct pharmacokinetic measurements. The HSA/IAM-based *V*_d_ model was originally developed to estimate human distribution from protein and phospholipid binding, while the IAM-based BBB and intestinal absorption models primarily reflect passive membrane interaction and do not comprehensively capture active uptake, efflux transporters, metabolism, or other *in vivo* determinants of exposure.^36,40^

Very large *V*_d_ values were therefore not automatically rewarded, because increasing tissue distribution is not intrinsically beneficial and may trade off against other pharmacokinetic properties.^28^ Experimental log *P* values in the intermediate range were favored, as excessive lipophilicity can negatively affect solubility, nonspecific binding, clearance, and overall compound quality even when membrane partitioning is improved.^76^

Using this framework, the six analogues that formed the highest ADME tier were as follows: (**15**) (96.7; rank 1), (**8**) (94.4; rank 2), (**26**) (93.0; rank 3), (**4**) (92.3; rank 4), (**10**) (92.1; rank 5), and (**24**) (90.2; rank 6). (**15**) and (**8**) were the most balanced across the experimental and model-based domains, combining acceptable cross-species plasma stability with nonextreme PPB, high *P*_m_, high %HIA, moderate predicted distribution, intermediate log *P* and comparatively favorable solubility. (**26**) was distinguished by the best solubility profile of the series (65.9/58.5/53.3 μg/mL in JP1/JP2/pH 7.4), high human and rat plasma stability (92.3%/89.1% remaining at 24 h), the lowest HSA binding among the donepezil-like analogues (93.5%), high predicted BTB (95.6%) and an intermediate experimental log *P* of 3.41. Its main model-based trade-off was a larger predicted *V*_d_ (6.95 L/kg) together with only intermediate *P*_m_ (6.57) and %HIA (89%). Compound (**24**) similarly combined favorable solubility (50.2/44.4/24.8 μg/mL), high human-plasma stability (91.4% remaining), nonextreme HSA/AGP binding (95.7%/79.5%) and a balanced *V*_d_/log *V*_du_/BTB profile, although *P*_m_ (6.73) and HIA (89%) were lower than those of the highest-permeability analogues.

Several lower-ranked compounds were limited by a single dominant developability liability rather than a globally unfavorable profile. Compounds (**2**), (**6**), (**11**), (**16**) and (**21**) showed particularly poor JP2 and/or pH 7.4 solubility, while compounds with HSA binding above 97% or AGP binding above 90% were not promoted solely on the basis of their high bound fraction. (**16**) additionally showed the least favorable plasma-stability profile, with only 66.4% and 35.6% parent compound remaining after 24 h in human and rat plasma, respectively.

For ionizable analogues, salt formation represents a rational formulation strategy that can improve dissolution rate and apparent aqueous solubility, but the benefit depends on p*K*_a_, counterion, salt solubility product, medium pH, and solid-state behavior and may be lost if the salt converts back to the less soluble free form.^110,121,122,126^

The donepezil-like tertiary amines (**24**)–(**26**) are particularly suitable for formal salt screening, whereas poorly soluble analogues lacking a sufficiently ionizable center may require alternative formulation approaches or structural optimization.^114^

#### 3.8.2 Integration with Previously Reported Biological Activity

The biological activity values summarized in Tables 1 and 2 were reported in the previously published characterization of the rivastigmine-derived and donepezil-like hybrid series and thus were not remeasured in the present ADME study.^16,17^ Accordingly, the following analysis (Table 15) should be interpreted as a cross-study integration of existing pharmacological evidence with the current ADME data, rather than as new biological validation. This distinction is of importance, as a compound with an excellent ADME profile is not necessarily the most pharmacologically active lead, while the most potent enzyme inhibitor may still be limited by solubility, protein binding, permeability, or metabolic liabilities.

**Table 15:** Integrated Biological-ADME Prioritization of Representative Candidates^a^

compound	ADME score/rank	biological activity	ADME strengths	liabilities
(**24**)	90.2/6	*h*AChE 0.0008	Good solubility; 91.4% human 24 h remaining; HSA/AGP 95.7/79.5%; *P*_m_ 6.73; HIA 89%; balanced *V*_d_/BTB	Rat stability lower than human; BBB/HIA are model-based and intermediate rather than maximal
		*eq*BChE 1.41		
(**26**)	93.0/3	*h*AChE 1.68	Best solubility; 92.3/89.1% human/rat 24 h remaining; HSA/AGP 93.5/79.9%; BTB 95.6%	Predicted *V*_d_ 6.95 L/kg; *P*_m_ 6.57 and HIA 89%
		eqBChE 5.61		
(**3**)	76.0/18	*h*AChE 0.036	Strong multitarget potency; 87.6/82.4% human/rat 24 h remaining; log *P* 2.30	pH 7.4 solubility 1.1 μg/mL; *P*_m_ 6.19; HIA 88%; long human *t*_1/2_ – estimate uncertain
		eqBChE 2.76		
		hFAAH 3.56		
(**18**)	73.5/19	*h*AChE 0.014	Very strong cholinesterase activity; measurable solubility across all media; log *P* 1.87	*P*_m_ 2.81; HIA 82%; rat 24 h remaining 64.4%; BTB 91.7%
		*eq*BChE 1.68		
		*h*FAAH 8.17		
(**15**)	96.7/1	*h*AChE 4.44	Highest ADME score; *P*_m_ 12.41; HIA 96%; good solubility; balanced distribution	Biological potency more moderate than primary leads
		*eq*BChE 57% at 10 μM		
		*h*FAAH 21.5		
(**25**)	81.2/11	*h*AChE 0.0028	Very high AChE potency; 90.4/89.6% human/rat 24 h remaining; good solubility	*P*_m_ 3.15 and HIA 81% are among the less favorable permeability values in the series
		*eq*BChE 5.01		

a Biological activity values were reported previously; half-maximal inhibitory concentration (IC_50_) values are in μM unless otherwise specified; the integrated priority is a qualitative synthesis of biological potency and the ADME-only score, not a separately validated numerical model.

(**24**) provides the clearest overall potency–developability compromise. It showed the strongest reported *h*AChE inhibition in the present compound set (IC_50_ = 0.0008 ± 0.0002 μM) together with *eq*BChE inhibition of 1.41 ± 0.27 μM, while retaining a Tier-A ADME score of 90.2 (rank 6), favorable solubility, high human-plasma stability, nonextreme PPB, CNS-positive IAM classification and predicted %HIA of 89%. On this basis, (**24**) is proposed as the primary potency–developability lead for confirmatory preclinical studies.

Compound (**26**) is the strongest developability-oriented colead. Its previously reported *h*AChE and *eq*BChE IC_50_ values were 1.68 ± 0.14 and 5.61 ± 0.23 μM, respectively, and its ADME score was 93.0 (rank 3). It also had the best solubility in all three media, high cross-species plasma stability, the lowest HSA binding within the donepezil-like subset, high predicted BTB and an intermediate log *P*. Its main uncertainty is that the predicted *V*_d_ is substantially larger than for most analogues, emphasizing the need to measure actual plasma and tissue exposure rather than assuming that greater distribution is automatically favorable.

Within the rivastigmine-derived series, (**3**) and (**18**) remain important potency-driven candidates despite lower ADME ranks. (**3**) showed *h*AChE, *eq*BChE, and *h*FAAH IC_50_ values of 0.036, 2.76, and 3.56 μM, respectively, but its pH 7.4 solubility was only 1.1 μg/mL. (**18**) showed still stronger *h*AChE inhibition (0.014 μM) and *eq*BChE inhibition (1.68 μM), but lower *P*_m_ (2.81), lower %HIA (82%) and weaker rat-plasma stability (64.4% remaining at 24 h). These two compounds therefore represent pharmacologically attractive secondary leads for which permeability, formulation, and species-dependent stability should be resolved before advancement.

(**15**) illustrates the complementary limitation of ADME-only ranking—it achieved the highest ADME score (96.7) because of its strong *P*_m_ (12.41), %HIA (96%), balanced predicted distribution, intermediate log *P* (2.82) and favorable solubility, yet its previously reported biological activity was more moderate (*h*AChE IC_50_ = 4.44 μM; *eq*BChE inhibitio*n* = 57% at 10 μM; *h*FAAH IC_50_ = 21.5 μM). It should therefore be considered a high-quality ADME benchmark or secondary lead rather than the primary pharmacological candidate. (**25**) represents the opposite trade-off, with very high *h*AChE potency (0.0028 μM), good stability and solubility, but lower *P*_m_ (3.15) and %HIA (81%), whereas (**11**) has broad low-micromolar BChE/FAAH/MAO-B activity but severe JP2 and pH 7.4 solubility limitations (<1 μg/mL) and very high HSA binding (97.6%).

## 4 Conclusion

The present study establishes a comparative ADME profile for 26 donepezil- and rivastigmine-derived hybrids and demonstrates that developability cannot be inferred from biological potency alone. The plasma-stability analysis showed generally limited parent-compound loss over 24 h in human and rat plasma. As many apparent half-lives extended beyond the experimental window and had broad or unbounded confidence intervals, they should be interpreted as comparative *in vitro* stability descriptors rather than predictors of clinical elimination half-life, systemic exposure, or dosing frequency. Species-dependent differences further emphasize the need for cautious extrapolation between rat and human plasma.

Plasma protein binding was high for most analogues, but this was treated as a balance parameter rather than an intrinsic advantage, since very high binding can markedly reduce the immediately unbound fraction and complicate exposure interpretation. The HSA- and IAM-derived distribution parameters therefore provide useful comparative information within the series, but they should not be considered direct measurements of *in vivo* pharmacokinetics.

IAM-based BBB and intestinal absorption models supported favorable passive-permeability profiles across the series, with all analogues classified as CNS-positive and predicted to show high intestinal absorption. Experimental log *P* values were generally compatible with membrane partitioning, whereas solubility provided stronger differentiation between candidates. (**26**) showed the most favorable solubility profile, while several analogues displayed formulation liabilities that warrant salt screening, where chemically appropriate, or alternative formulation and structural optimization.

The integrated analysis in Section 3.8 further showed why ADME-only ranking should not determine lead selection in isolation. The exploratory desirability score placed compounds (**15**), (**8**), (**26**), (**4**), (**10**) and (**24**) in the highest ADME tier. When these data were evaluated alongside the previously reported biological activities, (**24**) provided the clearest potency-developability compromise and was prioritized as the principal lead, while (**26**) emerged as the strongest developability-oriented colead. Compounds (**3**) and (**18**) remain relevant potency-driven secondary candidates, whereas (**15**) is better regarded as an ADME benchmark or secondary lead due to its more moderate biological activity.

Future work should prioritize compounds (**24**) and (**26**) for orthogonal experimental validation, including thermodynamic solubility and salt-form screening, direct determination of unbound fractions, metabolic profiling in microsomal or hepatocyte systems, transporter and efflux assessment, brain-exposure studies and *in vivo* pharmacokinetic and efficacy evaluation. The desirability score is intended as a transparent within-series prioritization tool rather than a validated pharmacokinetic model. Overall, integrating biomimetic ADME profiling with existing pharmacological evidence provides a more balanced basis for selecting candidates for subsequent preclinical development.

## Supplementary Material



## Notes

Chromatographic purity method and chromatograms for compounds (**1**)–(**26**) and blank run (Figures S1–S27); human and rat plasma stability data for compounds (**1**)–(**26**) (Tables S1–S52); statistical comparison of computational plasma protein-binding predictions with experimental biomimetic HSA and AGP binding estimates (Table S53); statistical comparison of experimentally determined HPLC log *P* values with *in silico* predictions (Table S54) (PDF)
