# Supplementary material for: Rapid HPLC-Based ADME Profiling of Novel Donepezil and Rivastigmine Analogues for Potential Application in Alzheimer’s Disease
Source: ACS Omega. 2026 Oct 6;11(41):63529–53. doi: 10.1021/acsomega.6c07555 (PMC13688497; doi:10.1021/acsomega.6c07555)
Supplement: Supplementary file 1 [file ao6c07555_si_001.pdf]

Supporting Information

Rapid HPLC-Based ADME Profiling of Novel Donepezil and Rivastigmine Analogues for Potential Application in Alzheimer's Disease

Stefan Simić[1,2,#], Rosalba Leuci[3], Marianne Pirker[1], Predrag Kalaba[4], Judith Wackerlig-Damle[1], Luca Piemontese[3], Thierry Langer[1,#]

[1]Department of Pharmaceutical Sciences, University of Vienna, Josef-Holaubek-Platz 2, 1090 Vienna, Austria

[2]Vienna Doctoral School of Pharmaceutical, Nutritional and Sport Sciences, University of Vienna, Josef-Holaubek-Platz 2, 1090 Vienna, Austria

[3]Department of Pharmacy—Pharmaceutical Sciences, University of Bari Aldo Moro, via E. Orabona 4, 70126 Bari, Italy

[4]The Florey Institute of Neuroscience and Mental Health, 30 Royal Parade, Parkville, VIC 3052, Australia

#Correspondance to:

Thierry Langer

Department of Pharmaceutical Sciences, Faculty of Life Sciences, University of Vienna, Josef-Holaubek-Platz 2, Vienna, Austria

E-Mail: thierry.langer@univie.ac.at

and

Stefan Simić

Department of Pharmaceutical Sciences, Faculty of Life Sciences, University of Vienna, Josef-Holaubek-Platz 2, Vienna, Austria

E-Mail: stefan.simic@univie.ac.at

The chromatographic purity of the investigated compounds was determined using a Shimadzu Prominence LC-20AD XR liquid chromatograph (Shimadzu Corporation, Tokyo, Japan) equipped with an Acclaim™ 120 C_{18} column (5 µm, 4.6 × 100 mm; Thermo Fisher Scientific, Dreieich, Germany), coupled with a suitable pre-column. The mobile phase consisted of water containing 0.1% formic acid (solvent A) and acetonitrile containing 0.1% formic acid (solvent B). The following gradient program was applied: 0 – 1 min, 20% B; 1 – 7.5 min, linear increase from 20% to 95% B; and 7.5–10 min, return to 20% B for equilibration. The flow rate was set to 1.0 mL/min, the column temperature was maintained at 25 °C and detection was performed at 254 nm. Samples were prepared by diluting the stock solutions with water at a ratio of 1:4 (v/v), resulting in a final concentration of 250 µg/mL. The injection volume was 10 µL.

Figure S1. Chromatogram of (1); retention time at 7.591 minutes; 99.7% purity.

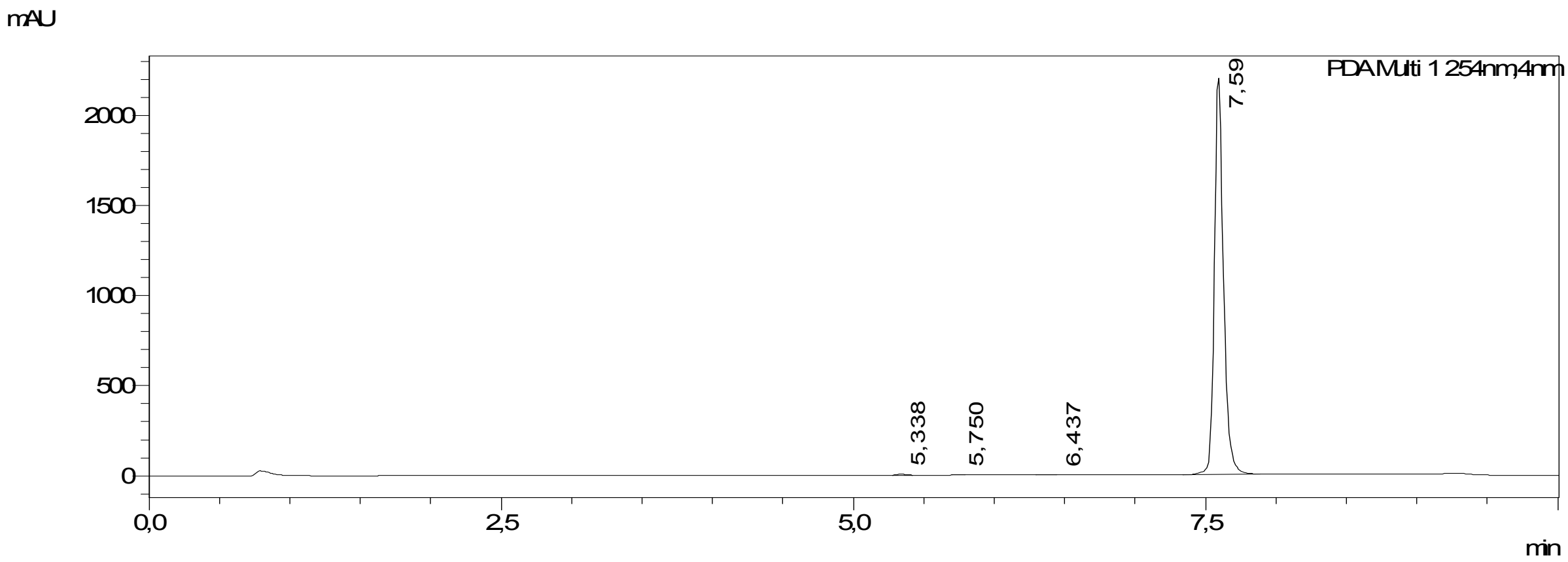

Figure S2. Chromatogram of (**2**); retention time at 7.811 minutes; 99.6% purity.

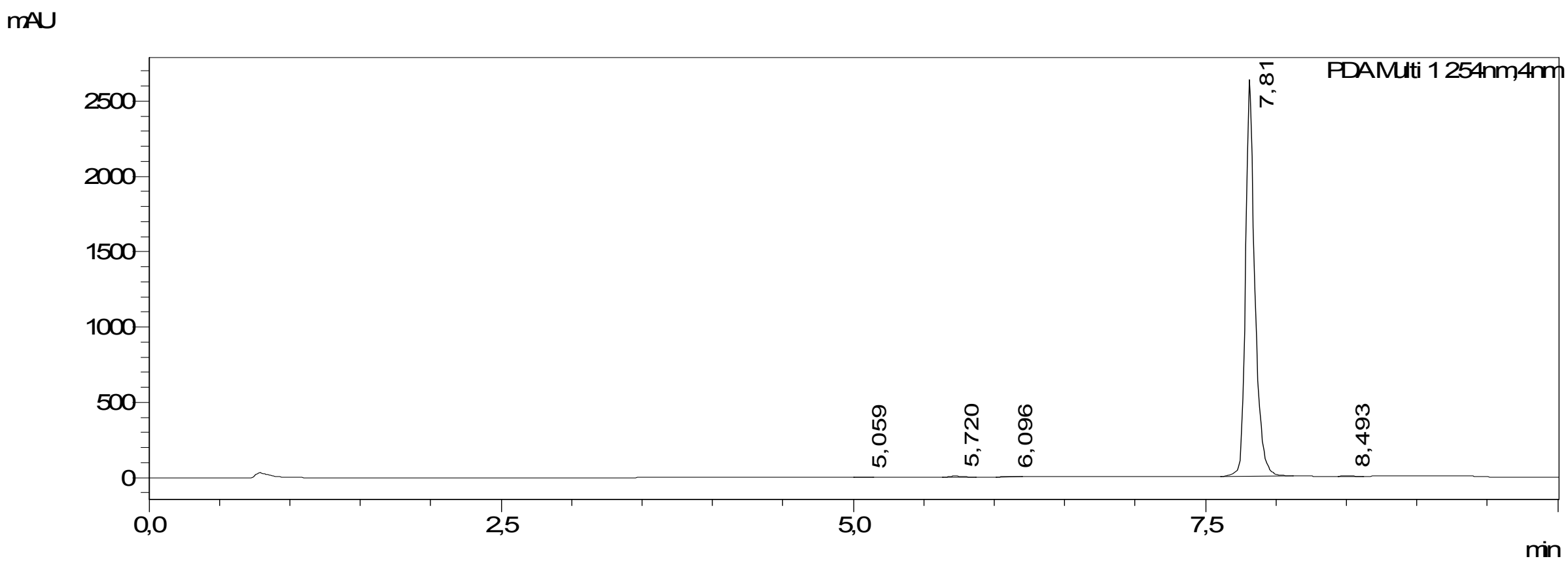

Figure S3. Chromatogram of (**3**); retention time at 6.528 minutes; 96.4% purity.

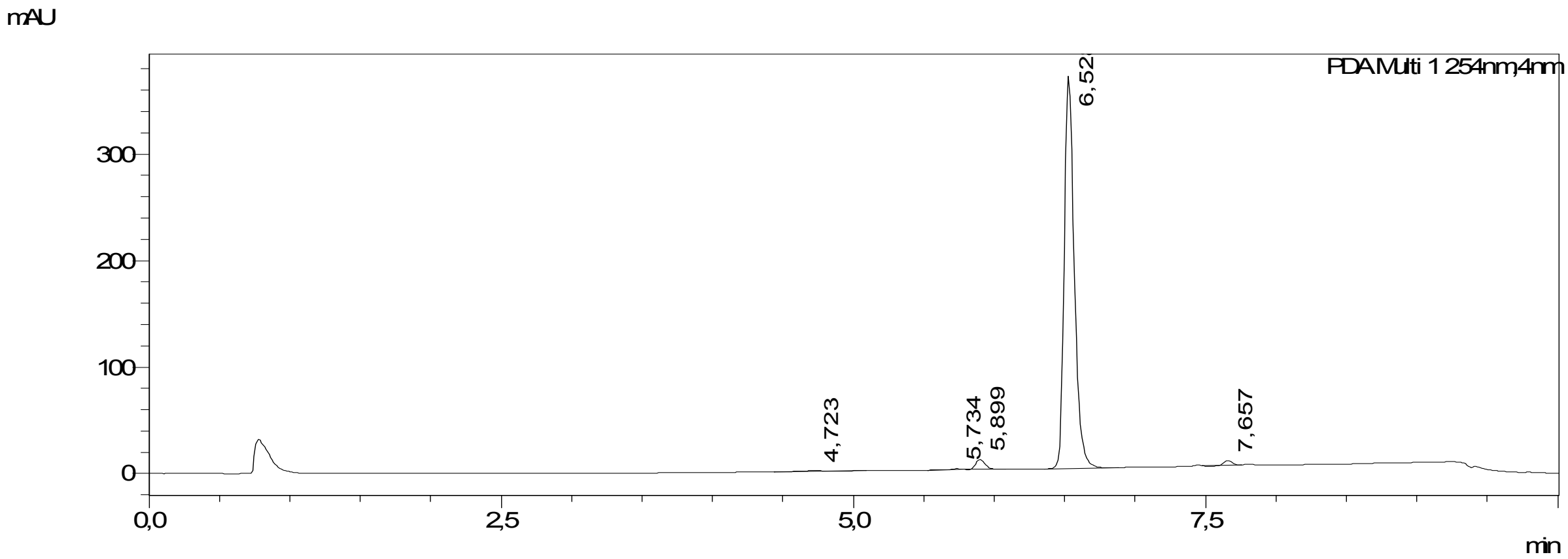

Figure S4. Chromatogram of (**4**); retention time at 6.434 minutes; 95.8% purity.

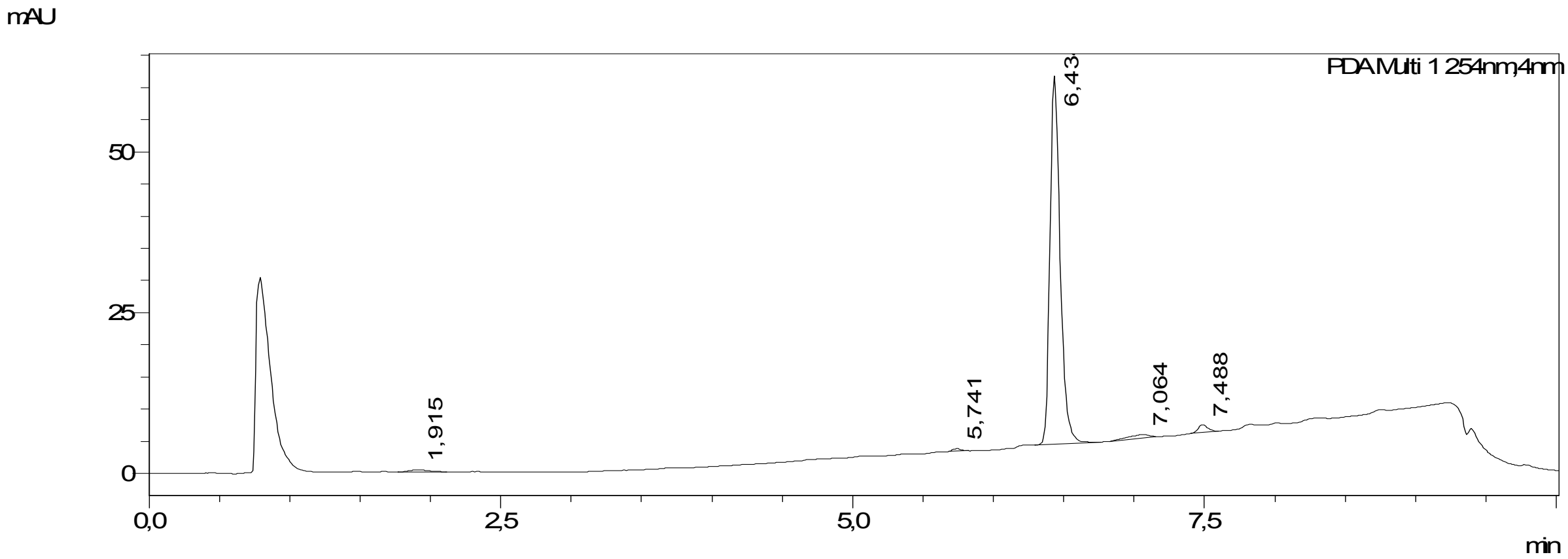

Figure S5. Chromatogram of (**5**); retention time at 7.065 minutes; 99.9% purity.

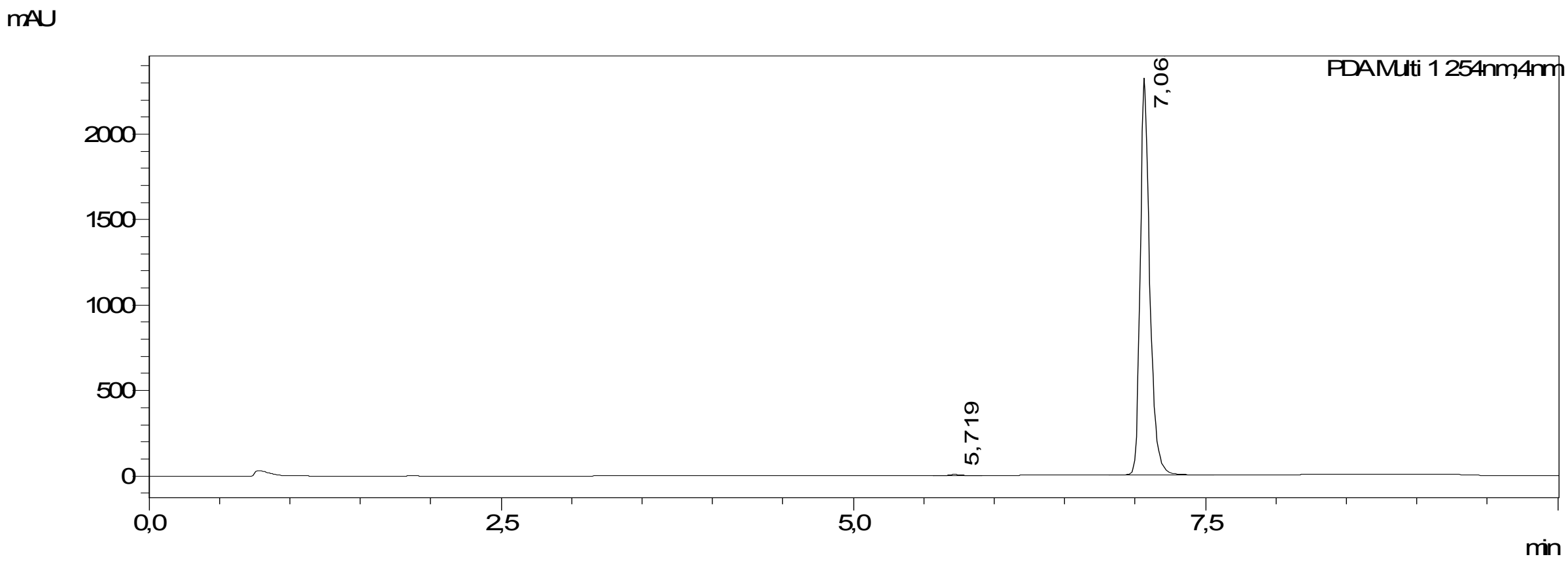

Figure S6. Chromatogram of (**6**); retention time at 7.361 minutes; 99.9% purity.

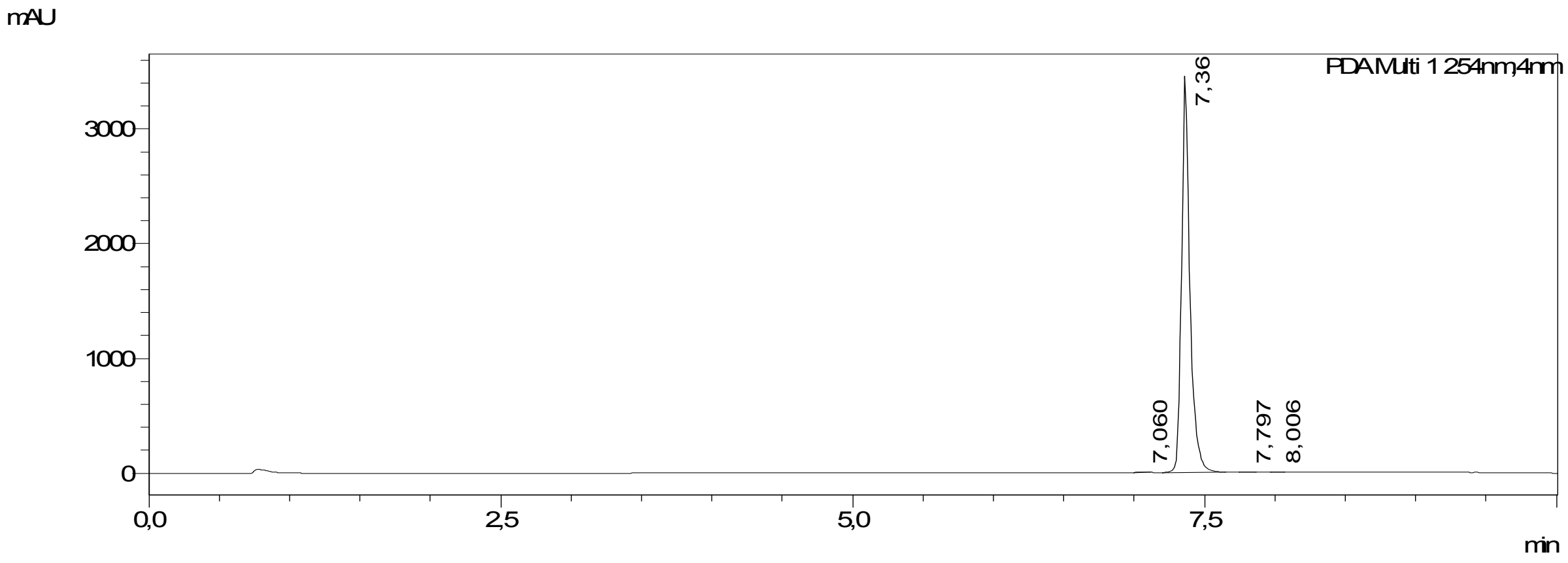

Figure S7. Chromatogram of (**7**); retention time at 6.401 minutes; 95.1% purity.

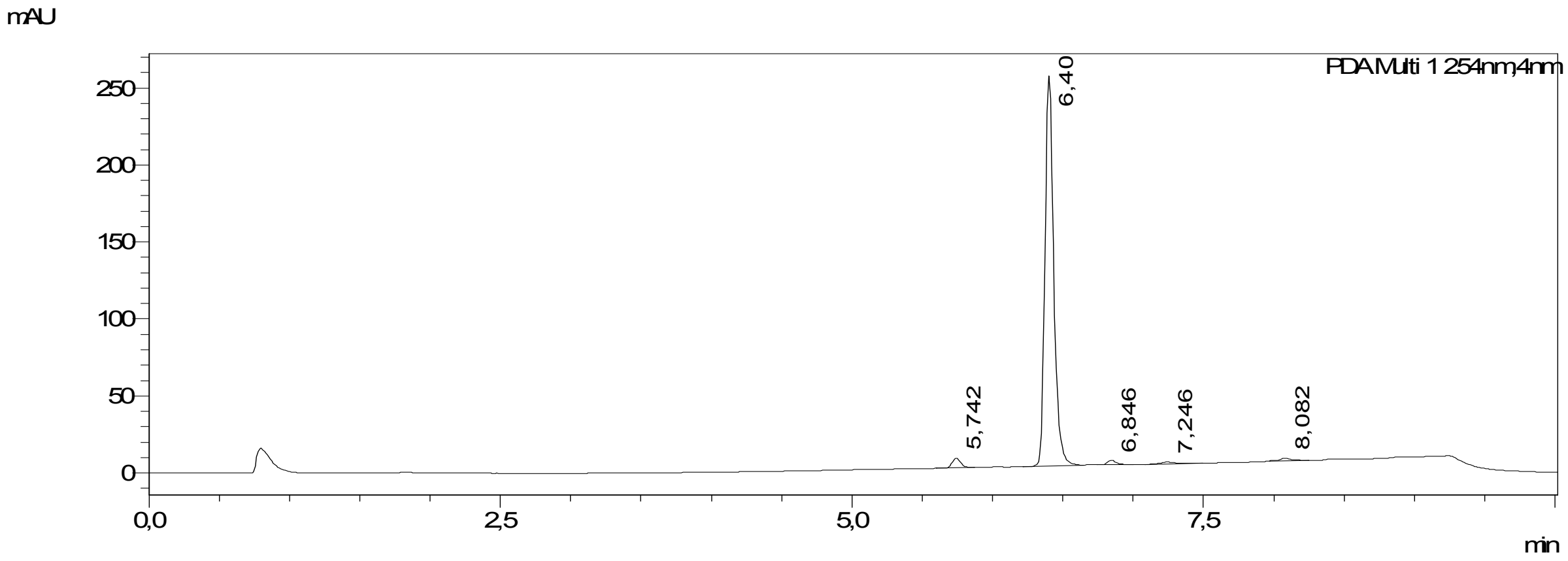

Figure S8. Chromatogram of (**8**); retention time at 5.792 minutes; 96.5% purity.

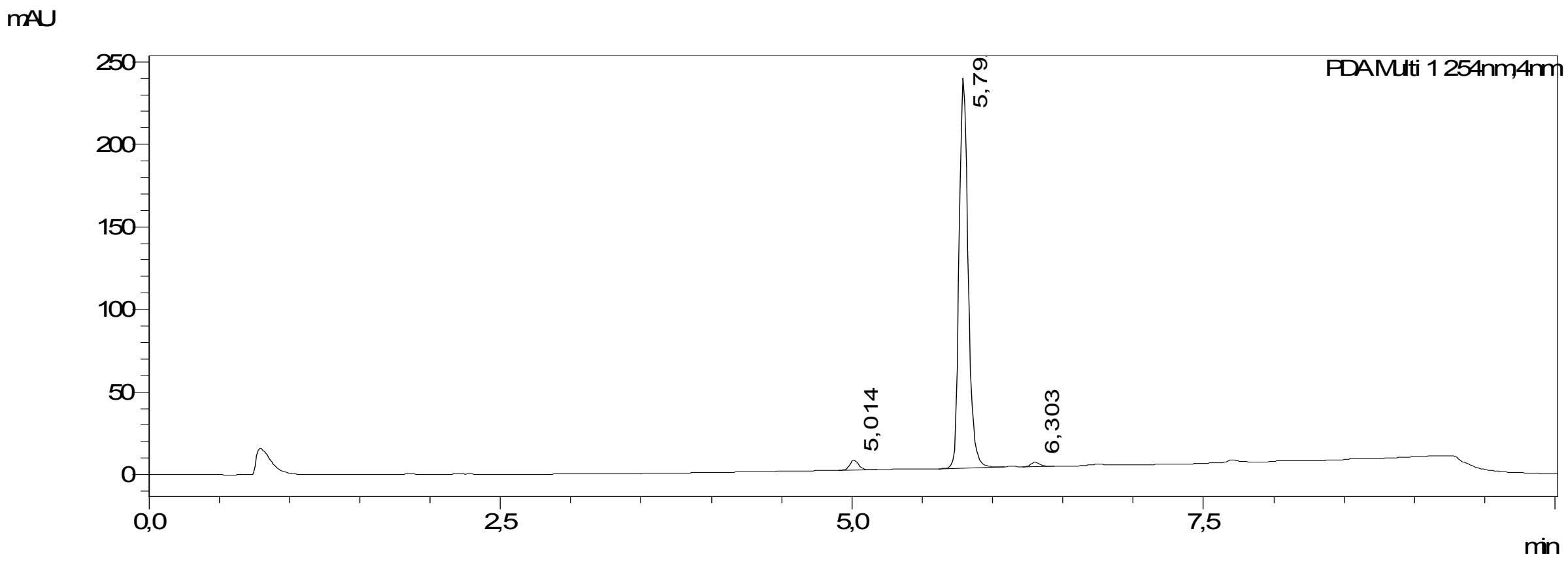

Figure S9. Chromatogram of (**9**); retention time at 6.499 minutes; 95.3% purity.

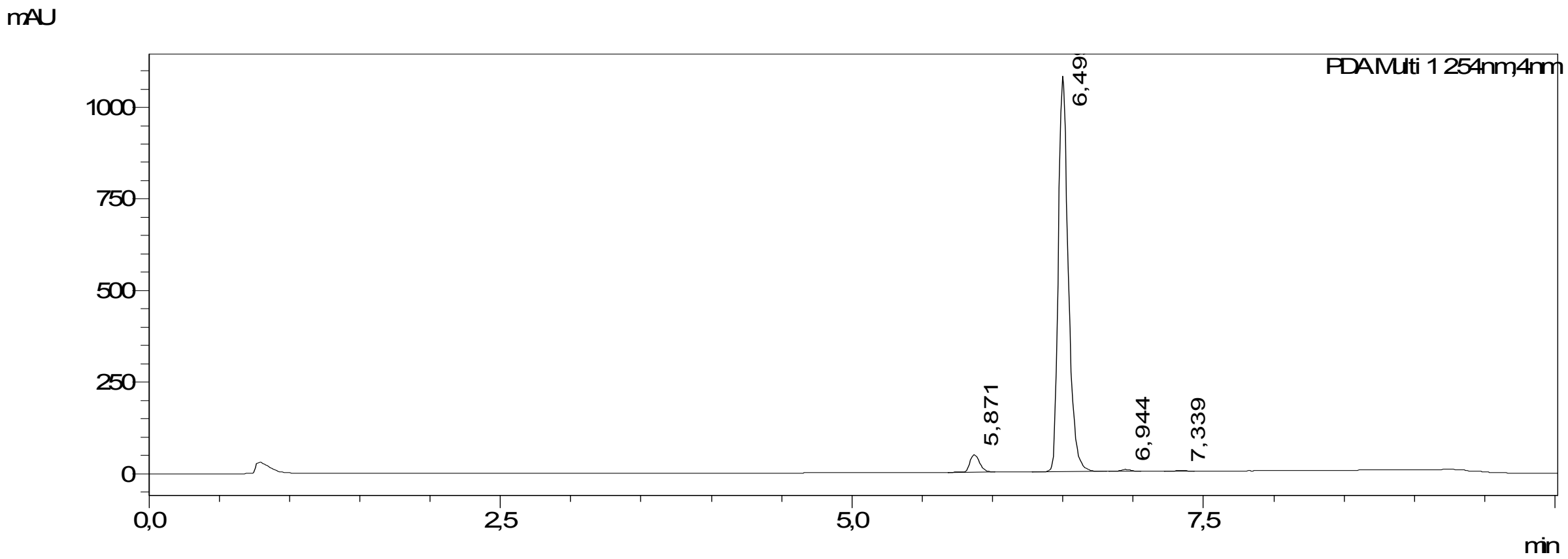

Figure S10. Chromatogram of (**10**); retention time at 7.113 minutes; 95.9% purity.

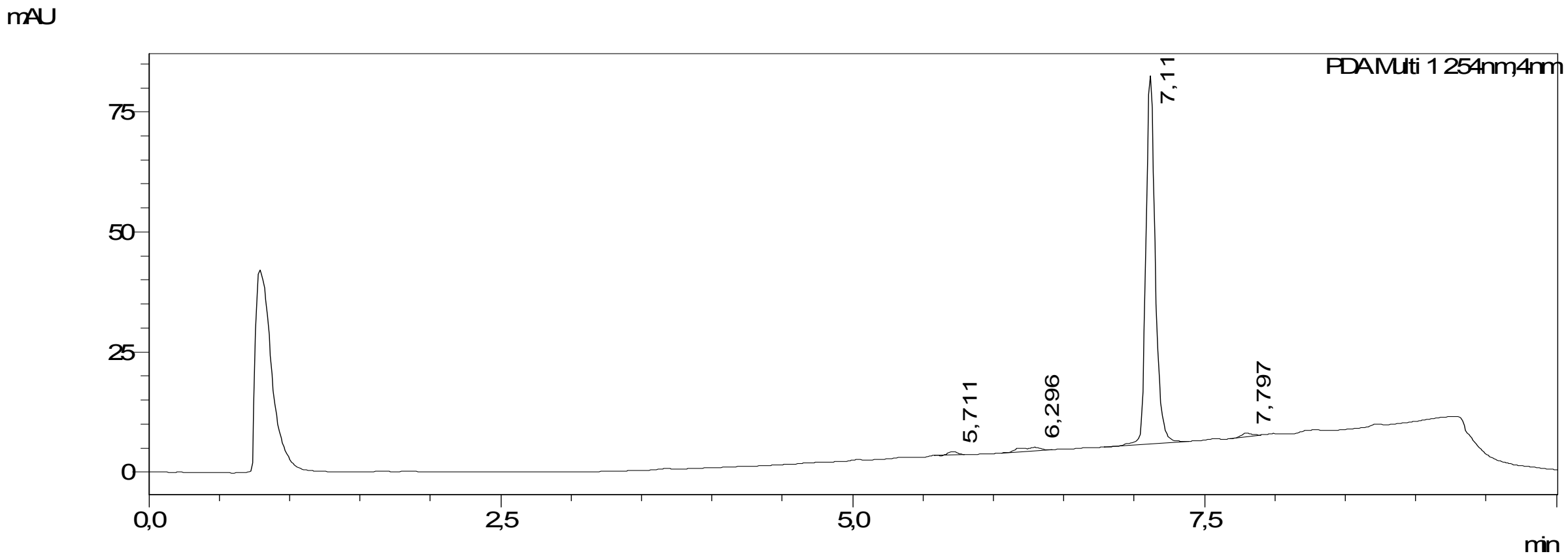

Figure S11. Chromatogram of (**11**); retention time at 7.500 minutes; 98.7% purity.

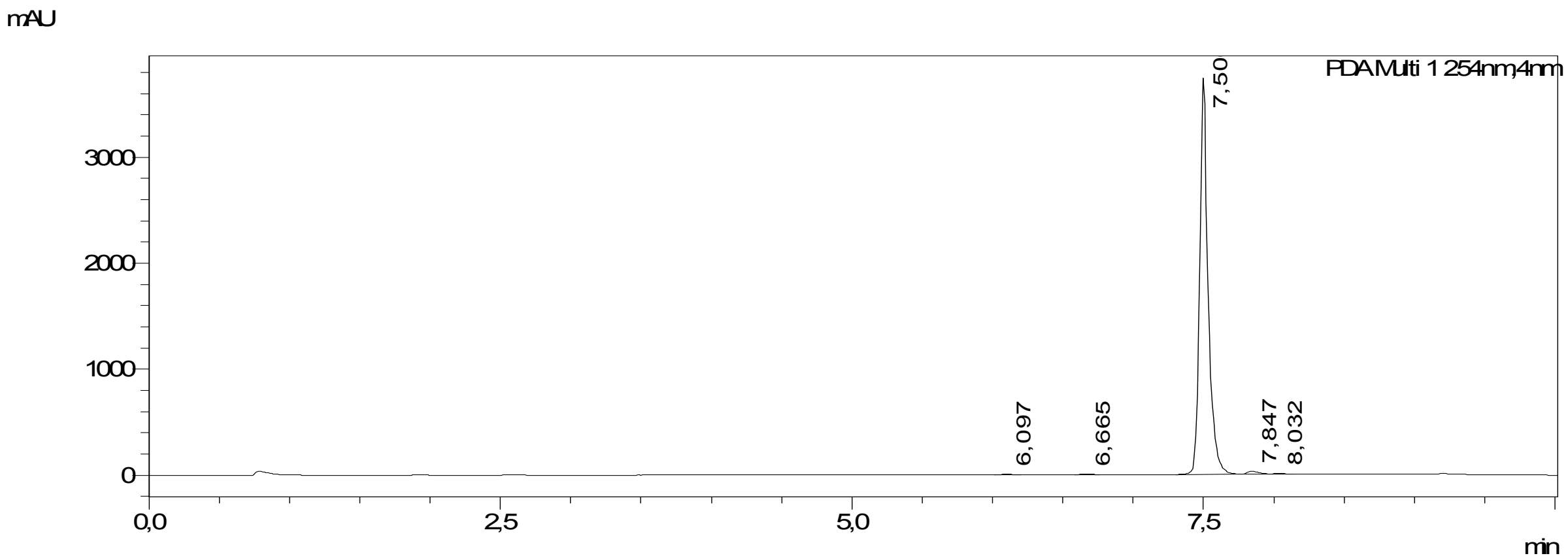

Figure S12. Chromatogram of (**12**); retention time at 6.520 minutes; 96.1% purity.

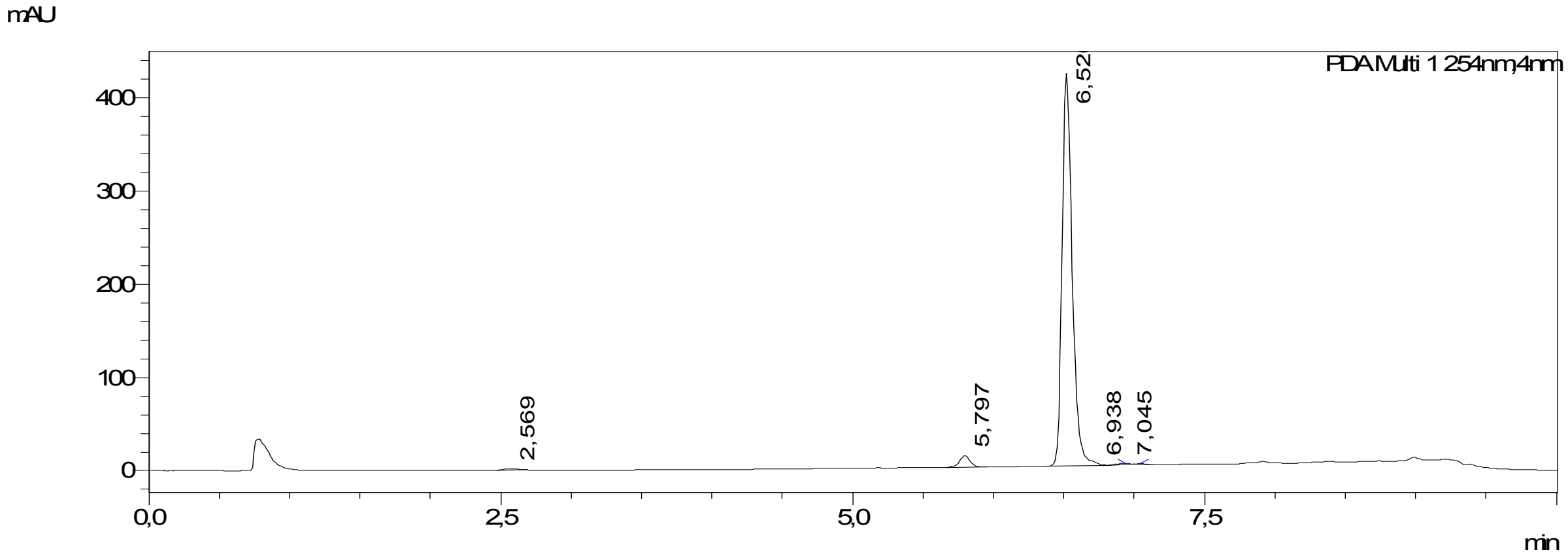

Figure S13. Chromatogram of (**13**); retention time at 7.131 minutes; 96.9% purity.

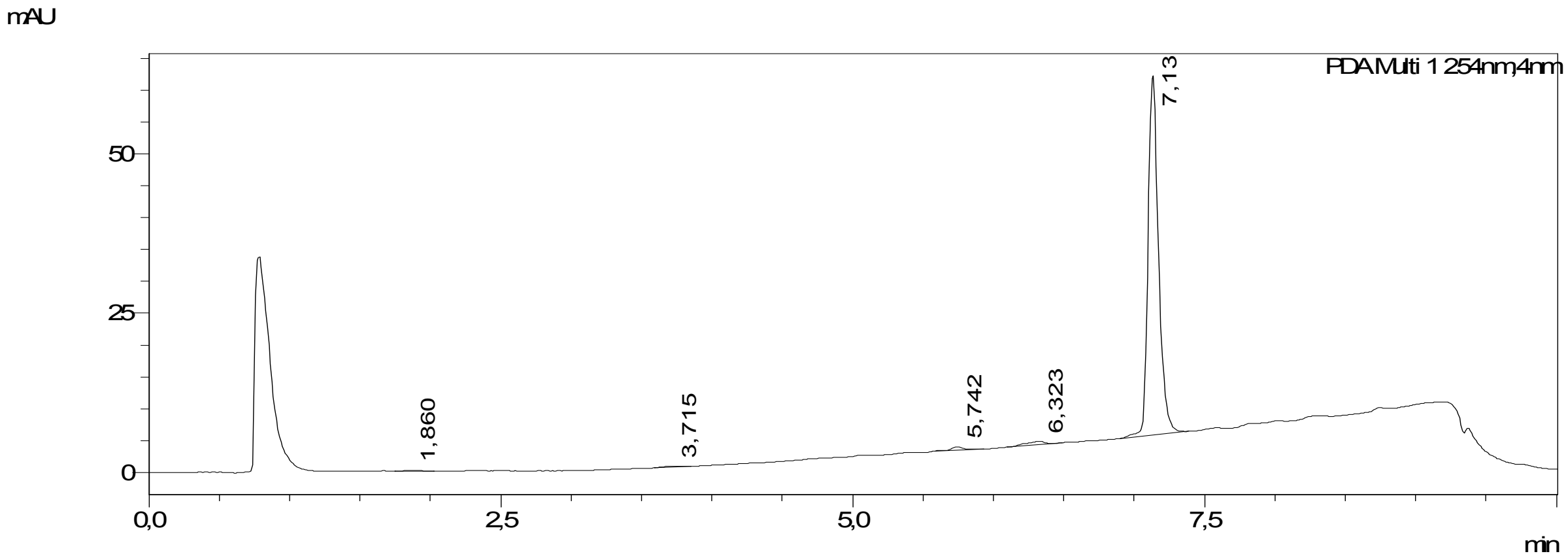

Figure S14. Chromatogram of (**14**); retention time at 7.016 minutes; 95.3% purity.

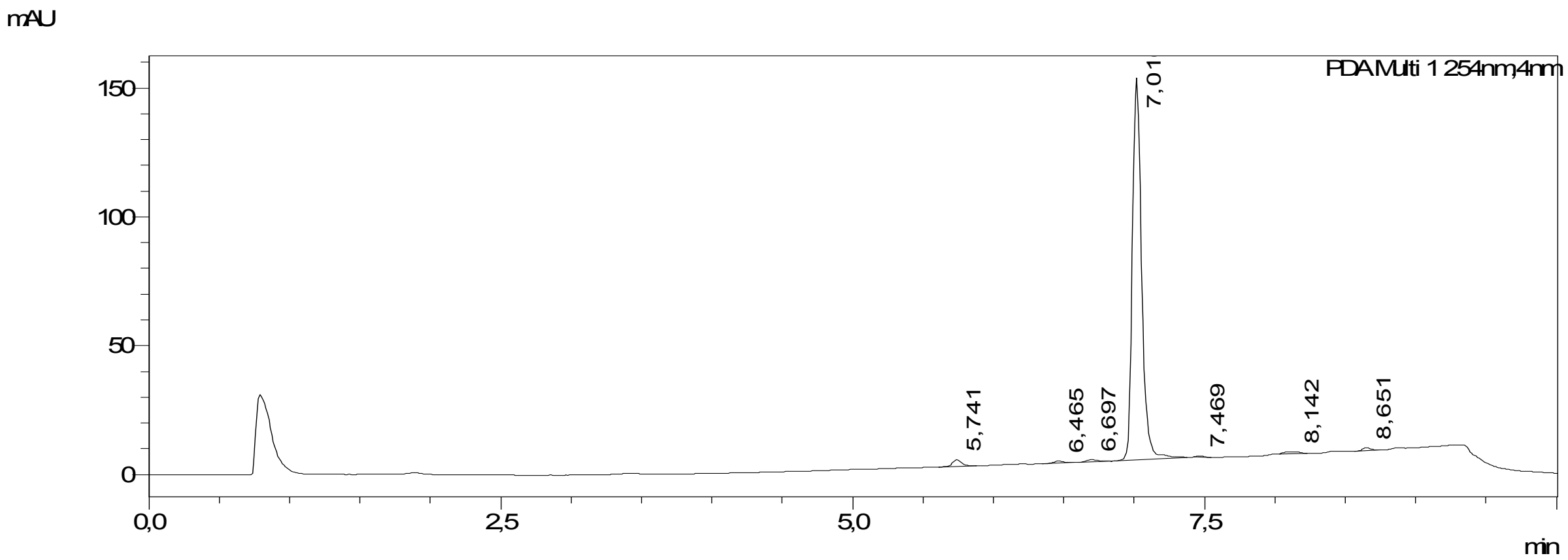

Figure S15. Chromatogram of (**15**); retention time at 6.775 minutes; 96.1% purity.

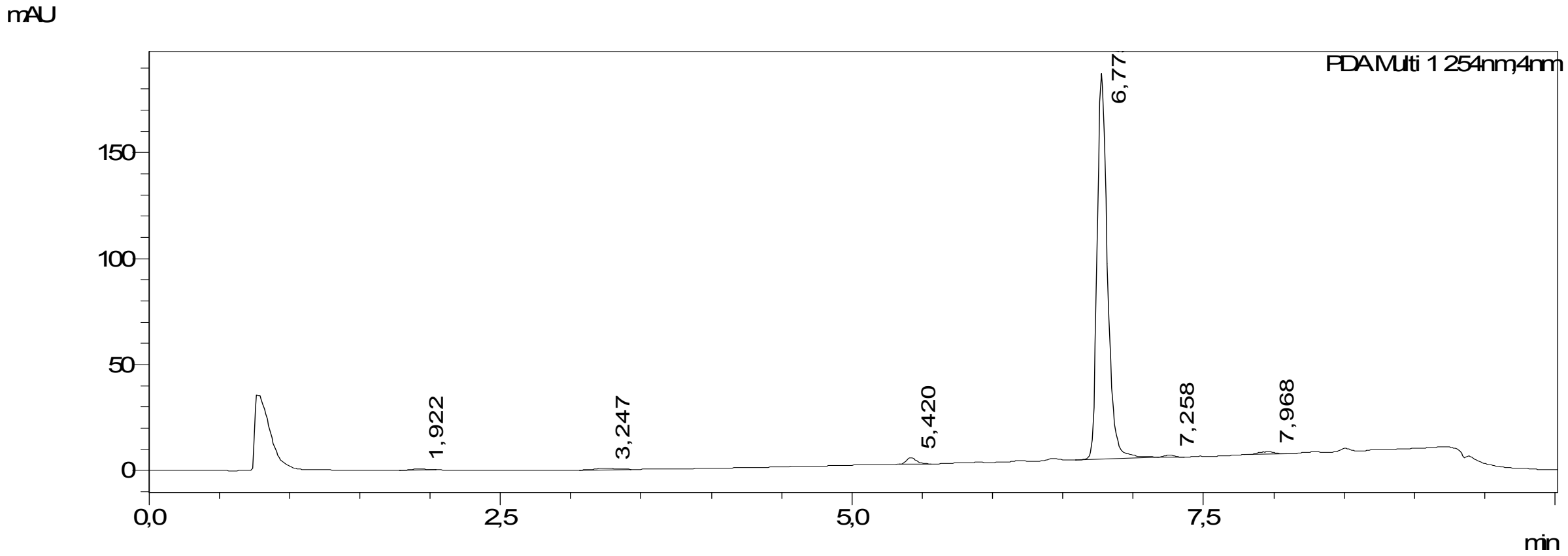

Figure S16. Chromatogram of (**16**); retention time at 7.696 minutes; 96.4% purity.

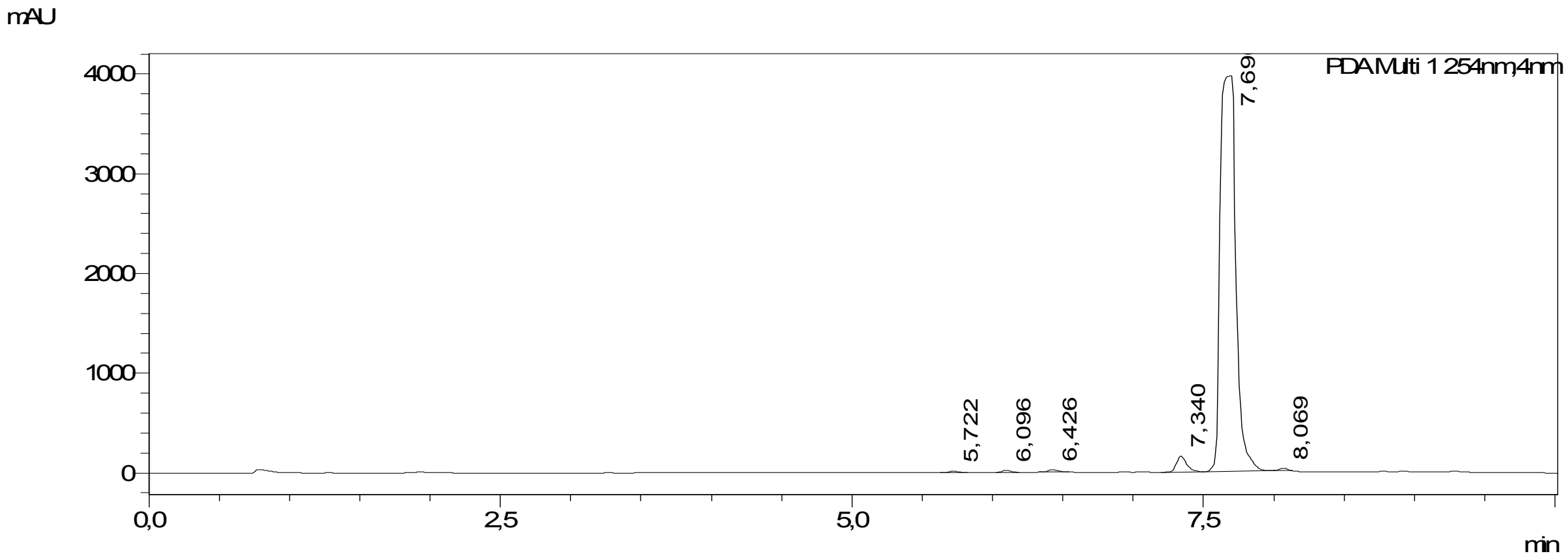

Figure S17. Chromatogram of (**17**); retention time at 6.402 minutes; 96.3% purity.

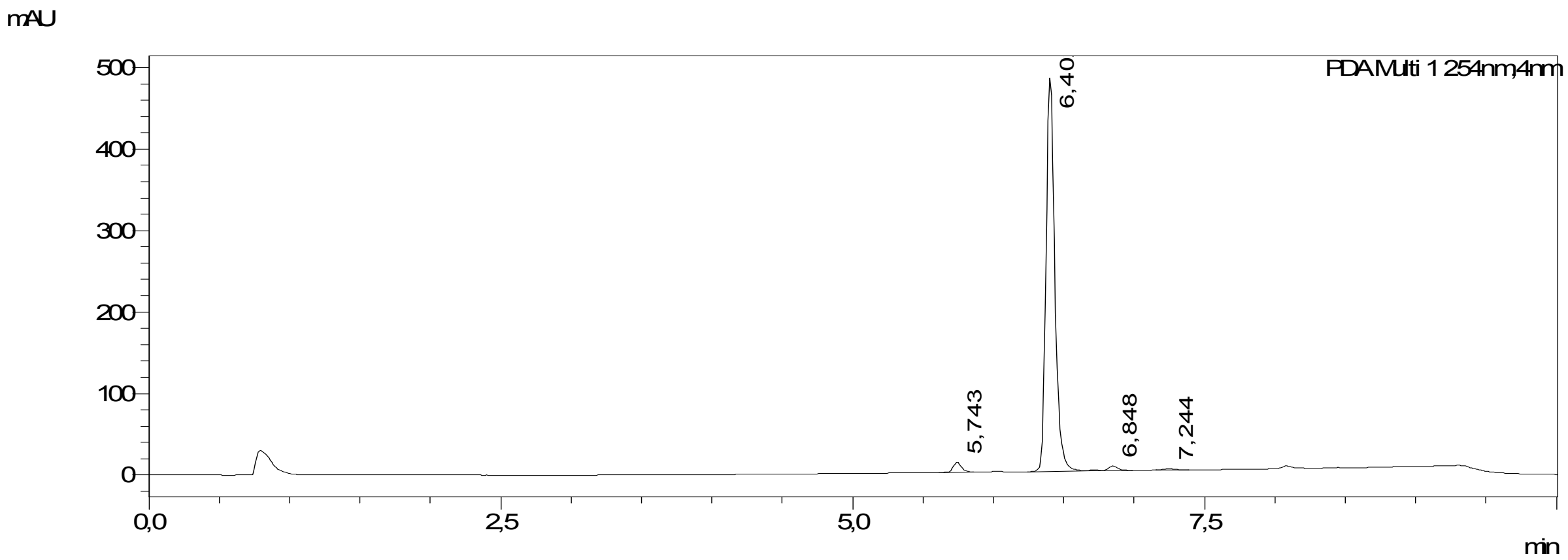

Figure S18. Chromatogram of (**18**); retention time at 6.119 minutes; 95.7% purity.

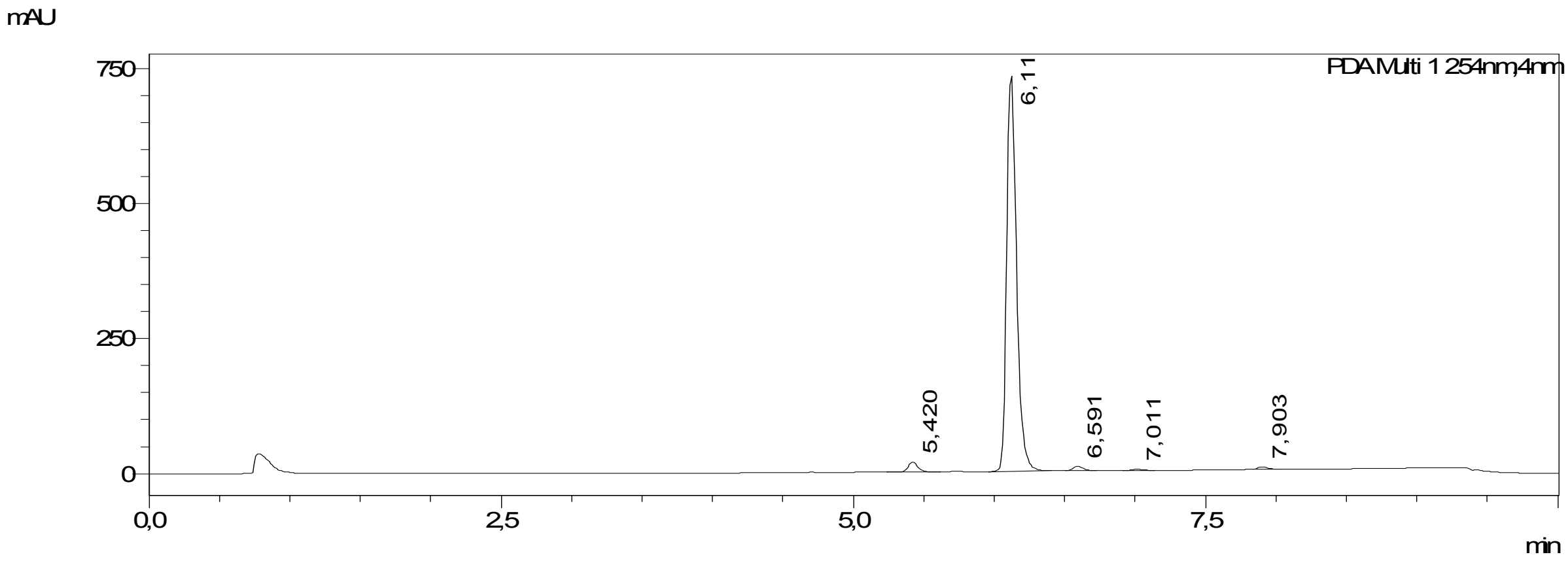

Figure S19. Chromatogram of (**19**); retention time at 6.675 minutes; 97.4% purity.

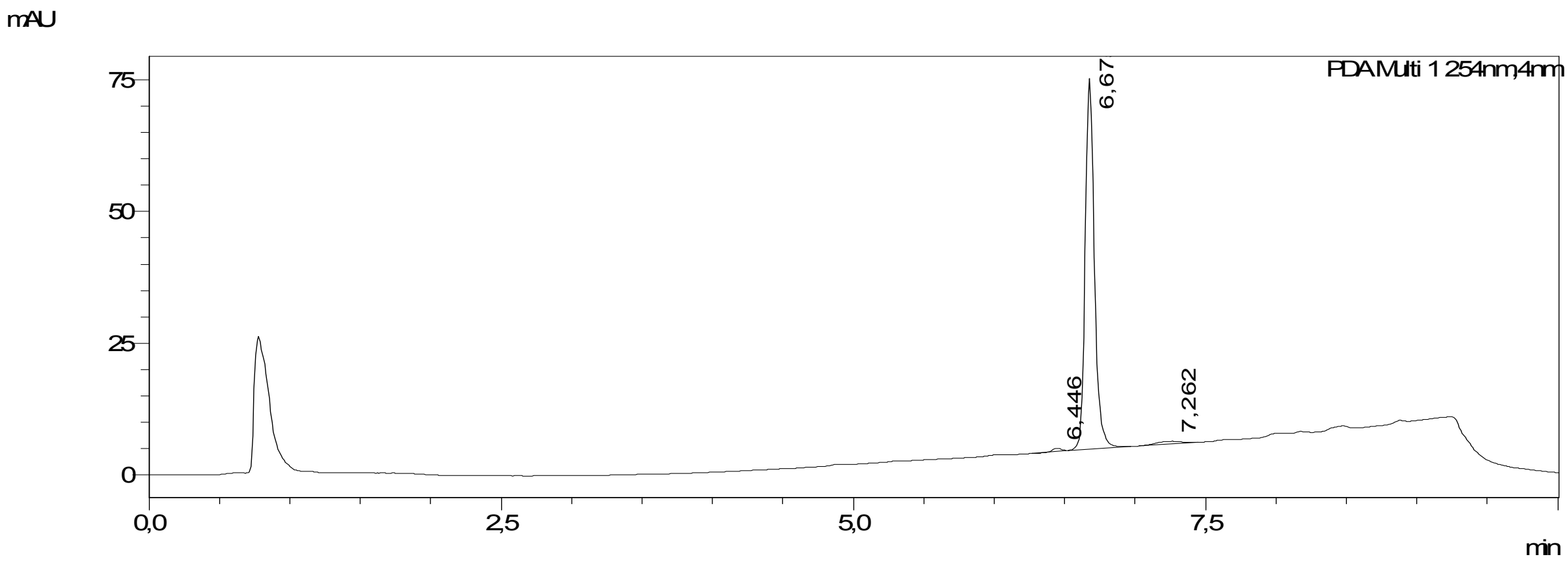

Figure S20. Chromatogram of (**20**); retention time at 6.846 minutes; 96.1% purity.

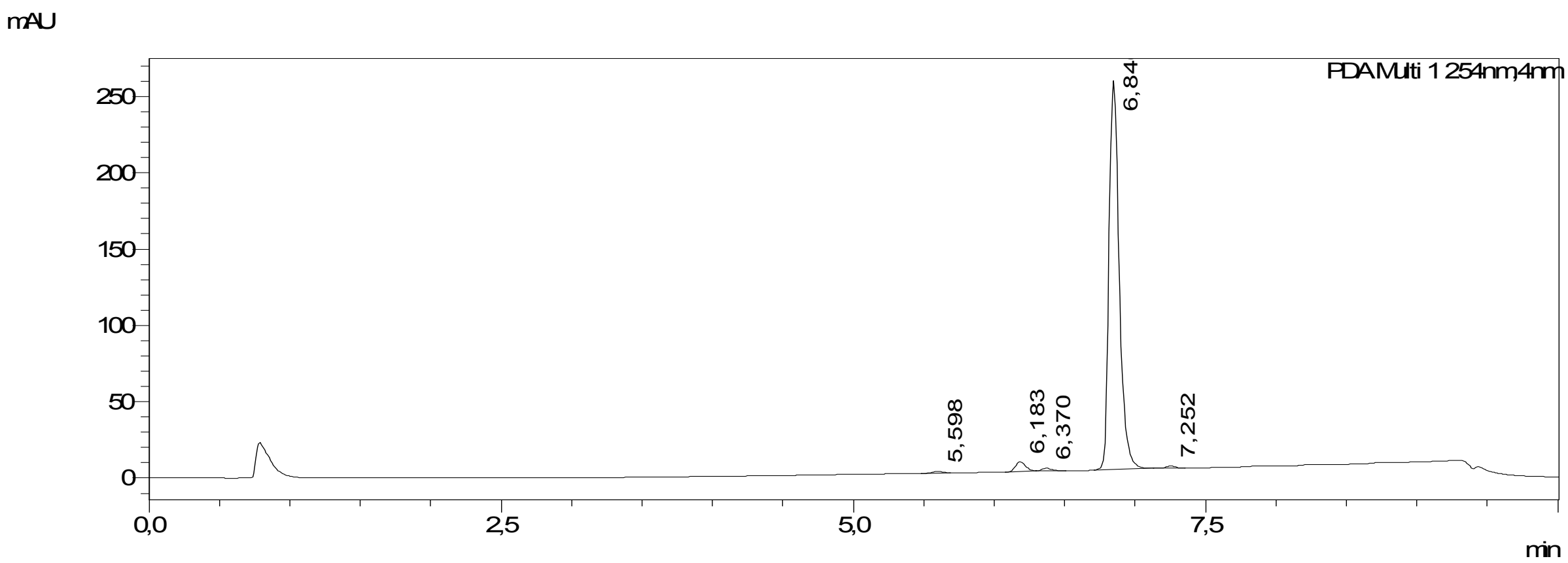

Figure S21. Chromatogram of (**21**); retention time at 6.775; 98.8% purity.

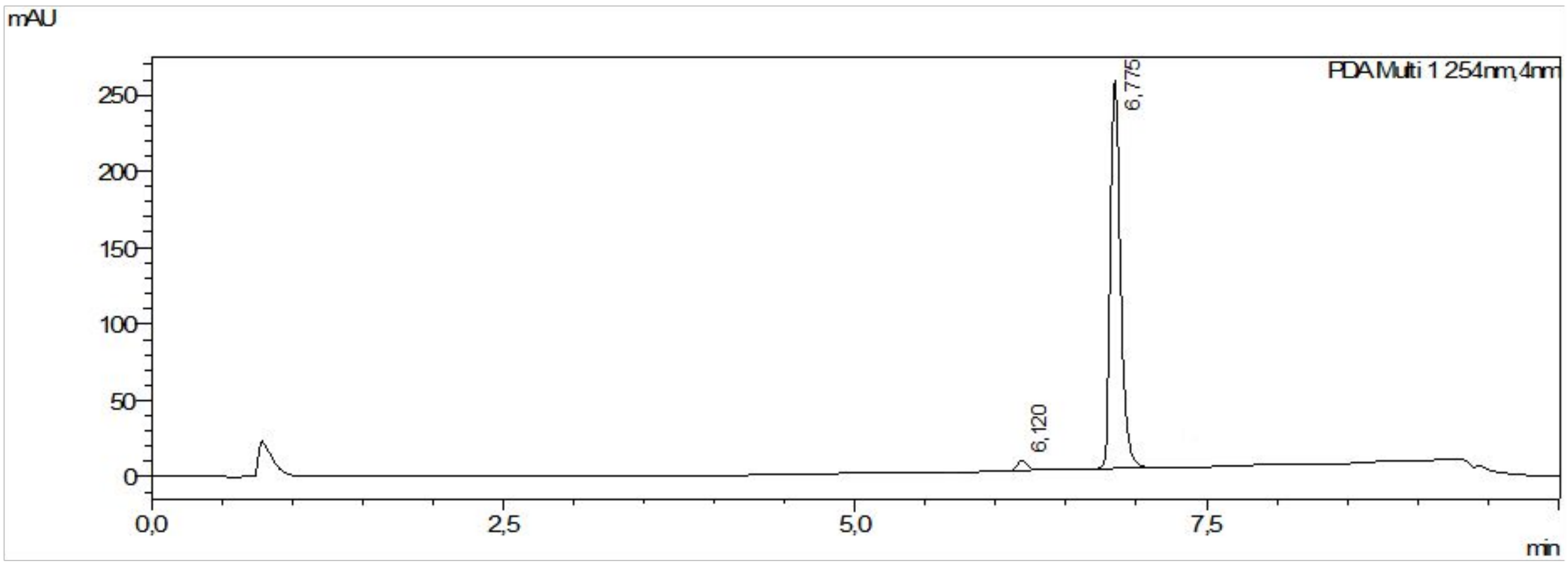

Figure S22. Chromatogram of (22); retention time at 6.423 minutes; 97.7% purity.

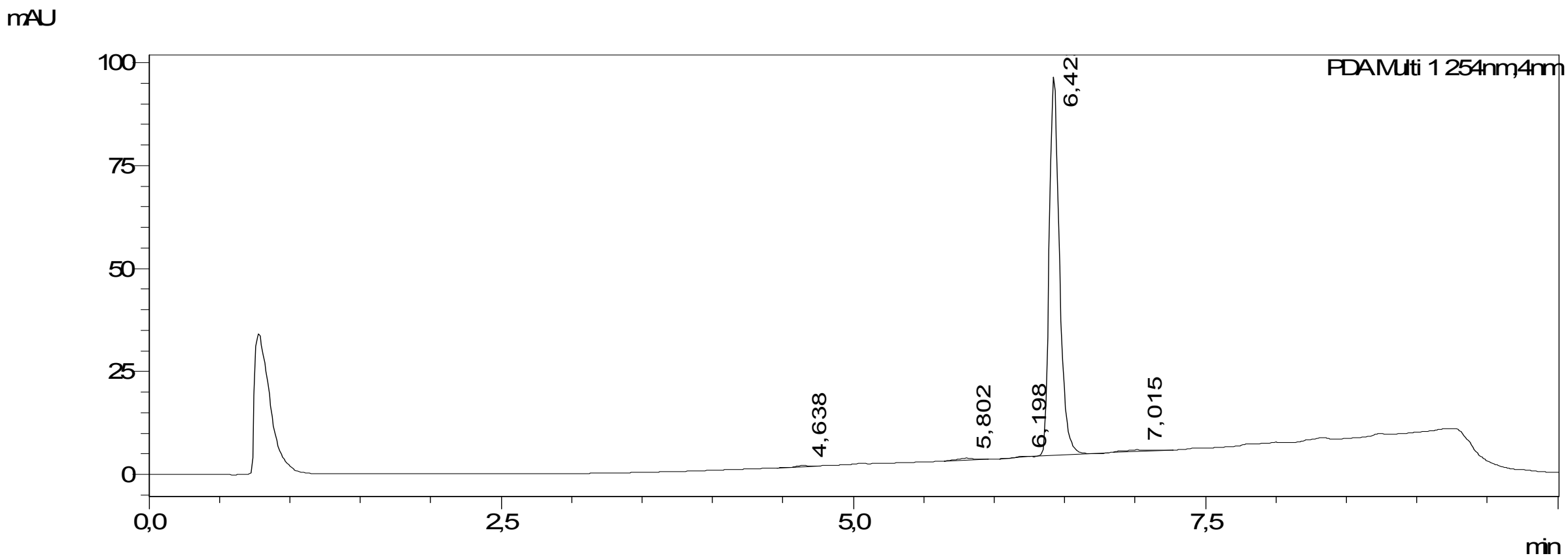

Figure S23. Chromatogram of (23); retention time at 6.535 minutes; 95.9% purity.

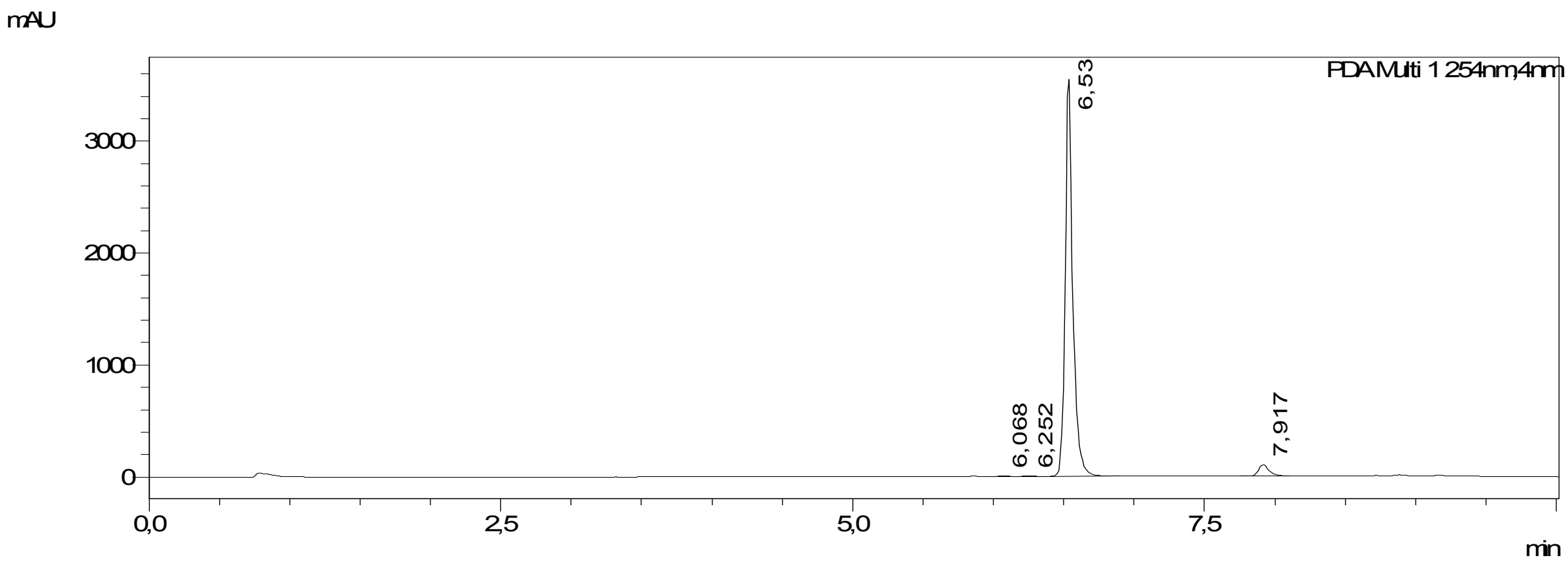

Figure S24. Chromatogram of (24); retention time at 5.265 minutes; 98.5% purity.

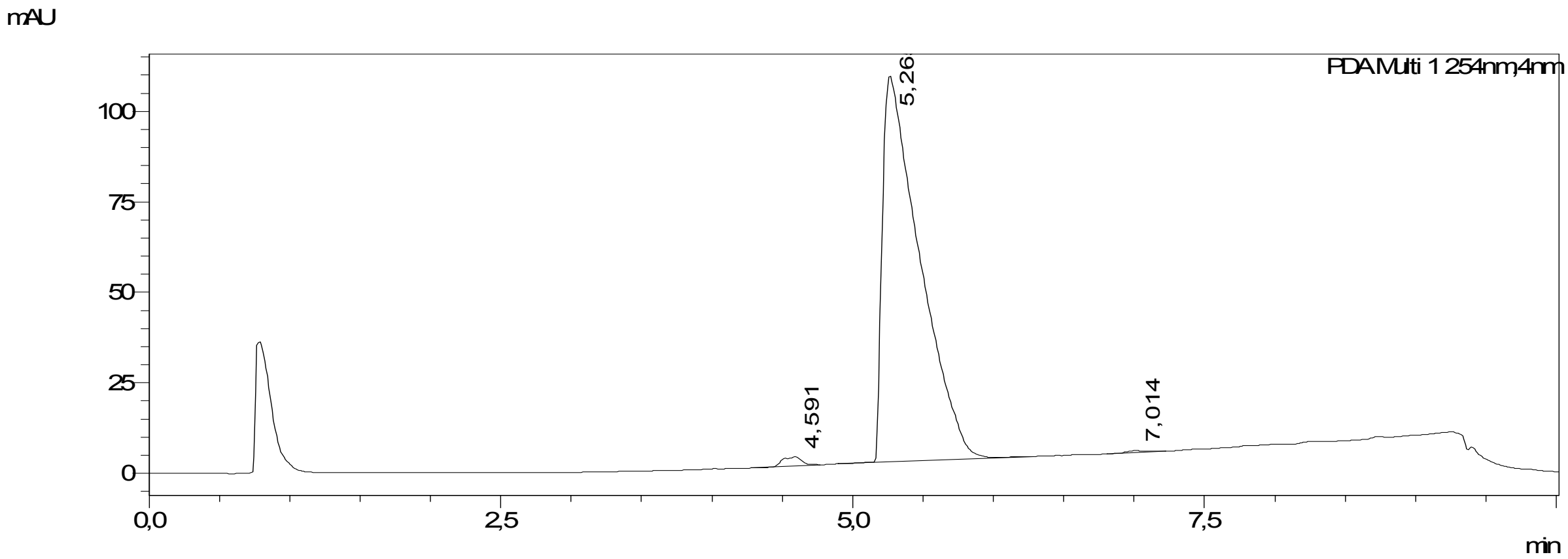

Figure S25. Chromatogram of (25); retention time at 4.655 minutes; 99.6% purity.

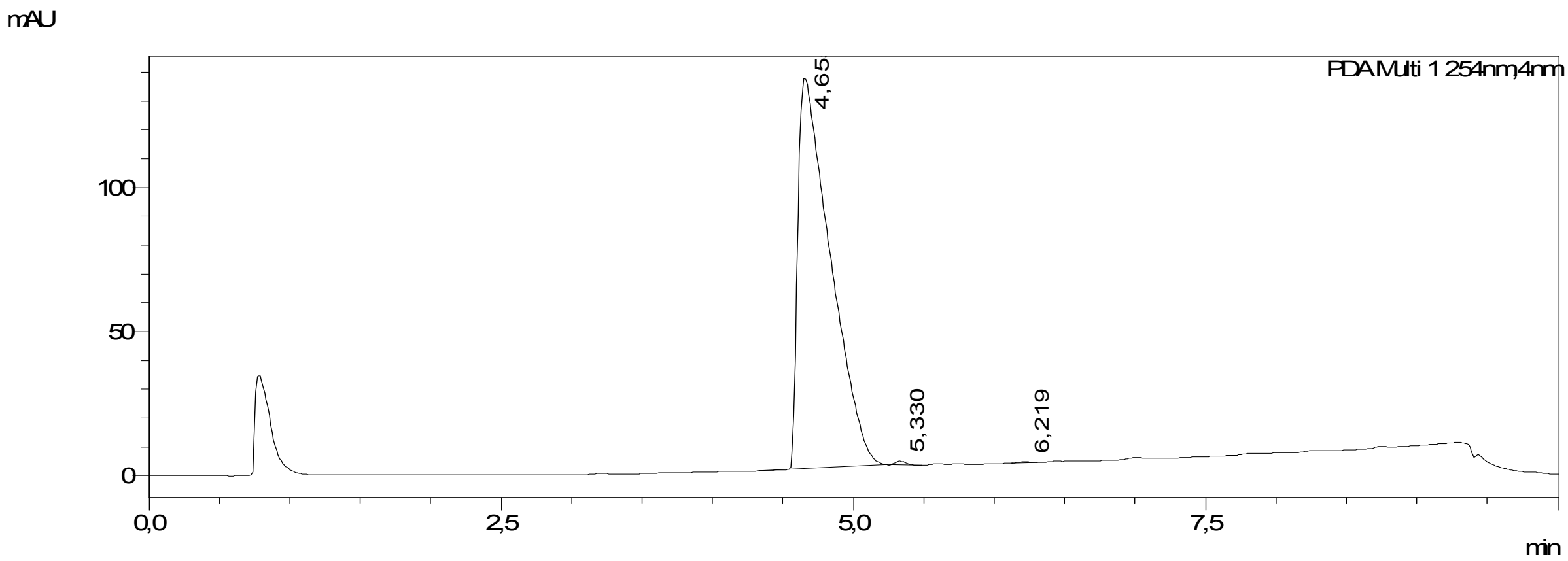

Figure S26. Chromatogram of (26); retention time at 5.158 minutes; 95.0% purity.

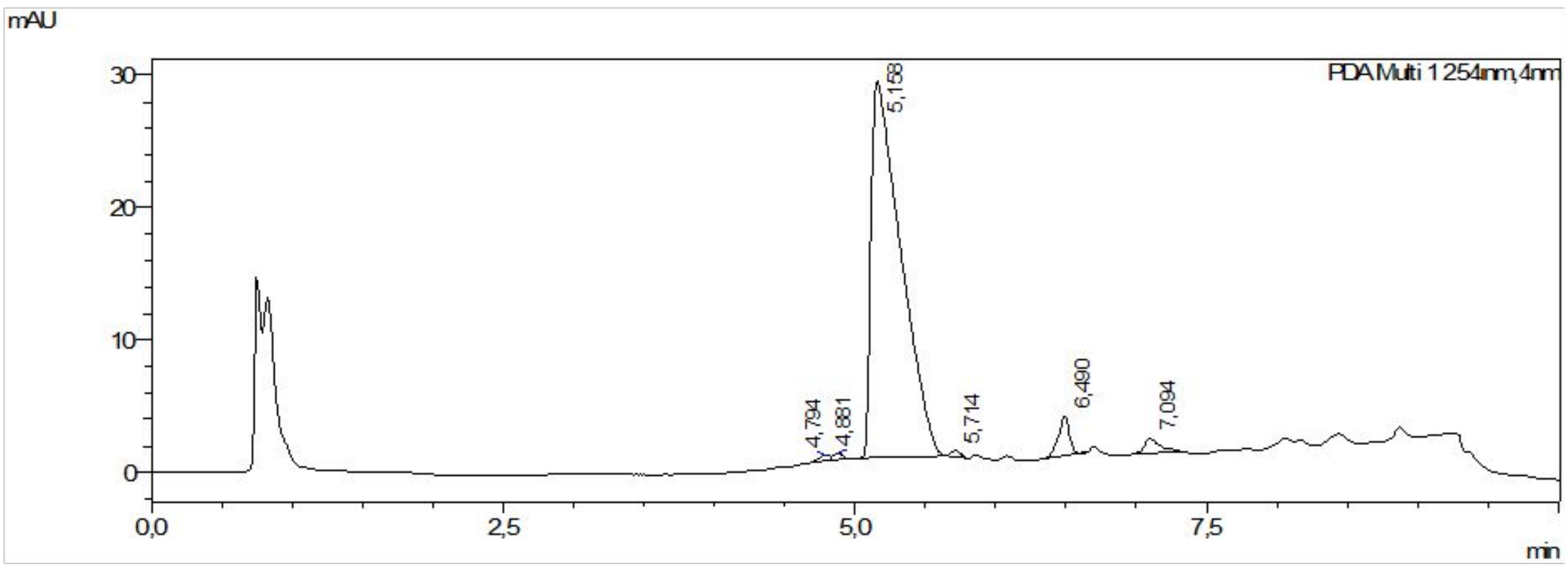

Figure S27. Chromatogram of Blank run; DMSO and water (v/v).

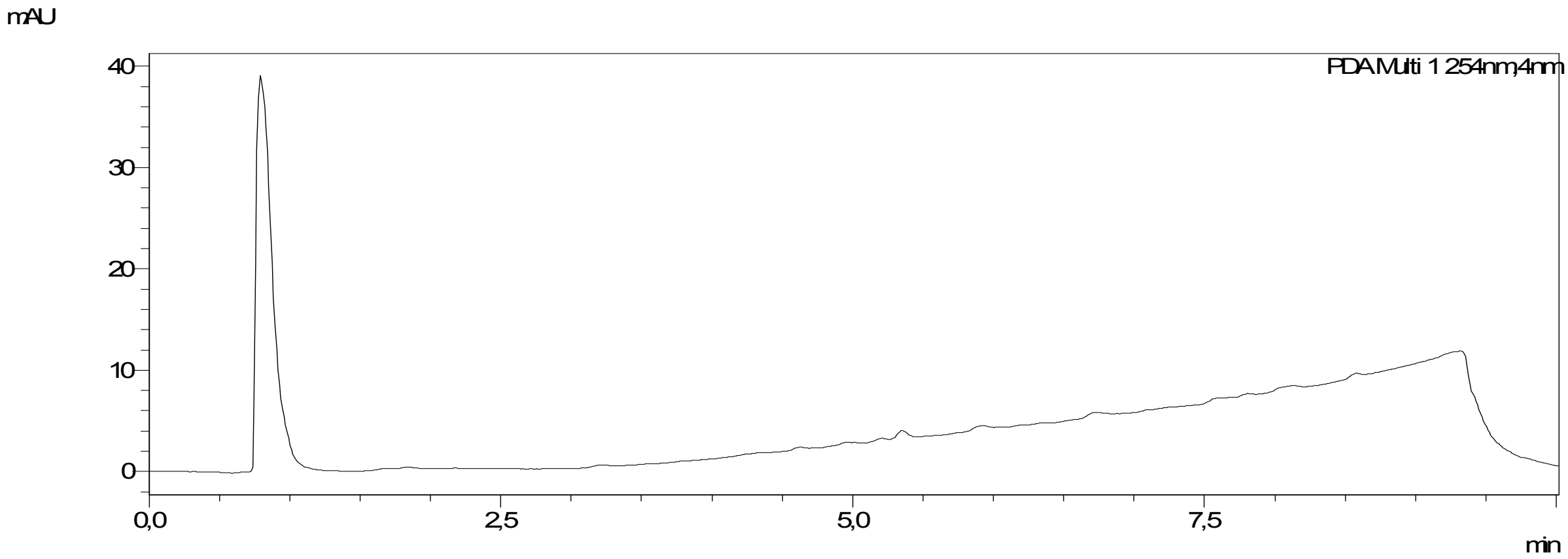

Table S1. Results of the incubation of (1) in human plasma over 24.00 h. Three samples were incubated over 24.00 h and measured at 254 nm with RP-UHPLC. The detected areas of (1) peaks were divided by the detected area of IS peaks (DOH). The average of the same time was converted to A(An)/A(IS). The calculated values referred to the recovered (1) peak area at t = 0.00 h to receive the percentage of the analyte (1) (n = 3).

	**Peak area**						
**Time [h]**	**(1)**	**Donepezil**	**A(Compound)/A(Standard)**	**A/A mean**	**Standard dev.**	**Var. [%]**	**Remaining compound [%]**
0	1920127	631932	3.039				
0	2041879	608252	3.357	3.209	0.160	5.0	100.0
0	1990499	616067	3.231				
0.5	1847418	625289	2.955				
0.5	2016192	613582	3.286	3.143	0.170	5.4	98.0
0.5	1938737	607942	3.189				
1	1850348	617711	2.995				
1	1891922	611295	3.095	3.135	0.163	5.2	97.7
1	1943635	586523	3.314				
1.5	1870581	594785	3.145				
1.5	1877820	609447	3.081	3.125	0.038	1.2	97.4
1.5	1895824	601847	3.150				
2	1830136	607846	3.011				
2	1904336	602066	3.163	3.115	0.090	2.9	97.1
2	1911174	602556	3.172				
4	1879078	615847	3.051				
4	1838624	606393	3.032	3.071	0.051	1.7	95.7
4	1901333	607743	3.129				
24	1753618	601943	2.913				
24	1743152	607381	2.870	2.873	0.039	1.4	89.5
24	1683638	593870	2.835				

Table S2. Results of the incubation of (1) in rat plasma over 24.00 h. Three samples were incubated over 24.00 h and measured at 254 nm with RP-UHPLC. The detected areas of (1) peaks were divided by the detected area of IS peaks (DOH). The average of the same time was converted to A(An)/A(IS). The calculated values referred to the recovered (1) peak area at t = 0.00 h to receive the percentage of the analyte (1) (n = 3).

	Peak area						
**Time [h]**	**(1)**	**Donepezil**	**A(Compound)/A(Standard)**	**A/A mean**	**Standard dev.**	**Var. [%]**	**Remaining compound [%]**
0	1044992	629553	1.660				
0	1144905	624980	1.832	1.788	0.113	6.3	100.0
0	1171680	625432	1.873				
0.5	1041567	625628	1.665				
0.5	1140112	623045	1.830	1.776	0.097	5.4	99.3
0.5	1151620	627704	1.835				
1	1001292	631086	1.587				
1	1158886	633996	1.828	1.758	0.149	8.5	98.3
1	1158886	623047	1.860				
1.5	1018845	633364	1.609				
1.5	1151280	630096	1.827	1.756	0.128	7.3	98.2
1.5	1154187	629773	1.833				
2	1017739	630020	1.615				
2	1153381	633901	1.819	1.754	0.121	6.9	98.1
2	1179567	645115	1.828				
4	1010523	637656	1.585				
4	1132781	629987	1.798	1.737	0.133	7.6	97.1
4	1156797	632695	1.828				
24	912201	631229	1.445				
24	851333	612498	1.390	1.346	0.127	9.4	75.3
24	722575	600361	1.204				

Table S3. Results of the incubation of (2) in human plasma over 24.00 h. Three samples were incubated over 24.00 h and measured at 254 nm with RP-UHPLC. The detected areas of (2) peaks were divided by the detected area of IS peaks (DOH). The average of the same time was converted to A(An)/A(IS). The calculated values referred to the recovered (2) peak area at t = 0.00 h to receive the percentage of the analyte (2) (n = 3).

	**Peak area**						
**Time [h]**	**(2)**	**Donepezil**	**A(Compound)/A(Standard)**	**A/A mean**	**Standard dev.**	**Var. [%]**	**Remaining compound [%]**
0	1759065	780371	2.254				
0	1824496	771216	2.366	2.313	0.056	2.4	100.0
0	1810270	780148	2.320				
0.5	1675218	773316	2.166				
0.5	1724847	777075	2.220	2.202	0.031	1.4	95.2
0.5	1717922	774143	2.219				
1	1762125	805567	2.187				
1	1711822	783228	2.186	2.158	0.049	2.3	93.3
1	1635574	778401	2.101				
1.5	1683594	786049	2.142				
1.5	1620926	783912	2.068	2.123	0.048	2.3	91.7
1.5	1714274	794378	2.158				
2	1620074	782123	2.071				
2	1670085	767122	2.177	2.109	0.059	2.8	91.2
2	1618342	778230	2.080				
4	1620341	786018	2.061				
4	1559140	743905	2.096	2.072	0.021	1.0	89.6
4	1619491	786710	2.059				
24	1567334	803174	1.951				
24	1622057	792346	2.047	1.981	0.057	2.9	85.6
24	1560585	802251	1.945				

Table S4. Results of the incubation of (2) in rat plasma over 24.00 h. Three samples were incubated over 24.00 h and measured with MS. The detected areas of (2) peaks were divided by the detected area of IS peaks (DOH). The average of the same time was converted to A(An)/A(IS). The calculated values were referred to the recovered (2) peak area at t = 0.00 h to receive the percentage of the analyte (2) (n = 3).

	Peak area						
**Time [h]**	**(2)**	**Donepezil**	**A(Compound)/A(Standard)**	**A/A mean**	**Standard dev.**	**Var. [%]**	**Remaining compound [%]**
0	22771642	66938544	0.340				
0	23265584	66408708	0.350	0.349	0.008	2.2	100.0
0	21444368	60330944	0.355				
0.5	23209806	69580072	0.334				
0.5	23464376	69728584	0.337	0.342	0.012	3.5	98.1
0.5	28195142	79298800	0.356				
1	26462346	80419680	0.329				
1	31721118	92783408	0.342	0.337	0.007	2.1	96.7
1	22579010	66408892	0.340				
1.5	21665318	68258272	0.317				
1.5	26362684	78194392	0.337	0.328	0.010	3.0	94.0
1.5	24824858	75495384	0.329				
2	25849320	81673048	0.316				
2	26623904	80319360	0.331	0.323	0.008	2.3	92.7
2	22822432	70919320	0.322				
4	21228298	68303720	0.311				
4	26103182	79293072	0.329	0.322	0.010	3.0	92.3
4	28621140	88054208	0.325				
24	19046460	79203352	0.240				
24	17241546	71770360	0.240	0.247	0.011	4.6	70.8
24	22046508	84769072	0.260				

Table S5. Results of the incubation of (3) in human plasma over 24.00 h. Three samples were incubated over 24.00 h and measured at 330 nm with RP-UHPLC. The detected areas of (3) peaks were divided by the detected area of IS peaks (DOH). The average of the same time was converted to A(An)/A(IS). The calculated values were referred to the recovered (3) peak area at t = 0.00 h to receive the percentage of the analyte (3) (n = 3).

	Peak area						
**Time [h]**	**(3)**	**Donepezil**	**A(Compound)/A(Standard)**	**A/A mean**	**Standard dev.**	**Var. [%]**	**Remaining compound [%]**
0	151709	786907	0.193				
0	152421	786222	0.194	0.193	0.001	0.5	100.0
0	150478	784341	0.192				
0.5	143504	791949	0.181				
0.5	145312	801762	0.181	0.181	0.001	0.3	93.8
0.5	143702	797080	0.180				
1	141020	796257	0.177				
1	140872	794605	0.177	0.178	0.001	0.4	92.1
1	142200	797355	0.178				
1.5	139171	799032	0.174				
1.5	139226	793337	0.175	0.175	0.001	0.4	90.7
1.5	137657	787052	0.175				
2	137299	796954	0.172				
2	138530	796441	0.174	0.172	0.002	1.0	89.3
2	136335	799962	0.170				
4	137410	804092	0.171				
4	137201	802647	0.171	0.172	0.001	0.7	89.0
4	139133	804590	0.173				
24	130046	790032	0.165				
24	136773	794352	0.172	0.169	0.004	2.3	87.7
24	134544	789550	0.170				

Table S6. Results of the incubation of (3) in rat plasma over 24.00 h. Three samples were incubated over 24.00 h and measured at 330 nm with RP-UHPLC. The detected areas of (3) peaks were divided by the detected area of IS peaks (DOH). The average of the same time was converted to A(An)/A(IS). The calculated values were referred to the recovered (3) peak area at t = 0.00 h to receive the percentage of the analyte (3) (n = 3).

	Peak area						
**Time [h]**	**(3)**	**Donepezil**	**A(Compound)/A(Standard)**	**A/A mean**	**Standard dev.**	**Var. [%]**	**Remaining compound [%]**
0	225252	614892	0.366				
0	241562	616701	0.392	0.386	0.017	4.4	100.0
0	245156	614901	0.399				
0.5	213708	617264	0.346				
0.5	236014	614051	0.384	0.374	0.024	6.4	96.9
0.5	239577	613525	0.390				
1	210974	613858	0.344				
1	225490	618001	0.365	0.364	0.019	5.3	94.3
1	237165	620856	0.382				
1.5	214882	617645	0.348				
1.5	222330	618809	0.359	0.356	0.007	1.9	92.2
1.5	222146	617645	0.360				
2	216518	621004	0.349				
2	216727	624964	0.347	0.355	0.012	3.5	92.0
2	229357	621412	0.369				
4	222755	616854	0.348				
4	219695	613455	0.358	0.354	0.005	1.5	91.8
4	222769	626755	0.355				
24	205510	624111	0.329				
24	187651	638089	0.294	0.318	0.021	6.5	82.5
24	206350	623688	0.331				

Table S7. Results of the incubation of (4) in human plasma over 24.00 h. Three samples were incubated over 24.00 h and measured at 230 nm with RP-UHPLC. The detected areas of (4) peaks were divided by the detected area of IS peaks (DOH). The average of the same time was converted to A(An)/A(IS). The calculated values were referred to the recovered (4) peak area at t = 0.00 h to receive the percentage of the analyte (4) (n = 3).

	Peak area						
**Time [h]**	**(4)**	**Donepezil**	**A(Compound)/A(Standard)**	**A/A mean**	**Standard dev.**	**Var. [%]**	**Remaining compound [%]**
0	593382	2065581	0.287				
0	593576	2027828	0.293	0.291	0.004	1.2	100.0
0	601019	2045160	0.294				
0.5	587121	2055967	0.286				
0.5	589705	2070066	0.285	0.285	0.001	0.3	97.8
0.5	584354	2058385	0.284				
1	564757	2064403	0.274				
1	569506	2059134	0.277	0.275	0.002	0.6	94.3
1	561537	2049400	0.274				
1.5	549911	2090897	0.263				
1.5	571386	2092001	0.273	0.273	0.009	3.4	93.6
1.5	583715	2073352	0.282				
2	551851	2056633	0.268				
2	552656	2062989	0.268	0.270	0.002	0.9	92.5
2	558142	2049263	0.272				
4	565395	2051304	0.276				
4	541654	2082445	0.260	0.267	0.008	3.0	91.6
4	552176	2087045	0.265				
24	531236	2098833	0.253				
24	555110	2061833	0.269	0.264	0.009	3.5	90.6
24	557930	2071434	0.269				

Table S8. Results of the incubation of (4) in rat plasma over 24.00 h. Three samples were incubated over 24.00 h and measured at 230 nm with RP-UHPLC. The detected areas of (4) peaks were divided by the detected area of IS peaks (DOH). The average of the same time was converted to A(An)/A(IS). The calculated values were referred to the recovered (4) peak area at t = 0.00 h to receive the percentage of the analyte (4) (n = 3).

	Peak area						
**Time [h]**	**(4)**	**Donepezil**	**A(Compound)/A(Standard)**	**A/A mean**	**Standard dev.**	**Var. [%]**	**Remaining compound [%]**
0	880691	1637003	0.538				
0	922557	1606318	0.574	0.569	0.029	5.2	100.0
0	942282	1580822	0.596				
0.5	865129	1608732	0.538				
0.5	907652	1613625	0.562	0.558	0.019	3.4	98.1
0.5	901193	1567649	0.575				
1	866614	1619393	0.535				
1	905799	1580098	0.573	0.557	0.020	3.6	97.9
1	895801	1588059	0.564				
1.5	869005	1601996	0.542				
1.5	889290	1589312	0.560	0.555	0.011	2.0	97.5
1.5	902148	1599637	0.564				
2	868967	1617327	0.537				
2	879443	1618767	0.543	0.547	0.012	2.1	96.0
2	895654	1600038	0.560				
4	857009	1600943	0.535				
4	868936	1594280	0.545	0.541	0.005	1.0	95.0
4	861986	1586843	0.543				
24	845300	1634165	0.517				
24	785975	1606405	0.489	0.503	0.014	2.8	88.4
24	812734	1613236	0.504				

Table S9. Results of the incubation of (5) in human plasma over 24.00 h. Three samples were incubated over 24.00 h and measured at 254 nm with RP-UHPLC. The detected areas of (5) peaks were divided by the detected area of IS peaks (DOH). The average of the same time was converted to A(An)/A(IS). The calculated values were referred to the recovered (5) peak area at t = 0.00 h to receive the percentage of the analyte (5) (n = 3).

	Peak area						
**Time [h]**	**(5)**	**Donepezil**	**A(Compound)/A(Standard)**	**A/A mean**	**Standard dev.**	**Var. [%]**	**Remaining compound [%]**
0	1495877	774085	1.932				
0	1496710	772126	1.938	1.932	0.007	0.4	100.0
0	1495042	776840	1.925				
0.5	1474240	770674	1.913				
0.5	1478813	776696	1.904	1.916	0.013	0.7	99.2
0.5	1485658	769617	1.930				
1	1471859	766175	1.921				
1	1476288	777503	1.899	1.909	0.011	0.6	98.8
1	1473874	772198	1.909				
1.5	1464545	771698	1.898				
1.5	1468709	767642	1.913	1.904	0.008	0.4	98.5
1.5	1466294	771829	1.900				
2	1468476	771702	1.903				
2	1452863	773339	1.879	1.890	0.012	0.6	97.8
2	1462982	774769	1.888				
4	1456605	777056	1.875				
4	1459698	767314	1.902	1.888	0.014	0.7	97.7
4	1461676	775034	1.886				
24	1468965	778292	1.887				
24	1447198	775178	1.867	1.877	0.010	0.5	97.2
24	1455870	775178	1.878				

Table S10. Results of the incubation of (5) in rat plasma over 24.00 h. Three samples were incubated over 24.00 h and measured at 254 nm with RP-UHPLC. The detected areas of (5) peaks were divided by the detected area of IS peaks (DOH). The average of the same time was converted to A(An)/A(IS). The calculated values were referred to the recovered (5) peak area at t = 0.00 h to receive the percentage of the analyte (5) (n = 3).

	**Peak area**						
**Time [h]**	**(5)**	**Donepezil**	**A(Compound)/A(Standard)**	**A/A mean**	**Standard dev.**	**Var. [%]**	**Remaining compound [%]**
0	1108070	521744	2.124				
0	1023962	483220	2.119	2.071	0.086	4.2	100.0
0	985049	499600	1.972				
0.5	990955	505323	1.961				
0.5	1060828	528912	2.006	1.986	0.023	1.1	95.9
0.5	1011733	507993	1.992				
1	1312783	667775	1.966				
1	1002328	529829	1.892	1.947	0.049	2.5	94.0
1	956708	482146	1.984				
1.5	945247	505428	1.870				
1.5	969798	502818	1.929	1.921	0.048	2.5	92.8
1.5	852481	433781	1.965				
2	717788	410946	1.747				
2	874321	469045	1.864	1.797	0.061	3.4	86.7
2	762850	428810	1.779				
4	634857	366078	1.734				
4	596403	332395	1.794	1.764	0.030	1.7	85.2
4	586201	332217	1.765				
24	1244054	769926	1.616				
24	1330201	805432	1.652	1.628	0.020	1.2	78.6
24	1293294	799502	1.618				

Table S11. Results of the incubation of (6) in human plasma over 24.00 h. Three samples were incubated over 24.00 h and measured at 254 nm with RP-UHPLC. The detected areas of (6) peaks were divided by the detected area of IS peaks (DOH). The average of the same time was converted to A(An)/A(IS). The calculated values were referred to the recovered (6) peak area at t = 0.00 h to receive the percentage of the analyte (6) (n = 3).

	Peak area						
**Time [h]**	**(6)**	**Donepezil**	**A(Compound)/A(Standard)**	**A/A mean**	**Standard dev.**	**Var. [%]**	**Remaining compound [%]**
0	1617781	764297	2.117				
0	1617781	772015	2.096	2.104	0.011	0.5	100.0
0	1618407	770904	2.099				
0.5	1618560	768136	2.107				
0.5	1597264	774739	2.062	2.087	0.023	1.1	99.2
0.5	1610441	769473	2.093				
1	1593833	768136	2.075				
1	1593216	768717	2.073	2.084	0.018	0.9	99.1
1	1608557	764288	2.105				
1.5	1585554	783864	2.023				
1.5	1594046	774152	2.059	2.064	0.044	2.1	98.1
1.5	1616114	765680	2.111				
2	1594427	765162	2.084				
2	1529491	793363	1.928	2.024	0.084	4.2	96.2
2	1598670	775788	2.061				
4	1578563	781935	2.019				
4	1575572	780618	2.018	2.018	0.001	0.1	95.9
4	1575875	781392	2.017				
24	1585309	801077	1.979				
24	1560504	779988	2.001	1.998	0.017	0.9	95.0
24	1573285	781392	2.013				

Table S12. Results of the incubation of (6) in rat plasma over 24.00 h. Three samples were incubated over 24.00 h and measured at 254 nm with RP-UHPLC. The detected areas of (6) peaks were divided by the detected area of IS peaks (DOH). The average of the same time was converted to A(An)/A(IS). The calculated values were referred to the recovered (6) peak area at t = 0.00 h to receive the percentage of the analyte (6) (n = 3).

	Peak area						
**Time [h]**	**(6)**	**Donepezil**	**A(Compound)/A(Standard)**	**A/A mean**	**Standard dev.**	**Var. [%]**	**Remaining compound [%]**
0	1463088	619173	2.363				
0	1460920	610354	2.394	2.373	0.018	0.7	100.0
0	1459795	617783	2.363				
0.5	1377540	616689	2.234				
0.5	1390917	612399	2.271	2.259	0.022	1.0	95.2
0.5	1406699	619468	2.271				
1	1391779	615089	2.263				
1	1405734	627218	2.241	2.254	0.011	0.5	95.0
1	1408159	623481	2.259				
1.5	1394938	628102	2.221				
1.5	1407009	619346	2.272	2.250	0.026	1.2	94.8
1.5	1400834	620238	2.259				
2	1394452	630020	2.213				
2	1388687	609680	2.278	2.250	0.033	1.5	94.8
2	1390562	615925	2.258				
4	1388661	625756	2.219				
4	1394323	626042	2.227	2.224	0.004	0.2	93.7
4	1394323	626277	2.226				
24	1350034	616518	2.190				
24	1356532	617709	2.196	2.191	0.004	0.2	92.3
24	1352231	617978	2.188				

Table S13. Results of the incubation of (7) in human plasma over 24.00 h. Three samples were incubated over 24.00 h and measured at 254 nm with RP-UHPLC. The detected areas of (7) peaks were divided by the detected area of IS peaks (DOH). The average of the same time was converted to A(An)/A(IS). The calculated values were referred to the recovered (7) peak area at t = 0.00 h to receive the percentage of the analyte (7) (n = 3).

	**Peak area**						
**Time [h]**	**(7)**	**Donepezil**	**A(Compound)/A(Standard)**	**A/A mean**	**Standard dev.**	**Var. [%]**	**Remaining compound [%]**
0	314646	778217	0.404				
0	319557	772918	0.413	0.407	0.005	1.3	100.0
0	314646	778217	0.404				
0.5	312269	776592	0.402				
0.5	308043	773002	0.399	0.400	0.002	0.5	98.2
0.5	310018	776644	0.399				
1	309544	774967	0.399				
1	303598	762975	0.398	0.397	0.004	0.9	97.3
1	301836	769389	0.392				
1.5	303236	779692	0.389				
1.5	304120	780682	0.390	0.391	0.002	0.6	95.9
1.5	299994	762643	0.393				
2	300640	765655	0.393				
2	298675	782909	0.381	0.385	0.006	1.6	94.6
2	293812	768517	0.382				
4	252117	680940	0.370				
4	294854	775661	0.380	0.378	0.007	1.9	92.9
4	296178	770010	0.385				
24	280032	793767	0.353				
24	295025	787182	0.375	0.364	0.011	3.0	89.4
24	291696	798135	0.365				

Table S14. Results of the incubation of (7) in rat plasma over 24.00 h. Three samples were incubated over 24.00 h and measured at 254 nm with RP-UHPLC. The detected areas of (7) peaks were divided by the detected area of IS peaks (DOH). The average of the same time was converted to A(An)/A(IS). The calculated values were referred to the recovered (7) peak area at t = 0.00 h to receive the percentage of the analyte (7) (n = 3).

	**Peak area**						
**Time [h]**	**(7)**	**Donepezil**	**A(Compound)/A(Standard)**	**A/A mean**	**Standard dev.**	**Var. [%]**	**Remaining compound [%]**
0	301656	550760	0.548				
0	304971	541354	0.563	0.553	0.009	1.6	100.0
0	276919	504757	0.549				
0.5	269119	546373	0.493				
0.5	299124	553606	0.540	0.512	0.025	4.9	92.5
0.5	277711	553250	0.502				
1	271521	558804	0.486				
1	289577	556327	0.521	0.509	0.020	3.9	91.9
1	288001	554652	0.519				
1.5	281559	558896	0.504				
1.5	273358	558015	0.490	0.496	0.007	1.4	89.7
1.5	272831	551269	0.495				
2	278445	559275	0.498				
2	274540	558381	0.492	0.493	0.005	1.0	89.0
2	270471	554073	0.488				
4	265306	561051	0.473				
4	271454	555761	0.488	0.480	0.008	1.7	86.7
4	266377	557935	0.477				
24	222895	563074	0.396				
24	239340	563741	0.425	0.414	0.016	3.7	74.8
24	236371	562308	0.420				

Table S15. Results of the incubation of (8) in human plasma over 24.00 h. Three samples were incubated over 24.00 h and measured at 230 nm with RP-UHPLC. The detected areas of (8) peaks were divided by the detected area of IS peaks (DOH). The average of the same time was converted to A(An)/A(IS). The calculated values were referred to the recovered (8) peak area at t = 0.00 h to receive the percentage of the analyte (8) (n = 3).

	Peak area						
**Time [h]**	**(8)**	**Donepezil**	**A(Compound)/A(Standard)**	**A/A mean**	**Standard dev.**	**Var. [%]**	**Remaining compound [%]**
0	667686	2121879	0.315				
0	715887	2096864	0.341	0.326	0.014	4.2	100.0
0	671060	2076550	0.323				
0.5	624308	1981362	0.315				
0.5	624404	1966112	0.318	0.316	0.001	0.4	96.8
0.5	631401	2003022	0.315				
1	588275	2050193	0.287				
1	646072	2002593	0.323	0.308	0.019	6.1	94.4
1	714429	2268318	0.315				
1.5	615489	1982637	0.310				
1.5	595086	2025934	0.294	0.303	0.009	2.8	92.9
1.5	609787	1994888	0.306				
2	598128	2012586	0.297				
2	592690	1994392	0.297	0.300	0.005	1.5	91.9
2	615395	2016659	0.305				
4	575972	2016614	0.286				
4	578824	2016570	0.287	0.286	0.001	0.3	87.6
4	565572	1983673	0.285				
24	568569	2038294	0.279				
24	561275	2023494	0.277	0.279	0.002	0.6	85.5
24	559268	1990867	0.281				

Table S16. Results of the incubation of (8) in rat plasma over 24.00 h. Three samples were incubated over 24.00 h and measured with MS. The detected areas of (8) peaks were divided by the detected area of IS peaks (DOH). The average of the same time was converted to A(An)/A(IS). The calculated values were referred to the recovered (8) peak area at t = 0.00 h to receive the percentage of the analyte (8) (n = 3).

	**Peak area**						
**Time [h]**	**(8)**	**Donepezil**	**A(Compound)/A(Standard)**	**A/A mean**	**Standard dev.**	**Var. [%]**	**Remaining compound [%]**
0	21116316	92107648	0.229				
0	20763694	87955776	0.236	0.234	0.005	1.9	100.0
0	19867850	83513680	0.238				
0.5	26253280	111927872	0.235				
0.5	13995031	61260912	0.228	0.234	0.005	2.3	99.9
0.5	37441996	156412720	0.239				
1	22178412	95966832	0.231				
1	20212432	87925648	0.230	0.232	0.002	1.0	98.9
1	29934212	127838616	0.234				
1.5	22474708	102665928	0.219				
1.5	19935296	85066304	0.234	0.227	0.008	3.4	96.9
1.5	18733366	81987912	0.228				
2	21937700	98083936	0.224				
2	18597198	79851888	0.233	0.227	0.005	2.4	96.7
2	25659998	114994800	0.223				
4	19213510	87821416	0.219				
4	12717193	58937116	0.216	0.217	0.002	0.8	92.5
4	9266172	42871660	0.216				
24	20720884	100390728	0.206				
24	17916136	86830384	0.206	0.212	0.010	4.5	90.4
24	17552068	78762424	0.223				

Table S17. Results of the incubation of (9) in human plasma over 24.00 h. Three samples were incubated over 24.00 h and measured at 300 nm with RP-UHPLC. The detected areas of (9) peaks were divided by the detected area of IS peaks (DOH). The average of the same time was converted to A(An)/A(IS). The calculated values were referred to the recovered (9) peak area at t = 0.00 h to receive the percentage of the analyte (9) (n = 3).

	Peak area						
**Time [h]**	**(9)**	**Donepezil**	**A(Compound)/A(Standard)**	**A/A mean**	**Standard dev.**	**Var. [%]**	**Remaining compound [%]**
0	227200	984298	0.231				
0	228135	988978	0.231	0.231	0.001	0.3	100.0
0	230408	992559	0.232				
0.5	224772	998349	0.225				
0.5	224495	991999	0.226	0.226	0.001	0.4	97.8
0.5	223210	983957	0.227				
1	219474	995291	0.221				
1	223332	991363	0.225	0.222	0.003	1.2	96.1
1	221741	1002843	0.221				
1.5	218292	996028	0.219				
1.5	223690	1001549	0.223	0.222	0.002	1.1	96.0
1.5	222557	996364	0.223				
2	222531	996028	0.223				
2	218694	1001549	0.218	0.221	0.003	1.2	95.4
2	219098	996364	0.220				
4	213090	998504	0.213				
4	219083	996614	0.220	0.219	0.006	2.6	94.9
4	221622	985758	0.225				
24	214792	997590	0.215				
24	205229	1007194	0.204	0.214	0.010	4.7	92.7
24	223299	996614	0.224				

Table S18. Results of the incubation of (9) in rat plasma over 24.00 h. Three samples were incubated over 24.00 h and measured at 300 nm with RP-UHPLC. The detected areas of (9) peaks were divided by the detected area of IS peaks (DOH). The average of the same time was converted to A(An)/A(IS). The calculated values were referred to the recovered (9) peak area at t = 0.00 h to receive the percentage of the analyte (9) (n = 3).

	Peak area						
**Time [h]**	**(9)**	**Donepezil**	**A(Compound)/A(Standard)**	**A/A mean**	**Standard dev.**	**Var. [%]**	**Remaining compound [%]**
0	242431	771840	0.314				
0	240121	782373	0.307	0.311	0.004	1.2	100.0
0	241518	775129	0.312				
0.5	228543	776198	0.294				
0.5	237542	769241	0.309	0.303	0.008	2.6	97.6
0.5	237634	773920	0.307				
1	228362	782392	0.292				
1	230202	780456	0.295	0.294	0.002	0.8	94.7
1	229707	774416	0.297				
1.5	222299	797741	0.279				
1.5	230026	770497	0.299	0.294	0.013	4.5	94.5
1.5	236351	777542	0.304				
2	228685	785871	0.291				
2	229287	773148	0.297	0.292	0.004	1.5	93.9
2	225415	782021	0.288				
4	225119	781986	0.288				
4	224862	775659	0.290	0.288	0.001	0.5	92.7
4	223001	776691	0.287				
24	157085	762196	0.206				
24	155488	764234	0.203	0.205	0.002	0.8	66.1
24	158491	766609	0.207				

Table S19. Results of the incubation of (10) in human plasma over 24.00 h. Three samples were incubated over 24.00 h and measured with the uV-detector of MS. The detected areas of (10) peaks were divided by the detected area of IS peaks (DOH). The average of the same time was converted to A(An)/A(IS). The calculated values were referred to the recovered (10) peak area at t = 0.00 h to receive the percentage of the analyte (10) (n = 3).

	Peak area						
**Time [h]**	**(10)**	**Donepezil**	**A(Compound)/A(Standard)**	**A/A mean**	**Standard dev.**	**Var. [%]**	**Remaining compound [%]**
0	54346	102636	0.530				
0	56021	109747	0.510	0.516	0.012	2.2	100.0
0	57183	112400	0.509				
0.5	57333	116661	0.491				
0.5	57004	112543	0.507	0.501	0.008	1.7	97.1
0.5	56604	112071	0.505				
1	54525	110366	0.494				
1	56745	112333	0.505	0.499	0.006	1.1	96.7
1	56524	113510	0.498				
1.5	55284	116541	0.474				
1.5	56231	114940	0.489	0.489	0.014	2.9	94.6
1.5	57075	113642	0.502				
2	57545	119780	0.480				
2	54478	117275	0.465	0.483	0.020	4.2	93.6
2	59693	118318	0.505				
4	55894	121908	0.458				
4	53235	110209	0.483	0.475	0.015	3.1	92.1
4	57433	118478	0.485				
24	48083	117421	0.409				
24	51668	123682	0.418	0.406	0.014	3.3	78.7
24	45065	115171	0.391				

Table S20. Results of the incubation of (10) in rat plasma over 24.00 h. Three samples were incubated over 24.00 h and measured with MS. The detected areas of (10) peaks were divided by the detected area of IS peaks (DOH). The average of the same time was converted to A(An)/A(IS). The calculated values were referred to the recovered (10) peak area at t = 0.00 h to receive the percentage of the analyte (10) (n = 3).

	Peak area						
**Time [h]**	**(10)**	**Donepezil**	**A(Compound)/A(Standard)**	**A/A mean**	**Standard dev.**	**Var. [%]**	**Remaining compound [%]**
0	12031054	49579548	0.243				
0	16251517	65025692	0.250	0.243	0.007	2.7	100.0
0	16485525	69646096	0.237				
0.5	18538576	82326952	0.225				
0.5	18463456	77191392	0.239	0.236	0.010	4.2	97.2
0.5	20449690	83632536	0.245				
1	11644319	52834672	0.220				
1	17327172	71364840	0.243	0.231	0.011	4.9	95.1
1	18784018	81537296	0.230				
1.5	20596586	90603160	0.227				
1.5	20867290	92951144	0.224	0.227	0.002	0.9	93.3
1.5	19643460	86057336	0.228				
2	17519630	78730856	0.223				
2	1963704	8744780	0.225	0.222	0.003	1.3	91.3
2	15369990	70204568	0.219				
4	18164766	83566816	0.217				
4	8639570	42467244	0.203	0.210	0.007	3.3	86.5
4	19641284	93627912	0.210				
24	16194805	96654352	0.168				
24	18785798	98422056	0.191	0.177	0.012	7.1	72.7
24	13299032	77519680	0.172				

Table S21. Results of the incubation of (11) in human plasma over 24.00 h. Three samples were incubated over 24.00 h and measured at 254 nm with RP-UHPLC. The detected areas of (11) peaks were divided by the detected area of IS peaks (DOH). The average of the same time was converted to A(An)/A(IS). The calculated values were referred to the recovered (11) peak area at t = 0.00 h to receive the percentage of the analyte (11) (n = 3).

	Peak area						
**Time [h]**	**(11)**	**Donepezil**	**A(Compound)/A(Standard)**	**A/A mean**	**Standard dev.**	**Var. [%]**	**Remaining compound [%]**
0	1535637	767822	2.000				
0	1654010	781849	2.116	2.059	0.058	2.8	100.0
0	1608069	780003	2.062				
0.5	1553694	788014	1.972				
0.5	1621590	780196	2.078	2.047	0.065	3.2	99.4
0.5	1614579	772281	2.091				
1	1538339	780653	1.971				
1	1610470	782173	2.059	2.032	0.053	2.6	98.7
1	1604552	776746	2.066				
1.5	1500571	785777	1.910				
1.5	1592822	770855	2.066	2.015	0.091	4.5	97.9
1.5	1594351	770339	2.070				
2	1490715	785295	1.898				
2	1578817	767471	2.057	2.011	0.098	4.9	97.7
2	1593689	767365	2.077				
4	1520039	769850	1.974				
4	1539575	756475	2.035	1.993	0.037	1.8	96.8
4	1504327	764087	1.969				
24	1511997	777516	1.945				
24	1502773	777852	1.932	1.966	0.049	2.5	95.5
24	1535389	759398	2.022				

Table S22. Results of the incubation of (11) in rat plasma over 24.00 h. Three samples were incubated over 24.00 h and measured at 254 nm with RP-UHPLC. The detected areas of (11) peaks were divided by the detected area of IS peaks (DOH). The average of the same time was converted to A(An)/A(IS). The calculated values were referred to the recovered (11) peak area at t = 0.00 h to receive the percentage of the analyte (11) (n = 3).

	Peak area						
**Time [h]**	**(11)**	**Donepezil**	**A(Compound)/A(Standard)**	**A/A mean**	**Standard dev.**	**Var. [%]**	**Remaining compound [%]**
0	1518073	609529	2.491				
0	1513350	597927	2.531	2.534	0.045	1.8	100.0
0	1580594	612399	2.581				
0.5	1467597	601331	2.441				
0.5	1462986	605138	2.418	2.493	0.111	4.5	98.4
0.5	1585940	605202	2.621				
1	1433177	606056	2.365				
1	1465222	623306	2.351	2.438	0.139	5.7	96.2
1	1610752	619977	2.598				
1.5	1565598	632726	2.474				
1.5	1442082	627630	2.298	2.423	0.109	4.5	95.6
1.5	1551044	621321	2.496				
2	1400401	568266	2.464				
2	1473279	639634	2.303	2.419	0.101	4.2	95.5
2	1583104	635754	2.490				
4	1429078	639971	2.233				
4	1469716	625267	2.351	2.357	0.127	5.4	93.0
4	1593448	640605	2.487				
24	1404952	774216	1.815				
24	1255867	723685	1.735	1.761	0.046	2.6	69.5
24	1318968	760578	1.734				

Table S23. Results of the incubation of (12) in human plasma over 24.00 h. Three samples were incubated over 24.00 h and measured at 254 nm with RP-UHPLC. The detected areas of (12) peaks were divided by the detected area of IS peaks (DOH). The average of the same time was converted to A(An)/A(IS). The calculated values were referred to the recovered (12) peak area at t = 0.00 h to receive the percentage of the analyte (12) (n = 3).

	Peak area						
**Time [h]**	**(12)**	**Donepezil**	**A(Compound)/A(Standard)**	**A/A mean**	**Standard dev.**	**Var. [%]**	**Remaining compound [%]**
0	286089	796217	0.359				
0	318458	797364	0.399	0.376	0.021	5.5	100.0
0	294176	794726	0.370				
0.5	287192	789368	0.364				
0.5	294192	783061	0.376	0.374	0.010	2.6	99.5
0.5	297442	775947	0.383				
1	290136	775173	0.374				
1	286456	772142	0.371	0.374	0.002	0.7	99.3
1	287791	765700	0.376				
1.5	286355	782795	0.366				
1.5	291583	774176	0.377	0.372	0.005	1.5	98.8
1.5	288121	773507	0.372				
2	279622	794730	0.352				
2	290239	786015	0.369	0.364	0.011	3.0	96.8
2	287971	774348	0.372				
4	262201	780779	0.336				
4	286870	775989	0.370	0.355	0.017	4.9	94.3
4	274807	764966	0.359				
24	222221	780009	0.285				
24	232491	770385	0.302	0.303	0.018	6.0	80.4
24	249070	776194	0.321				

Table S24. Results of the incubation of (12) in rat plasma over 24.00 h. Three samples were incubated over 24.00 h and measured at 254 nm with RP-UHPLC. The detected areas of (12) peaks were divided by the detected area of IS peaks (DOH). The average of the same time was converted to A(An)/A(IS). The calculated values were referred to the recovered (12) peak area at t = 0.00 h to receive the percentage of the analyte (12) (n = 3).

	Peak area						
**Time [h]**	**(12)**	**Donepezil**	**A(Compound)/A(Standard)**	**A/A mean**	**Standard dev.**	**Var. [%]**	**Remaining compound [%]**
0	281476	632573	0.445				
0	304679	629697	0.484	0.466	0.020	4.2	100.0
0	294101	627944	0.468				
0.5	267479	635067	0.421				
0.5	288233	627622	0.459	0.442	0.019	4.4	95.0
0.5	284392	637017	0.446				
1	261767	631108	0.415				
1	278088	638527	0.436	0.434	0.019	4.3	93.2
1	286920	635178	0.452				
1.5	260153	634787	0.410				
1.5	269793	630279	0.428	0.417	0.010	2.3	89.5
1.5	261821	634103	0.413				
2	257077	635241	0.405				
2	262997	631473	0.416	0.413	0.007	1.8	88.7
2	265233	634388	0.418				
4	237562	633140	0.375				
4	251473	631434	0.398	0.386	0.012	3.0	82.8
4	242728	633544	0.383				
24	173825	571894	0.304				
24	199440	644694	0.309	0.301	0.010	3.3	64.6
24	182679	630244	0.290				

Table S25. Results of the incubation of (13) in human plasma over 24.00 h. Three samples were incubated over 24.00 h and measured with the uV-detector of MS. The detected areas of (13) peaks were divided by the detected area of IS peaks (DOH). The average of the same time was converted to A(An)/A(IS). The calculated values were referred to the recovered (13) peak area at t = 0.00 h to receive the percentage of the analyte (13) (n = 3).

	**Peak area**						
**Time [h]**	**(13)**	**Donepezil**	**A(Compound)/A(Standard)**	**A/A mean**	**Standard dev.**	**Var. [%]**	**Remaining compound [%]**
0	21793138	81934936	0.266				
0	21010346	82737640	0.254	0.260	0.006	2.3	100.0
0	23064940	88746288	0.260				
0.5	3682101	15028836	0.245				
0.5	9768026	38698344	0.252	0.252	0.007	2.7	97.0
0.5	17835202	68900824	0.259				
1	21734922	86675800	0.251				
1	5609492	22911904	0.245	0.252	0.007	2.9	96.8
1	22335444	86133952	0.259				
1.5	12731594	50864644	0.250				
1.5	21627354	89755024	0.241	0.243	0.007	2.9	93.3
1.5	22896130	96842096	0.236				
2	19222306	81604280	0.236				
2	20765072	85753048	0.242	0.242	0.006	2.6	93.1
2	20614758	83129896	0.248				
4	21246038	89185304	0.238				
4	19933116	83608024	0.238	0.242	0.006	2.4	92.9
4	23329210	93994648	0.248				
24	21749920	92402648	0.235				
24	17279060	75305040	0.229	0.230	0.005	2.0	88.6
24	22115310	97750744	0.226				

Table S26. Results of the incubation of (13) in rat plasma over 24.00 h. Three samples were incubated over 24.00 h and measured with MS. The detected areas of (13) peaks were divided by the detected area of IS peaks (DOH). The average of the same time was converted to A(An)/A(IS). The calculated values were referred to the recovered (13) peak area at t = 0.00 h to receive the percentage of the analyte (13) (n = 3).

	Peak area						
**Time [h]**	**(13)**	**Donepezil**	**A(Compound)/A(Standard)**	**A/A mean**	**Standard dev.**	**Var. [%]**	**Remaining compound [%]**
0	15435392	65114580	0.237				
0	13184982	58422140	0.226	0.235	0.008	3.6	100.0
0	16250513	67084128	0.242				
0.5	18760516	84127312	0.223				
0.5	17766972	74751824	0.238	0.233	0.008	3.6	99.0
0.5	16922816	71278288	0.237				
1	17171378	75094320	0.229				
1	12742263	56035000	0.227	0.231	0.006	2.4	98.4
1	20089092	84548144	0.238				
1.5	18365728	83501064	0.220				
1.5	19504178	86124600	0.226	0.225	0.004	1.8	95.6
1.5	18850552	82846496	0.228				
2	18203932	84258448	0.216				
2	17281500	75227200	0.230	0.225	0.007	3.3	95.5
2	16208138	71156840	0.228				
4	18573360	83696056	0.222				
4	19182406	87461600	0.219	0.222	0.003	1.3	94.5
4	21365794	95000792	0.225				
24	17197028	84064648	0.205				
24	21473764	99728168	0.215	0.204	0.012	6.0	86.6
24	13254738	69443056	0.191				

Table S27. Results of the incubation of (14) in human plasma over 24.00 h. Three samples were incubated over 24.00 h and measured at 265 nm with RP-UHPLC. The detected areas of (14) peaks were divided by the detected area of IS peaks (DOH). The average of the same time was converted to A(An)/A(IS). The calculated values were referred to the recovered (14) peak area at t = 0.00 h to receive the percentage of the analyte (14) (n = 3).

	Peak area						
**Time [h]**	**(14)**	**Donepezil**	**A(Compound)/A(Standard)**	**A/A mean**	**Standard dev.**	**Var. [%]**	**Remaining compound [%]**
0	312958	1367975	0.229				
0	315957	1349983	0.234	0.233	0.004	1.9	100.0
0	324956	1367527	0.238				
0.5	313046	1366534	0.229				
0.5	310427	1341628	0.231	0.230	0.001	0.5	98.6
0.5	314536	1366399	0.230				
1	314014	1364662	0.230				
1	310579	1364662	0.228	0.228	0.002	0.7	97.8
1	311633	1370288	0.227				
1.5	305503	1377534	0.222				
1.5	311962	1360218	0.229	0.227	0.004	1.9	97.2
1.5	308718	1345601	0.229				
2	306117	1365688	0.224				
2	307870	1367790	0.225	0.225	0.001	0.6	96.5
2	311302	1371759	0.227				
4	300931	1397831	0.215				
4	285180	1406804	0.203	0.210	0.006	3.0	89.8
4	296734	1406010	0.211				
24	263745	1377534	0.191				
24	268202	1360218	0.197	0.197	0.005	2.6	84.3
24	271476	1345601	0.202				

Table S28. Results of the incubation of (14) in rat plasma over 24.00 h. Three samples were incubated over 24.00 h and measured at 265 nm with RP-UHPLC. The detected areas of (14) peaks were divided by the detected area of IS peaks (DOH). The average of the same time was converted to A(An)/A(IS). The calculated values were referred to the recovered (14) peak area at t = 0.00 h to receive the percentage of the analyte (14) (n = 3).

	Peak area						
**Time [h]**	**(14)**	**Donepezil**	**A(Compound)/A(Standard)**	**A/A mean**	**Standard dev.**	**Var. [%]**	**Remaining compound [%]**
0	329051	1128326	0.292				
0	326073	1128130	0.289	0.296	0.010	3.5	100.0
0	345664	1121437	0.308				
0.5	313952	1103925	0.284				
0.5	335502	1103925	0.304	0.293	0.010	3.4	98.8
0.5	323977	1115893	0.290				
1	314714	1161082	0.271				
1	324102	1154363	0.281	0.276	0.005	1.8	93.1
1	318128	1154060	0.276				
1.5	312802	1146504	0.273				
1.5	321826	1130483	0.285	0.276	0.008	2.9	93.0
1.5	310934	1155042	0.269				
2	313509	1182639	0.265				
2	329218	1157937	0.284	0.275	0.010	3.5	92.9
2	315623	1141688	0.276				
4	307403	1134829	0.271				
4	307674	1112939	0.276	0.268	0.010	3.8	90.5
4	286623	1116418	0.257				
24	333524	1238339	0.269				
24	331122	1217363	0.272	0.266	0.009	3.4	89.6
24	336577	1318244	0.255				

Table S29. Results of the incubation of (15) in human plasma over 24.00 h. Three samples were incubated over 24.00 h and measured with the uV-detector of MS. The detected areas of (15) peaks were divided by the detected area of IS peaks (DOH). The average of the same time was converted to A(An)/A(IS). The calculated values were referred to the recovered (15) peak area at t = 0.00 h to receive the percentage of the analyte (15) (n = 3).

	Peak area						
**Time [h]**	**(15)**	**Donepezil**	**A(Compound)/A(Standard)**	**A/A mean**	**Standard dev.**	**Var. [%]**	**Remaining compound [%]**
0	64056	114648	0.559				
0	63474	116790	0.543	0.562	0.020	3.6	100.0
0	66918	114699	0.583				
0.5	67909	118885	0.571				
0.5	64963	120565	0.539	0.554	0.016	2.9	98.6
0.5	64555	116919	0.552				
1	66622	120451	0.553				
1	66979	122724	0.546	0.551	0.004	0.8	98.0
1	67731	122375	0.553				
1.5	64063	121594	0.527				
1.5	67919	123256	0.551	0.542	0.013	2.4	96.4
1.5	66133	120943	0.547				
2	64473	119473	0.540				
2	65976	121927	0.541	0.538	0.005	0.8	95.7
2	64933	121924	0.533				
4	62112	118917	0.522				
4	63911	125084	0.511	0.518	0.006	1.2	92.2
4	63861	122413	0.522				
24	51191	117098	0.437				
24	55691	121130	0.460	0.450	0.012	2.6	80.1
24	58547	129396	0.452				

Table S30. Results of the incubation of (15) in rat plasma over 24.00 h. Three samples were incubated over 24.00 h and measured with MS. The detected areas of (15) peaks were divided by the detected area of IS peaks (DOH). The average of the same time was converted to A(An)/A(IS). The calculated values were referred to the recovered (15) peak area at t = 0.00 h to receive the percentage of the analyte (15) (n = 3).

	**Peak area**						
**Time [h]**	**(15)**	**Donepezil**	**A(Compound)/A(Standard)**	**A/A mean**	**Standard dev.**	**Var. [%]**	**Remaining compound [%]**
0	10523214	58240116	0.181				
0	12208307	71313248	0.171	0.175	0.005	3.0	100.0
0	13735796	79953608	0.172				
0.5	12358045	72926744	0.169				
0.5	13931195	80870656	0.172	0.171	0.001	0.8	97.8
0.5	12916484	75734824	0.171				
1	11637740	69493352	0.167				
1	12368943	73936160	0.167	0.168	0.001	0.7	96.3
1	11053154	65261276	0.169				
1.5	10884793	67997744	0.160				
1.5	11812279	68987120	0.171	0.166	0.006	3.4	95.2
1.5	13282895	79436880	0.167				
2	14818151	86320296	0.172				
2	14785874	88919208	0.166	0.166	0.006	3.8	94.9
2	15248911	95823904	0.159				
4	11657563	73516496	0.159				
4	12476769	76052728	0.164	0.162	0.003	2.1	93.0
4	12874127	78199200	0.165				
24	11015492	83324848	0.132				
24	9719038	70213032	0.138	0.137	0.004	2.9	78.3
24	16814686	120427328	0.140				

Table S31. Results of the incubation of (16) in human plasma over 24.00 h. Three samples were incubated over 24.00 h and measured at 270 nm with RP-UHPLC. The detected areas of (16) peaks were divided by the detected area of IS peaks (DOH). The average of the same time was converted to A(An)/A(IS). The calculated values referred to the recovered (16) peak area at t = 0.00 h to receive the percentage of the analyte (1) (n = 3).

	Peak area						
**Time [h]**	**(16)**	**Donepezil**	**A(Compound)/A(Standard)**	**A/A mean**	**Standard dev.**	**Var. [%]**	**Remaining compound [%]**
0	6447495	587223	10.980				
0	6430499	591555	10.871	10.729	0.344	3.2	100.0
0	6133569	593343	10.337				
0.5	5807902	568955	10.208				
0.5	6356739	611652	10.393	10.467	0.303	2.9	97.6
0.5	6315353	584711	10.801				
1	5807902	594971	9.762				
1	6236090	583936	10.679	10.241	0.460	4.5	95.4
1	6302164	613012	10.281				
1.5	5764503	598850	9.626				
1.5	6237804	587114	10.625	10.223	0.527	5.2	95.3
1.5	6089129	584494	10.418				
2	5683296	586757	9.686				
2	6182312	590635	10.467	10.127	0.400	4.0	94.4
2	6001523	586773	10.228				
4	5401422	598011	9.032				
4	5612768	565428	9.927	9.439	0.453	4.8	88.0
4	5675892	606565	9.357				
24	4024152	583890	6.892				
24	4562704	597039	7.642	7.128	0.446	6.3	66.4
24	4022998	587442	6.848				

Table S32. Results of the incubation of (16) in rat plasma over 24.00 h. Three samples were incubated over 24.00 h and measured at 270 nm with RP-UHPLC. The detected areas of (16) peaks were divided by the detected area of IS peaks (DOH). The average of the same time was converted to A(An)/A(IS). The calculated values referred to the recovered (16) peak area at t = 0.00 h to receive the percentage of the analyte (1) (n = 3).

	Peak area						
**Time [h]**	**(16)**	**Donepezil**	**A(Compound)/A(Standard)**	**A/A mean**	**Standard dev.**	**Var. [%]**	**Remaining compound [%]**
0	1276881	544000	2.347				
0	2627851	1126571	2.333	2.341	0.008	0.3	100.0
0	2454100	1047248	2.343				
0.5	1478221	637800	2.318				
0.5	1513209	672956	2.249	2.318	0.070	3.0	99.0
0.5	1836997	769106	2.388				
1	2454100	1047248	2.343				
1	2237856	1012263	2.211	2.234	0.100	4.5	95.4
1	2314523	1078316	2.146				
1.5	1578470	774273	2.039				
1.5	1106434	510175	2.169	2.118	0.070	3.3	90.5
1.5	2314523	1078316	2.146				
2	1782012	948386	1.879				
2	1547371	875688	1.767	1.803	0.066	3.7	77.0
2	1500524	851110	1.763				
4	1531553	901937	1.698				
4	1500524	851110	1.763	1.725	0.034	2.0	73.7
4	1432325	836274	1.713				
24	1079621	1350387	0.799				
24	865056	1088572	0.795	0.833	0.063	7.6	35.6
24	1121812	1237985	0.906				

Table S33. Results of the incubation of (17) in human plasma over 24.00 h. Three samples were incubated over 24.00 h and measured at 254 nm with RP-UHPLC. The detected areas of (17) peaks were divided by the detected area of IS peaks (DOH). The average of the same time was converted to A(An)/A(IS). The calculated values were referred to the recovered (17) peak area at t = 0.00 h to receive the percentage of the analyte (17) (n = 3).

	Peak area						
**Time [h]**	**(17)**	**Donepezil**	**A(Compound)/A(Standard)**	**A/A mean**	**Standard dev.**	**Var. [%]**	**Remaining compound [%]**
0	339727	781666	0.435				
0	368165	778672	0.473	0.450	0.020	4.5	100.0
0	342065	773781	0.442				
0.5	341096	772991	0.441				
0.5	349357	776338	0.450	0.442	0.007	1.6	98.3
0.5	336323	771779	0.436				
1	334553	774314	0.432				
1	346246	771331	0.449	0.432	0.016	3.8	96.1
1	323453	776903	0.416				
1.5	332177	784524	0.423				
1.5	339615	774276	0.439	0.432	0.008	1.8	95.9
1.5	335952	776596	0.433				
2	320193	773549	0.414				
2	338815	776669	0.436	0.429	0.013	3.0	95.4
2	339058	776476	0.437				
4	327261	784747	0.417				
4	329334	775997	0.424	0.415	0.011	2.6	92.2
4	314021	779382	0.403				
24	304759	790015	0.386				
24	294678	761208	0.387	0.397	0.018	4.6	88.3
24	317904	760030	0.418				

Table S34. Results of the incubation of (17) in rat plasma over 24.00 h. Three samples were incubated over 24.00 h and measured at 254 nm with RP-UHPLC. The detected areas of (17) peaks were divided by the detected area of IS peaks (DOH). The average of the same time was converted to A(An)/A(IS). The calculated values were referred to the recovered (17) peak area at t = 0.00 h to receive the percentage of the analyte (17) (n = 3).

	Peak area						
**Time [h]**	**(17)**	**Donepezil**	**A(Compound)/A(Standard)**	**A/A mean**	**Standard dev.**	**Var. [%]**	**Remaining compound [%]**
0	135401	345025	0.392				
0	131931	353089	0.374	0.388	0.012	3.2	100.0
0	136277	343135	0.397				
0.5	168254	459216	0.366				
0.5	151757	383790	0.395	0.382	0.015	3.8	98.4
0.5	138799	362345	0.383				
1	153251	425010	0.361				
1	179106	471176	0.380	0.373	0.011	3.0	96.3
1	175727	463365	0.379				
1.5	182017	479168	0.380				
1.5	167946	443790	0.378	0.372	0.012	3.1	96.1
1.5	192964	537368	0.359				
2	175876	493301	0.357				
2	208884	536859	0.389	0.372	0.016	4.4	95.9
2	197599	534029	0.370				
4	196671	553272	0.355				
4	223117	612692	0.364	0.364	0.008	2.3	93.9
4	229300	616168	0.372				
24	209123	730334	0.286				
24	218562	719985	0.304	0.296	0.009	3.0	76.4
24	196446	657126	0.299				

Table S35. Results of the incubation of (18) in human plasma over 24.00 h. Three samples were incubated over 24.00 h and measured at 300 nm with RP-UHPLC. The detected areas of (18) peaks were divided by the detected area of IS peaks (DOH). The average of the same time was converted to A(An)/A(IS). The calculated values were referred to the recovered (18) peak area at t = 0.00 h to receive the percentage of the analyte (18) (n = 3).

	Peak area						
**Time [h]**	**(18)**	**Donepezil**	**A(Compound)/A(Standard)**	**A/A mean**	**Standard dev.**	**Var. [%]**	**Remaining compound [%]**
0	223457	986000	0.227				
0	225104	990334	0.227	0.228	0.002	0.7	100.0
0	224302	977167	0.230				
0.5	224885	985839	0.228				
0.5	220171	975156	0.226	0.225	0.004	1.6	98.8
0.5	222166	1004274	0.221				
1	218457	993704	0.220				
1	222022	994786	0.223	0.222	0.002	0.9	97.5
1	222099	995235	0.223				
1.5	218296	1004131	0.217				
1.5	219835	979418	0.224	0.221	0.004	1.6	97.0
1.5	219281	991989	0.221				
2	218307	1004131	0.217				
2	220214	979418	0.225	0.218	0.006	2.7	95.9
2	211264	991989	0.213				
4	208069	978700	0.213				
4	208272	984760	0.211	0.212	0.001	0.3	93.0
4	223903	1059053	0.211				
24	196862	986000	0.200				
24	202488	990334	0.204	0.203	0.003	1.4	89.1
24	200097	977167	0.205				

Table S36. Results of the incubation of (18) in rat plasma over 24.00 h. Three samples were incubated over 24.00 h and measured at 300 nm with RP-UHPLC. The detected areas of (18) peaks were divided by the detected area of IS peaks (DOH). The average of the same time was converted to A(An)/A(IS). The calculated values were referred to the recovered (18) peak area at t = 0.00 h to receive the percentage of the analyte (18) (n = 3).

	Peak area						
**Time [h]**	**(18)**	**Donepezil**	**A(Compound)/A(Standard)**	**A/A mean**	**Standard dev.**	**Var. [%]**	**Remaining compound [%]**
0	233525	721398	0.324				
0	234664	738209	0.318	0.320	0.004	1.1	100.0
0	234664	739698	0.317				
0.5	222709	727043	0.306				
0.5	230995	739329	0.312	0.312	0.006	1.9	97.7
0.5	232625	730380	0.318				
1	225268	725170	0.311				
1	223885	734352	0.305	0.310	0.005	1.6	97.1
1	225730	716455	0.315				
1.5	224037	737696	0.304				
1.5	222644	735801	0.303	0.306	0.005	1.5	95.7
1.5	228741	735457	0.311				
2	219472	737129	0.298				
2	219130	733989	0.299	0.296	0.004	1.3	92.6
2	218857	750209	0.292				
4	217969	749384	0.291				
4	218197	752994	0.290	0.292	0.004	1.3	91.5
4	217066	731460	0.297				
24	150136	725093	0.207				
24	140283	741204	0.189	0.206	0.016	7.9	64.4
24	161719	729963	0.222				

Table S37. Results of the incubation of (19) in human plasma over 24.00 h. Three samples were incubated over 24.00 h and measured at 254 nm with RP-UHPLC. The detected areas of (19) peaks were divided by the detected area of IS peaks (DOH). The average of the same time was converted to A(An)/A(IS). The calculated values were referred to the recovered (19) peak area at t = 0.00 h to receive the percentage of the analyte (19) (n = 3).

	**Peak area**						
**Time [h]**	**(19)**	**Donepezil**	**A(Compound)/A(Standard)**	**A/A mean**	**Standard dev.**	**Var. [%]**	**Remaining compound [%]**
0	290838	789319	0.368				
0	293901	793525	0.370	0.373	0.006	1.6	100.0
0	297418	783088	0.380				
0.5	293699	787702	0.373				
0.5	293367	777299	0.377	0.370	0.008	2.3	99.3
0.5	280946	778084	0.361				
1	286010	767834	0.372				
1	280511	767416	0.366	0.370	0.004	1.0	99.1
1	286362	772768	0.371				
1.5	286602	777121	0.369				
1.5	286982	777949	0.369	0.366	0.006	1.6	98.0
1.5	283237	789108	0.359				
2	278393	789443	0.353				
2	280388	777049	0.361	0.362	0.010	2.9	97.2
2	288080	771608	0.373				
4	272502	784428	0.347				
4	269808	764774	0.353	0.352	0.004	1.2	94.4
4	272497	766888	0.355				
24	279068	807362	0.346				
24	267603	779944	0.343	0.343	0.002	0.6	92.1
24	263418	771456	0.341				

Table S38. Results of the incubation of (19) in rat plasma over 24.00 h. Three samples were incubated over 24.00 h and measured at 254 nm with RP-UHPLC. The detected areas of (19) peaks were divided by the detected area of IS peaks (DOH). The average of the same time was converted to A(An)/A(IS). The calculated values were referred to the recovered (19) peak area at t = 0.00 h to receive the percentage of the analyte (19) (n = 3).

	Peak area						
**Time [h]**	**(19)**	**Donepezil**	**A(Compound)/A(Standard)**	**A/A mean**	**Standard dev.**	**Var. [%]**	**Remaining compound [%]**
0	267855	600583	0.446				
0	272191	598955	0.454	0.452	0.005	1.1	100.0
0	272402	598803	0.455				
0.5	268188	603080	0.445				
0.5	266030	595366	0.447	0.444	0.003	0.6	98.4
0.5	264363	598590	0.442				
1	263518	602782	0.437				
1	264692	602587	0.439	0.438	0.001	0.2	97.0
1	263862	601469	0.439				
1.5	254422	596783	0.426				
1.5	261996	591790	0.443	0.431	0.010	2.4	95.4
1.5	256594	605587	0.424				
2	253998	611074	0.416				
2	255974	601304	0.426	0.420	0.005	1.2	93.0
2	254024	605161	0.420				
4	250274	607875	0.412				
4	252794	607875	0.416	0.414	0.002	0.5	91.7
4	250684	603631	0.415				
24	243036	605003	0.402				
24	242608	606421	0.400	0.401	0.001	0.2	88.8
24	243118	606100	0.401				

Table S39. Results of the incubation of (20) in human plasma over 24.00 h. Three samples were incubated over 24.00 h and measured with MS. The detected areas of (20) peaks were divided by the detected area of IS peaks (DOH). The average of the same time was converted to A(An)/A(IS). The calculated values were referred to the recovered (20) peak area at t = 0.00 h to receive the percentage of the analyte (20) (n = 3).

	**Peak area**						
**Time [h]**	**(20)**	**Donepezil**	**A(Compound)/A(Standard)**	**A/A mean**	**Standard dev.**	**Var. [%]**	**Remaining compound [%]**
0	342310	602729	0.568				
0	342685	597784	0.573	0.570	0.003	0.5	100.0
0	338528	595647	0.568				
0.5	324553	607768	0.534				
0.5	339858	597072	0.569	0.553	0.018	3.2	97.1
0.5	336145	604566	0.556				
1	316718	598850	0.529				
1	332814	601871	0.553	0.542	0.012	2.3	95.2
1	330809	606679	0.545				
1.5	323535	593112	0.545				
1.5	312821	585866	0.534	0.540	0.006	1.1	94.8
1.5	320225	591242	0.542				
2	318847	595501	0.535				
2	318451	584141	0.545	0.538	0.006	1.1	94.5
2	312576	584803	0.534				
4	311008	601370	0.517				
4	313189	597359	0.524	0.518	0.006	1.2	90.8
4	306316	598708	0.512				
24	211000	571171	0.369				
24	237610	587275	0.405	0.392	0.019	5.0	68.8
24	237659	592014	0.401				

Table S40. Results of the incubation of (20) in rat plasma over 24.00 h. Three samples were incubated over 24.00 h and measured with MS. The detected areas of (20) peaks were divided by the detected area of IS peaks (DOH). The average of the same time was converted to A(An)/A(IS). The calculated values were referred to the recovered (20) peak area at t = 0.00 h to receive the percentage of the analyte (20) (n = 3).

	**Peak area**						
**Time [h]**	**(20)**	**Donepezil**	**A(Compound)/A(Standard)**	**A/A mean**	**Standard dev.**	**Var. [%]**	**Remaining compound [%]**
0	33352490	112649232	0.296				
0	31396178	109953664	0.286	0.287	0.009	3.0	100.0
0	27521996	98623592	0.279				
0.5	35131280	130086304	0.270				
0.5	31958084	113352808	0.282	0.276	0.006	2.2	96.3
0.5	30651818	110619224	0.277				
1	28237236	108542784	0.260				
1	47699936	162658736	0.293	0.274	0.017	6.2	95.6
1	20662530	76625480	0.270				
1.5	46011444	162305648	0.283				
1.5	25050590	94011144	0.266	0.270	0.012	4.4	94.2
1.5	18627222	71393680	0.261				
2	18888502	78739144	0.240				
2	25108364	97849208	0.257	0.249	0.009	3.5	87.0
2	17612968	69928472	0.252				
4	18793472	91302376	0.206				
4	19583212	86140160	0.227	0.222	0.014	6.2	77.2
4	27286198	117799432	0.232				
24	8678500	82557888	0.105				
24	9497357	86806032	0.109	0.111	0.007	6.5	38.8
24	17902550	150073024	0.119				

Table S41. Results of the incubation of (21) in human plasma over 24.00 h. Three samples were incubated over 24.00 h and measured with MS. The detected areas of (21) peaks were divided by the detected area of IS peaks (DOH). The average of the same time was converted to A(An)/A(IS). The calculated values were referred to the recovered (21) peak area at t = 0.00 h to receive the percentage of the analyte (21) (n = 3).

	Peak area						
**Time [h]**	**(21)**	**Donepezil**	**A(Compound)/A(Standard)**	**A/A mean**	**Standard dev.**	**Var. [%]**	**Remaining compound [%]**
0	25397604	77597200	0.327				
0	20151388	60394884	0.334	0.331	0.003	1.0	100.0
0	26708072	80350216	0.332				
0.5	24117914	74489392	0.324				
0.5	22049562	67697080	0.326	0.330	0.009	2.7	99.6
0.5	23994210	70544136	0.340				
1	29809796	90861080	0.328				
1	24251438	74419048	0.326	0.328	0.002	0.5	98.9
1	30670772	93282048	0.329				
1.5	21739032	71614000	0.304				
1.5	25550050	81179024	0.315	0.312	0.008	2.6	94.4
1.5	26263380	82320408	0.319				
2	23660824	75380264	0.314				
2	24156986	80882616	0.299	0.308	0.008	2.7	93.1
2	26243662	84083976	0.312				
4	25960590	83532264	0.311				
4	25240776	83074136	0.304	0.307	0.003	1.1	92.8
4	24949104	81217352	0.307				
24	22050454	77221784	0.286				
24	22694012	83627720	0.271	0.277	0.008	2.7	83.7
24	20903806	76232792	0.274				

Table S42. Results of the incubation of (21) in rat plasma over 24.00 h. Three samples were incubated over 24.00 h and measured with MS. The detected areas of (21) peaks were divided by the detected area of IS peaks (DOH). The average of the same time was converted to A(An)/A(IS). The calculated values were referred to the recovered (21) peak area at t = 0.00 h to receive the percentage of the analyte (21) (n = 3).

	**Peak area**						
**Time [h]**	**(21)**	**Donepezil**	**A(Compound)/A(Standard)**	**A/A mean**	**Standard dev.**	**Var. [%]**	**Remaining compound [%]**
0	15933347	63369504	0.251				
0	20465000	79778216	0.257	0.260	0.010	4.0	100.0
0	17951334	66147548	0.271				
0.5	16788468	66766392	0.251				
0.5	22395562	86716936	0.258	0.257	0.005	2.1	99.0
0.5	18367944	70140936	0.262				
1	17214158	67300616	0.256				
1	16113917	65030936	0.248	0.256	0.009	3.4	98.7
1	18535124	69832592	0.265				
1.5	15584572	64792704	0.241				
1.5	15833989	65763228	0.241	0.248	0.012	4.9	95.3
1.5	21304972	81458120	0.262				
2	18868844	78224096	0.241				
2	19870276	79486104	0.250	0.244	0.005	2.2	93.9
2	10473697	43591004	0.240				
4	16407098	77815120	0.211				
4	17474898	78510912	0.223	0.219	0.007	3.4	84.5
4	18566714	82602528	0.225				
24	21303306	204852448	0.104				
24	22947118	212647568	0.108	0.106	0.002	2.0	41.0
24	22121534	206284416	0.107				

Table S43. Results of the incubation of (22) in human plasma over 24.00 h. Three samples were incubated over 24.00 h and measured with MS. The detected areas of (22) peaks were divided by the detected area of IS peaks (DOH). The average of the same time was converted to A(An)/A(IS). The calculated values were referred to the recovered (22) peak area at t = 0.00 h to receive the percentage of the analyte (22) (n = 3).

	Peak area						
**Time [h]**	**(22)**	**Donepezil**	**A(Compound)/A(Standard)**	**A/A mean**	**Standard dev.**	**Var. [%]**	**Remaining compound [%]**
0	87404	116098	0.753				
0	86954	115515	0.753	0.748	0.008	1.0	100.0
0	84510	114246	0.740				
0.5	86502	122269	0.707				
0.5	85630	121815	0.703	0.716	0.018	2.5	95.6
0.5	90593	123039	0.736				
1	83644	119165	0.702				
1	88183	123640	0.713	0.714	0.012	1.7	95.3
1	87274	120259	0.726				
1.5	88205	123708	0.713				
1.5	87736	128595	0.682	0.706	0.022	3.1	94.4
1.5	87840	121315	0.724				
2	76782	111521	0.688				
2	76481	113755	0.672	0.667	0.025	3.7	89.1
2	78314	122465	0.639				
4	81287	124115	0.655				
4	81971	124699	0.657	0.659	0.005	0.8	88.0
4	77376	116430	0.665				
24	74930	121274	0.618				
24	73585	118336	0.622	0.624	0.007	1.1	83.3
24	80713	127843	0.631				

Table S44. Results of the incubation of (22) in rat plasma over 24.00 h. Three samples were incubated over 24.00 h and measured with MS. The detected areas of (22) peaks were divided by the detected area of IS peaks (DOH). The average of the same time was converted to A(An)/A(IS). The calculated values were referred to the recovered (22) peak area at t = 0.00 h to receive the percentage of the analyte (22) (n = 3).

	**Peak area**						
**Time [h]**	**(22)**	**Donepezil**	**A(Compound)/A(Standard)**	**A/A mean**	**Standard dev.**	**Var. [%]**	**Remaining compound [%]**
0	13180268	79537056	0.166				
0	13793858	77199080	0.179	0.172	0.006	3.8	100.0
0	13263236	77329048	0.172				
0.5	12292884	76197064	0.161				
0.5	14929334	84015392	0.178	0.170	0.008	4.8	98.7
0.5	13866409	81540448	0.170				
1	11866979	73936256	0.161				
1	11765297	67660312	0.174	0.169	0.007	4.3	98.2
1	12823100	74440800	0.172				
1.5	11013102	67982400	0.162				
1.5	13613660	77786656	0.175	0.168	0.007	4.0	97.5
1.5	12662964	76192264	0.166				
2	12359585	78156592	0.158				
2	11259950	67406904	0.167	0.163	0.004	2.7	94.5
2	12694268	78193848	0.162				
4	13601091	86038576	0.158				
4	13769824	86345424	0.159	0.161	0.004	2.7	93.8
4	12976723	78035600	0.166				
24	20033838	215019728	0.093				
24	21070652	207664368	0.101	0.098	0.004	4.3	56.7
24	21999278	224367072	0.098				

Table S45. Results of the incubation of (23) in human plasma over 24.00 h. Three samples were incubated over 24.00 h and measured at 254 nm with RP-UHPLC. The detected areas of (23) peaks were divided by the detected area of IS peaks (DOH). The average of the same time was converted to A(An)/A(IS). The calculated values referred to the recovered (23) peak area at t = 0.00 h to receive the percentage of the analyte (1) (n = 3).

	Peak area						
**Time [h]**	**(23)**	**Donepezil**	**A(Compound)/A(Standard)**	**A/A mean**	**Standard dev.**	**Var. [%]**	**Remaining compound [%]**
0	1360759	584065	2.330				
0	1359624	582872	2.333	2.310	0.037	1.6	100.0
0	1325718	584753	2.267				
0.5	1211006	615039	1.969				
0.5	1268180	599213	2.116	2.060	0.080	3.9	89.2
0.5	1259626	601246	2.095				
1	1192910	616992	1.933				
1	1264948	602806	2.098	2.037	0.090	4.4	88.2
1	1254196	603223	2.079				
1.5	1193057	599701	1.989				
1.5	1185134	582031	2.036	2.029	0.036	1.8	87.8
1.5	1188914	576834	2.061				
2	1187331	603311	1.968				
2	1178193	587727	2.005	2.001	0.031	1.5	86.6
2	1188665	585846	2.029				
4	1177585	609540	1.932				
4	1175180	599781	1.959	1.965	0.036	1.8	85.1
4	1191937	595119	2.003				
24	984408	600783	1.639				
24	849985	585368	1.452	1.569	0.102	6.5	67.9
24	974860	603049	1.617				

Table S46. Results of the incubation of (23) in rat plasma over 24.00 h. Three samples were incubated over 24.00 h and measured with MS. The detected areas of (23) peaks were divided by the detected area of IS peaks (DOH). The average of the same time was converted to A(An)/A(IS). The calculated values were referred to the recovered (23) peak area at t = 0.00 h to receive the percentage of the analyte (23) (n = 3).

	Peak area						
**Time [h]**	**(23)**	**Donepezil**	**A(Compound)/A(Standard)**	**A/A mean**	**Standard dev.**	**Var. [%]**	**Remaining compound [%]**
0	27963944	156800640	0.178				
0	4746735	28333624	0.168	0.171	0.006	3.5	100.0
0	16485724	97824496	0.169				
0.5	15762470	96661368	0.163				
0.5	14171034	87081904	0.163	0.167	0.006	3.8	97.2
0.5	24611320	141475504	0.174				
1	17732706	115163208	0.154				
1	19603142	117967712	0.166	0.164	0.009	5.3	95.5
1	27706708	162066848	0.171				
1.5	11273027	71739992	0.157				
1.5	17363064	102570584	0.169	0.163	0.006	3.7	95.1
1.5	27033946	166165792	0.163				
2	16777520	109141400	0.154				
2	16756938	103223736	0.162	0.160	0.005	3.2	93.1
2	24029996	147781072	0.163				
4	15425822	103003616	0.150				
4	17087858	105636296	0.162	0.158	0.007	4.5	92.1
4	16006161	98599384	0.162				
24	6176267	49999944	0.124				
24	11561980	86361144	0.134	0.128	0.005	4.1	74.7
24	10540411	83217952	0.127				

Table S47. Results of the incubation of (24) in human plasma over 24.00 h. Three samples were incubated over 24.00 h and measured at 260 nm with RP-UHPLC. The detected areas of (24) peaks were divided by the detected area of IS peaks (DOH). The average of the same time was converted to A(An)/A(IS). The calculated values were referred to the recovered (24) peak area at t = 0.00 h to receive the percentage of the analyte (24) (n = 3).

	Peak area						
**Time [h]**	**(24)**	**Donepezil**	**A(Compound)/A(Standard)**	**A/A mean**	**Standard dev.**	**Var. [%]**	**Remaining compound [%]**
0	250360	1121804	0.223				
0	270958	1138422	0.238	0.232	0.008	3.5	100.0
0	262809	1112910	0.236				
0.5	246462	1135980	0.217				
0.5	247707	1138114	0.218	0.223	0.010	4.3	95.8
0.5	263676	1127944	0.234				
1	240486	1125961	0.214				
1	253108	1150129	0.220	0.219	0.004	2.0	94.0
1	240486	1082520	0.222				
1.5	245771	1140131	0.216				
1.5	247551	1131570	0.219	0.218	0.002	0.8	93.6
1.5	247054	1131853	0.218				
2	240117	1105492	0.217				
2	240960	1100105	0.219	0.216	0.004	1.8	92.9
2	243062	1148639	0.212				
4	247146	1138102	0.217				
4	245599	1132873	0.217	0.216	0.002	0.9	92.8
4	247363	1158864	0.213				
24	248358	1138102	0.218				
24	237940	1132873	0.210	0.212	0.005	2.4	91.3
24	241821	1158864	0.209				

Table S48. Results of the incubation of (24) in rat plasma over 24.00 h. Three samples were incubated over 24.00 h and measured with MS. The detected areas of (24) peaks were divided by the detected area of IS peaks (DOH). The average of the same time was converted to A(An)/A(IS). The calculated values were referred to the recovered (24) peak area at t = 0.00 h to receive the percentage of the analyte (24) (n = 3).

	Peak area						
**Time [h]**	**(24)**	**Donepezil**	**A(Compound)/A(Standard)**	**A/A mean**	**Standard dev.**	**Var. [%]**	**Remaining compound [%]**
0	61611008	93908928	0.656				
0	24264314	36951288	0.657	0.652	0.008	1.2	100.0
0	25457950	39636584	0.642				
0.5	30031410	45866256	0.655				
0.5	35441452	53942464	0.657	0.644	0.020	3.2	98.8
0.5	32317938	52078796	0.621				
1	46463908	73600056	0.631				
1	28974018	47100284	0.615	0.631	0.016	2.5	96.8
1	14182182	21944126	0.646				
1.5	20979350	33013592	0.635				
1.5	14736256	24572370	0.600	0.620	0.019	3.0	95.2
1.5	17651218	28191784	0.626				
2	36415292	59699524	0.610				
2	69601984	121846160	0.571	0.590	0.019	3.3	90.6
2	36832736	62453488	0.590				
4	36027688	60522304	0.595				
4	53352132	97402960	0.548	0.577	0.026	4.4	88.5
4	30231722	51409860	0.588				
24	13373340	27558870	0.485				
24	43162060	89642536	0.481	0.471	0.022	4.7	72.2
24	14284867	32110100	0.445				

Table S49. Results of the incubation of (25) in human plasma over 24.00 h. Three samples were incubated over 24.00 h and measured with MS. The detected areas of (25) peaks were divided by the detected area of IS peaks (DOH). The average of the same time was converted to A(An)/A(IS). The calculated values were referred to the recovered (25) peak area at t = 0.00 h to receive the percentage of the analyte (25) (n = 3).

	Peak area						
**Time [h]**	**(25)**	**Donepezil**	**A(Compound)/A(Standard)**	**A/A mean**	**Standard dev.**	**Var. [%]**	**Remaining compound [%]**
0	69420528	86643544	0.801				
0	45333308	57176280	0.793	0.792	0.010	1.2	100.0
0	44100904	56403132	0.782				
0.5	57344692	76646144	0.748				
0.5	36769260	47182420	0.779	0.762	0.016	2.1	96.2
0.5	48461072	63980064	0.757				
1	58890892	81923616	0.719				
1	18804004	24350272	0.772	0.747	0.027	3.6	94.3
1	16450568	21979068	0.748				
1.5	41878100	58874172	0.711				
1.5	20197728	26751184	0.755	0.740	0.025	3.3	93.4
1.5	11717529	15564084	0.753				
2	53475644	74298936	0.720				
2	56473836	75137832	0.752	0.735	0.016	2.2	92.8
2	52120748	71117504	0.733				
4	45717056	64177424	0.712				
4	54008868	73273288	0.737	0.722	0.013	1.9	91.1
4	52914412	73908680	0.716				
24	50345472	69995952	0.719				
24	53410456	75888008	0.704	0.716	0.011	1.5	90.4
24	49964524	68943488	0.725				

Table S50. Results of the incubation of (25) in rat plasma over 24.00 h. Three samples were incubated over 24.00 h and measured with MS. The detected areas of (25) peaks were divided by the detected area of IS peaks (DOH). The average of the same time was converted to A(An)/A(IS). The calculated values were referred to the recovered (25) peak area at t = 0.00 h to receive the percentage of the analyte (25) (n = 3).

	Peak area						
**Time [h]**	**(25)**	**Donepezil**	**A(Compound)/A(Standard)**	**A/A mean**	**Standard dev.**	**Var. [%]**	**Remaining compound [%]**
0	39651820	67517144	0.587				
0	39183756	69576904	0.563	0.579	0.014	2.4	100.0
0	40241988	68586600	0.587				
0.5	49010632	85600000	0.573				
0.5	44355732	76875536	0.577	0.578	0.005	0.9	99.8
0.5	42142260	72234544	0.583				
1	40811884	73219376	0.557				
1	45528468	79221944	0.575	0.572	0.013	2.2	98.7
1	38627556	66319572	0.582				
1.5	40031464	73453584	0.545				
1.5	40371148	72806112	0.555	0.560	0.018	3.3	96.7
1.5	40016928	68953760	0.580				
2	43391428	77680176	0.559				
2	43076252	76341424	0.564	0.555	0.012	2.2	95.8
2	42086192	77780648	0.541				
4	39122816	76795584	0.509				
4	44178416	80457848	0.549	0.522	0.024	4.5	90.1
4	31222550	61608668	0.507				
24	80118008	163006320	0.492				
24	104059976	197506336	0.527	0.519	0.024	4.7	89.6
24	107156208	199077488	0.538				

Table S51. Results of the incubation of (26) in human plasma over 24.00 h. Three samples were incubated over 24.00 h and measured with MS. The detected areas of (26) peaks were divided by the detected area of IS peaks (DOH). The average of the same time was converted to A(An)/A(IS). The calculated values were referred to the recovered (26) peak area at t = 0.00 h to receive the percentage of the analyte (26) (n = 3).

	Peak area						
**Time [h]**	**(26)**	**Donepezil**	**A(Compound)/A(Standard)**	**A/A mean**	**Standard dev.**	**Var. [%]**	**Remaining compound [%]**
0	10451876	18397430	0.568				
0	41716248	70040288	0.596	0.584	0.014	2.4	100.0
0	46549484	79157864	0.588				
0.5	32867058	59047076	0.557				
0.5	18681610	31640644	0.590	0.577	0.018	3.1	98.8
0.5	48185512	82429976	0.585				
1	37758068	68550048	0.551				
1	22165822	37618532	0.589	0.572	0.019	3.4	97.9
1	43123956	75054688	0.575				
1.5	41979796	76037072	0.552				
1.5	42987220	72525248	0.593	0.570	0.021	3.6	97.7
1.5	48564328	85800112	0.566				
2	36203656	65795124	0.550				
2	38246084	67142512	0.570	0.557	0.011	2.0	95.3
2	44656788	81150224	0.550				
4	37256112	69659184	0.535				
4	43847544	79501272	0.552	0.547	0.010	1.9	93.6
4	38871888	70217528	0.554				
24	46189684	85047696	0.543				
24	45220524	82579032	0.548	0.539	0.012	2.1	92.3
24	44531940	84701008	0.526				

Table S52. Results of the incubation of (26) in rat plasma over 24.00 h. Three samples were incubated over 24.00 h and measured with MS. The detected areas of (26) peaks were divided by the detected area of IS peaks (DOH). The average of the same time was converted to A(An)/A(IS). The calculated values were referred to the recovered (26) peak area at t = 0.00 h to receive the percentage of the analyte (26) (n = 3).

	Peak area						
**Time [h]**	**(26)**	**Donepezil**	**A(Compound)/A(Standard)**	**A/A mean**	**Standard dev.**	**Var. [%]**	**Remaining compound [%]**
0	38846856	72536272	0.536				
0	36105024	69655096	0.518	0.523	0.011	2.1	100.0
0	34675392	67350264	0.515				
0.5	38219740	76490040	0.500				
0.5	31378086	60020860	0.523	0.505	0.016	3.1	96.6
0.5	36692176	74405168	0.493				
1	30615608	63146912	0.485				
1	40152664	77809608	0.516	0.499	0.016	3.2	95.5
1	32905284	66234256	0.497				
1.5	47806712	99876592	0.479				
1.5	48581392	97913456	0.496	0.495	0.016	3.3	94.7
1.5	44492980	86988624	0.511				
2	48690940	98467584	0.494				
2	42461764	85975080	0.494	0.494	0.000	0.1	94.5
2	41809048	84629808	0.494				
4	41606952	89323840	0.466				
4	38186984	76577368	0.499	0.481	0.017	3.4	92.0
4	40619612	84872600	0.479				
24	33222588	74760560	0.444				
24	36835112	78846800	0.467	0.466	0.021	4.5	89.1
24	36353276	74730904	0.486				

Table S53. Performance of computational plasma protein-binding predictions against protein-specific biomimetic binding estimates**:** Comparison of predicted plasma protein binding values from preADMET, deepPK, and ADMETlab 3.0 against experimental protein-specific biomimetic binding estimates for human serum albumin (HSA) and α1-acid glycoprotein (AGP), using all 26 analogues and both reference drugs (n = 28). Predictive performance was evaluated using Pearson correlation coefficients, Spearman rank correlations, coefficients of determination (R^2), Lin's concordance correlation coefficient (CCC), mean absolute error (MAE), root mean square error (RMSE), bias, regression slope, and intercept. Bias is reported as predicted minus experimental binding in percentage points. Pearson correlations indicated strong linear associations for all methods across both protein endpoints, although agreement metrics and error estimates varied substantially between platforms. CCC and error-based metrics highlight differences between correlation strength and numerical agreement, particularly where systematic bias or deviations from unity slope were observed.

Endpoint	Method	n	Pearson r (p)	95% CI for r	Spearman rho (p)	R^2	Lin CCC	MAE	RMSE	Bias
HSA	preADMET	28	0.870 (<0.001)	0.736 to 0.939	0.705 (<0.001)	0.757	0.730	7.31	9.78	-6.78
HSA	deepPK	28	0.855 (<0.001)	0.708 to 0.931	0.129 (0.512)	0.731	0.559	12.59	15.26	-12.39
HSA	ADMETlab 3.0	28	0.794 (<0.001)	0.597 to 0.900	0.721 (<0.001)	0.630	0.716	5.74	9.66	-4.49
AGP	preADMET	28	0.794 (<0.001)	0.599 to 0.901	0.052 (0.792)	0.631	0.735	8.15	9.90	+5.03
AGP	deepPK	28	0.861 (<0.001)	0.719 to 0.934	0.468 (0.012)	0.742	0.828	6.12	8.34	-0.59
AGP	ADMETlab 3.0	28	0.710 (<0.001)	0.459 to 0.856	0.259 (0.183)	0.505	0.612	11.16	12.48	+7.32

Table S54. Statistical comparison of experimentally determined HPLC logP values with predictions obtained using different in silico methods. Method performance was evaluated using the Pearson correlation coefficient, mean absolute error (MAE), root mean squared error (RMSE), coefficient of determination ((R^2)), and the number of available predictions ((n)). Higher Pearson (r) and (R^2) values indicate stronger agreement with the experimental data, whereas lower MAE and RMSE values indicate greater predictive accuracy.

**Method**	**Pearson r**	**MAE**	**n**	**RMSE**	**R^2**
Crippen	0.931	0.346	14	0.401	0.867
Viswanadhan	0.906	0.346	14	0.399	0.821
ACD/Chemsketch 2021.2.1	0.812	0.452	28	0.574	0.660
XLOGP	0.752	0.555	28	0.620	0.566
BIOVIA Draw 2022	0.743	0.577	28	0.689	0.552
Broto	0.641	0.370	27	0.656	0.411
MLOGP	0.581	0.688	28	0.841	0.337
SILICO-IT	0.578	1.168	28	1.446	0.334
